# ACE2-binding exposes the SARS-CoV-2 fusion peptide to broadly neutralizing coronavirus antibodies

**DOI:** 10.1126/science.abq2679

**Published:** 2022-07-12

**Authors:** Jun Siong Low, Josipa Jerak, M. Alejandra Tortorici, Matthew McCallum, Dora Pinto, Antonino Cassotta, Mathilde Foglierini, Federico Mele, Rana Abdelnabi, Birgit Weynand, Julia Noack, Martin Montiel-Ruiz, Siro Bianchi, Fabio Benigni, Nicole Sprugasci, Anshu Joshi, John E. Bowen, Cameron Stewart, Megi Rexhepaj, Alexandra C. Walls, David Jarrossay, Diego Morone, Philipp Paparoditis, Christian Garzoni, Paolo Ferrari, Alessandro Ceschi, Johan Neyts, Lisa A. Purcell, Gyorgy Snell, Davide Corti, Antonio Lanzavecchia, David Veesler, Federica Sallusto

**Affiliations:** ^1^ Institute for Research in Biomedicine, Università della Svizzera Italiana, 6500 Bellinzona, Switzerland.; ^2^ Institute of Microbiology, ETH Zürich, 8093 Zurich, Switzerland.; ^3^ Department of Biochemistry, University of Washington, Seattle, WA 98195, USA.; ^4^ Humabs BioMed SA (subsidiary of Vir Biotechnology), 6500 Bellinzona, Switzerland.; ^5^ KU Leuven Department of Microbiology, Immunology and Transplantation, Rega Institute for Medical Research, Laboratory of Virology and Chemotherapy, B-3000 Leuven, Belgium.; ^6^ KU Leuven Department of Imaging and Pathology, Translational Cell and Tissue Research, B-3000 Leuven, Belgium.; ^7^ Vir Biotechnology, San Francisco, CA 94158, USA.; ^8^ Howard Hughes Medical Institute, Seattle, WA 98195, USA.; ^9^ Clinic of Internal Medicine and Infectious Diseases, Clinica Luganese Moncucco; 6900 Lugano, Switzerland.; ^10^ Faculty of Biomedical Sciences, Università della Svizzera Italiana, 6900 Lugano, Switzerland.; ^11^ Department of Internal Medicine, Ente Ospedaliero Cantonale, 6500 Bellinzona, Switzerland.; ^12^ Prince of Wales Hospital Clinical School, University of New South Wales, Sydney, New South Wales 2052, Australia.; ^13^ Division of Clinical Pharmacology and Toxicology, Institute of Pharmacological Sciences of Southern Switzerland, Ente Ospedaliero Cantonale, 6900 Lugano, Switzerland.; ^14^ Clinical Trial Unit, Ente Ospedaliero Cantonale, 6500 Bellinzona, Switzerland.; ^15^ Department of Clinical Pharmacology and Toxicology, University Hospital Zurich, 8091 Zurich, Switzerland.; ^16^ Global Virus Network, Baltimore, MD 21201, USA.; ^17^ National Institute of Molecular Genetics, 20122 Milano, Italy.

## Abstract

The coronavirus spike (S) glycoprotein attaches to host receptors and mediates viral fusion. Using a broad screening approach, we isolated from severe acute respiratory syndrome coronavirus 2 (SARS-CoV-2) immune donors seven monoclonal antibodies (mAbs) that bind to all human-infecting coronavirus S proteins. This class of mAbs recognize the fusion peptide and acquire affinity and breadth through somatic mutations. Despite targeting a conserved motif, only some mAbs show broad neutralizing activity in vitro against alpha- and beta-coronaviruses, including animal coronavirus WIV-1 and PDF-2180. Two selected mAbs also neutralize Omicron BA.1 and BA.2 authentic viruses and reduce viral burden and pathology in vivo. Structural and functional analyses show that the fusion peptide-specific mAbs bind with different modalities to a cryptic epitope, which is hidden in prefusion stabilized S, and becomes exposed upon binding of angiotensin-converting enzyme 2 (ACE2) or ACE2-mimicking mAbs.

The Orthocoronavirinae subfamily of coronaviruses comprises four genera: *alphacoronavirus*, *betacoronavirus*, *gammacoronavirus* and *deltacoronavirus*. Alphacoronaviruses NL63 and 229E and betacoronaviruses OC43, HKU1, SARS-CoV-2, SARS-CoV and MERS-CoV account for almost all human coronavirus (hCoV) infections, with sporadic infections attributed to CCoV-HuPn-2018 and porcine deltacoronavirus (PDCoV) (*1*–*3*). The spike (S) glycoprotein, which binds to various host receptors for viral entry (table S1), is composed of the S_1_ and S_2_ subunits, is highly divergent, with only ~30% sequence identity between alpha- and betacoronaviruses and is the main target of neutralizing antibodies (*4*–*7*). Previous studies have described neutralizing monoclonal antibodies (mAbs) that cross-react amongst sarbecoviruses by targeting the receptor binding domain (RBD) (*8*–*14*) or more broadly across betacoronaviruses by targeting the stem helix (*15*–*20*). However, mAbs neutralizing both alpha- and betacoronaviruses have not been reported. Broadly neutralizing mAbs, as exemplified by those targeting influenza viruses or HIV-1 (*21*–*26*), can potentially be used for prophylaxis or therapy and to guide the design of vaccines eliciting broadly protective immunity (*27*, *28*).

## Isolation of broadly reactive coronavirus mAbs from convalescent and vaccinated individuals

**Fig. 1.**
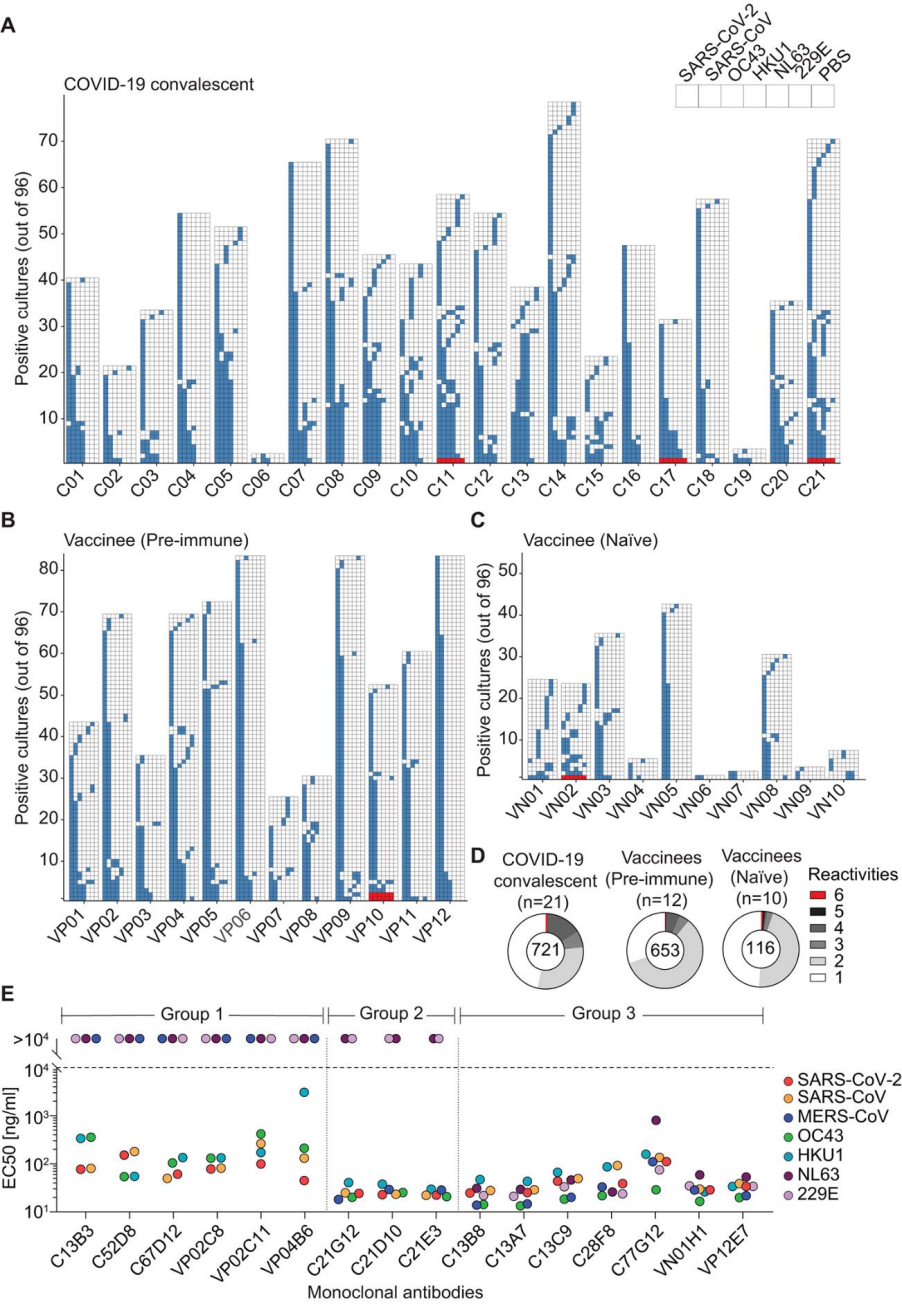
Rare broadly reactive memory B cells are elicited upon natural infection or vaccination. (**A** to **D**) Total PBMCs from COVID-19 convalescents (C) (**A**), vaccinees with prior infection (pre-immune) (VP) (**B**), and vaccinees without prior infection (naïve) (VN) (**C**) (table S5) were plated in replicate 96 wells (10^4^ cells/well) and stimulated with TLR agonist R848 (2.5 μg/ml) in the presence of IL-2 (1,000 U/ml). Twelve days later, the supernatant of each culture was screened, in parallel, for the specificities of the secreted IgG antibodies to commercially available hCoV S proteins from SARS-CoV-2, SARS-CoV, OC43, HKU1, NL63 and 229E by ELISA. The skyline plot provides a detailed view of the specificities of each culture well (represented in rows) to the respective antigens (in subcolumn) from each donor (in column). The order of the antigens is indicated in the legend and uncoated plates (PBS) were used as control. If OD 405nm value exceeds the cut-off value determined by average OD 405nm of PBS wells + 4*standard deviation, the culture was considered reactive to the antigen and indicated with colored cells. Only cultures exhibiting reactivity to at least one antigen are shown. Red cells highlight the six cultures exhibiting reactivity to all alpha and beta hCoV S proteins tested. (**D**) Cross-reactivity patterns of SARS-CoV-2 S positive cultures from (**A** to **C**) shown as pie charts. The total number of SARS-CoV-2 S-positive cultures from each cohort is indicated at the center of the pie. (**E**) Using a two-step screening strategy as depicted in fig. S1, 16 mAbs that cross-reacted with multiple hCoV S proteins were isolated from 10 donors (C series from convalescent donors, VP and VN series from pre-immune and naïve vaccinees). Shown are EC50 values to the respective commercially available hCoV S protein, measured by ELISA. mAbs are grouped based on the reactivity patterns. Shown is one representative experiment out of at least 2 performed. Group 2 mAbs C21G12, C21D10, and C21E3 were described in a separate study (*15*) (designated P34G12, P34D10, and P34E3, respectively).

To search for antibodies that cross-react with human-infecting alpha- and betacoronaviruses, we stimulated under limiting conditions, total peripheral blood mononuclear cells (PBMCs) from SARS-CoV-2 immune donors, in the presence of the TLR7/8 agonist R848 and IL-2, which selectively induce the proliferation and differentiation of memory B cells (*29*). On day 12, the specificities of IgGs secreted in the culture supernatants were tested by enzyme-linked immunosorbent assay (ELISA) against a panel of recombinant S proteins from alpha and beta hCoV (Fig. 1, A to C). The number of SARS-CoV-2 IgG-positive cultures was generally higher in COVID-19 convalescent patients and in SARS-CoV-2 vaccinees with prior infection (pre-immune) as compared to vaccinees without prior infection (naïve). Most SARS-CoV-2 S IgG-positive cultures were either monospecific or cross-reactive with the closely related SARS-CoV S, while a small fraction was also reactive with OC43 and HKU1 S (Fig. 1, A to D), consistent with previous serological analyses (*17* *,* *30* *,* *31*). Six cultures (out of >4,000) from 5 individuals (out of 43) cross-reacted with all alpha and beta hCoV S proteins tested (Fig. 1, A to D), suggesting that memory B cells producing broadly reactive coronavirus antibodies might exist at very low frequency.

To isolate broadly reactive coronavirus mAbs, we combined the screening of polyclonally-activated memory B cells with the sorting and cloning of antibody secreting cells to retrieve paired heavy and light chain sequences (fig. S1). Using this approach, we isolated 16 SARS-CoV-2-S-specific mAbs that cross-reacted with various hCoV S proteins (Fig. 1E and fig. S2A). Six mAbs (Group 1) cross-reacted with the betacoronaviruses SARS-CoV, SARS-CoV-2, OC43 and HKU1 S proteins with EC50 values ranging from 45 ng/ml to 3,000 ng/ml. Three mAbs (Group 2) cross-reacted with high affinity to all beta hCoV S proteins, with EC50 values ranging from 18 ng/ml to 40 ng/ml (Fig. 1E). These mAbs were found to target the stem helix region and were described in a separate study (*15*). The remaining seven mAbs (Group 3) exhibited the broadest cross-reactivity to both alpha and beta hCoV S proteins with EC50 values ranging from 29 ng/ml to 800 ng/ml (Fig. 1E). These broadly reactive mAbs, which are the focus of the present study, were isolated from convalescent or vaccinated individuals, use different V genes (except for C13B8 and C13A7 that are clonally related) and displayed a high load of somatic mutations (7-14% in VH, 2-8% in VL at the nucleotide level; table S2). These results illustrate the utility of a simple high-throughput method based on multiple parallel screening steps of memory B cells to isolate broadly reactive coronavirus mAbs.

## Broadly reactive mAbs bind to the fusion peptide and acquire affinity and breadth through somatic mutations

**Fig. 2.**
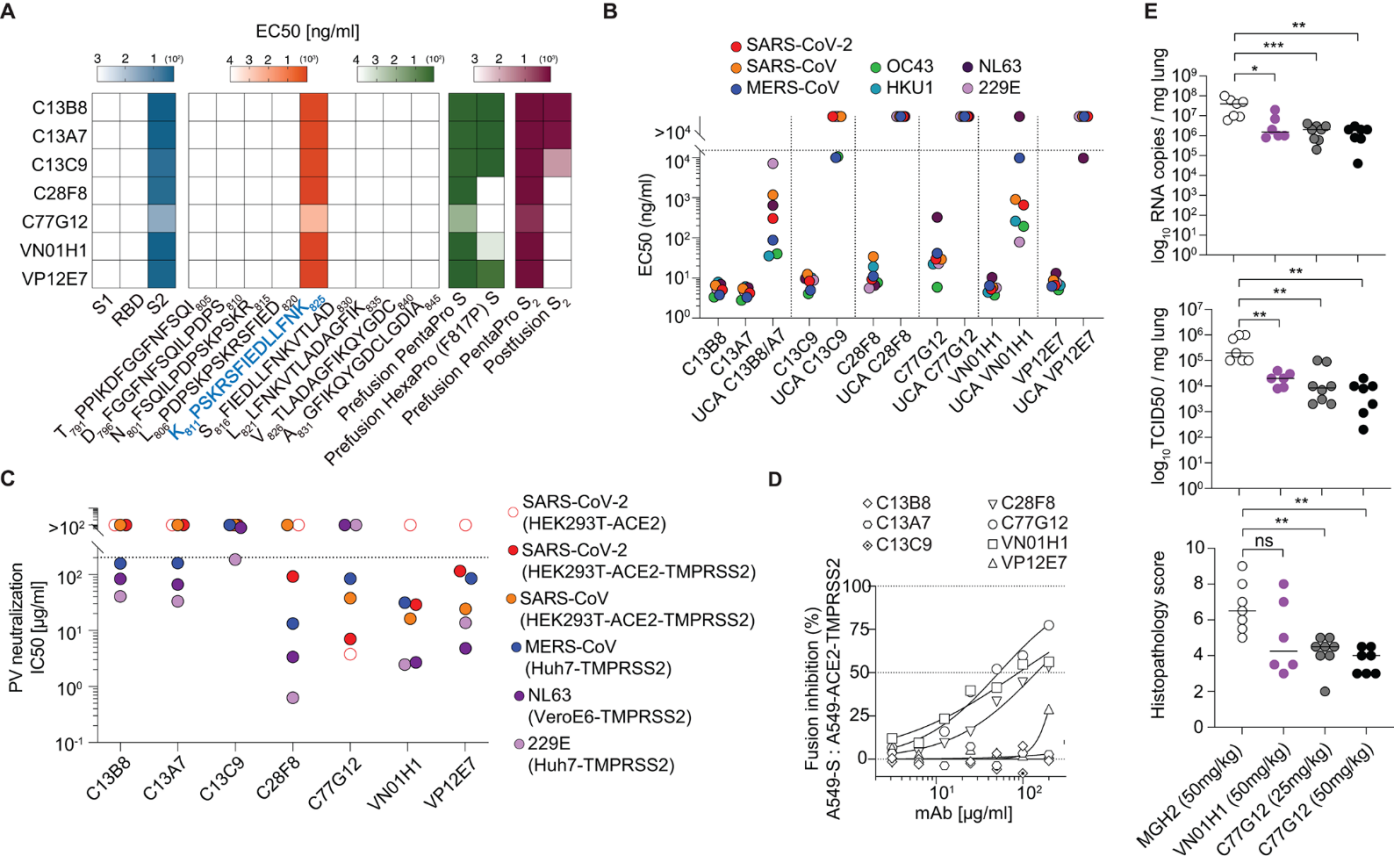
Broadly reactive fusion peptide-targeted mAbs acquire affinity and breadth through somatic mutations. (**A**) Binding profiles of the seven Group 3 mAbs. Epitope mapping was performed using commercially available SARS-CoV-2 protein domains (S_1_, RBD, S_2_), 15-mer overlapping synthetic peptides with the indicated sequences, prefusion HexaPro (F817P) S, prefusion PentaPro (F817) S, prefusion PentaPro (F817) S_2_ and postfusion S_2_ coated onto plastic and assessed by ELISA (log_10_ EC50 shown). The mAbs all recognize the fusion peptide sequence highlighted in blue. (**B**) Binding evaluation of Group3 mAbs and their respective germline reverted unmutated common ancestors (UCAs) to different hCoV S proteins. C13B8 and C13A7 are sister clones and thus share a single UCA. One representative experiment out of at least two experiments is shown. (**C**) Half-maximum inhibitory concentrations (IC_50_s) of Group 3 mAbs against SARS-CoV-2, SARS-CoV, MERS-CoV, NL63 and 229E pseudoviruses in the indicated target cell lines. For each hCoV pseudotyped assay, all mAbs were compared in parallel. One representative experiment out of at least two experiments is shown. (**D**) Group 3 mAbs were assessed for their ability to inhibit the fusion of SARS-CoV-2 S-expressing (A549-S) and ACE2-TMPRSS2-expressing (A549-ACE2-TMPRSS2) A549 cells. Inhibition of fusion values are normalized to the percentage of fusion without mAb (100%) and to that of fusion of non-transfected cells (0%). One representative experiment out of two is shown. (**E**) The prophylactic efficacy of VN01H1 (50 mg/kg), C77G12 (25 mg/kg and 50 mg/kg) and negative control anti-malaria mAb MGH2 (50 mg/kg) were tested in hamsters challenged with the SARS-CoV-2 P.1 (Gamma) variant of concern. Viral RNA loads (top), replicating virus titers (middle), and histopathological scores (bottom) are shown. ns *P* > 0.05, *P ≤ 0.05, **P ≤ 0.01, ***P ≤ 0.001, Mann-Whitney test corrected using Bonferroni multiple comparison.

Using SARS-CoV-2 S_1_, RBD and S_2_ proteins, as well as 15-mer linear peptides covering the entire S sequence, the specificities of all seven Group 3 mAbs were mapped to the K_811_PSKRSFIEDLLFNK_825_ sequence in the SARS-CoV-2 S_2_ subunit (Fig. 2A). This sequence spans the S_2_’ cleavage site (R815) and the fusion peptide N-terminal region, which is essential for membrane fusion (*32*) and is highly conserved among all genera of the Orthocoronavirinae subfamily, including deltacoronavirus PDCoV and gammacoronavirus IBV, as well as all SARS-CoV-2 variants sequenced to date (fig. S3, A to C). Most of the seven Group 3 mAbs bound more tightly to the prefusion SARS-CoV-2 S trimer without stabilizing proline substitution in the fusion peptide (PentaPro: F817) relative to HexaPro (F817P) (*33*) and all of them bound with greater avidity to prefusion SARS-CoV-2 S_2_ compared to postfusion S_2_ (Fig. 2A and fig. S2D).

To explore the ontogeny of these fusion peptide-specific mAbs, we compared the binding properties of mature mAbs to their unmutated common ancestors (UCAs) (Fig. 2B and fig. S2B). The UCAs of C13B8, C13A7 (the two clonally related mAbs) and VN01H1 exhibited broad reactivity with hCoV S proteins but had lower affinity than their mature counterparts. In contrast, the C13C9 UCA bound only to the beta hCoV OC43 and MERS-CoV S, whereas the VP12E7 UCA bound only to the alpha hCoV NL63 S, with low affinity in both cases. Finally, the UCAs of C28F8 and C77G12 did not bind to any hCoV S proteins tested. Given the lack of a common V gene usage, these findings suggest that broadly reactive fusion peptide-specific mAbs can mature through multiple pathways and acquire high affinity and cross-reactivity through somatic mutations, possibly because of priming by endemic coronavirus infection followed by SARS-CoV-2 boosting upon infection or vaccination.

Considering the high conservation of the fusion peptide region, we next assessed if broad reactivity is a property shared by most fusion peptide-specific mAbs. IgGs secreted upon polyclonal activation of memory B cells from 71 convalescent individuals were screened for binding to alpha and beta hCoV fusion peptide pool as well as to SARS-CoV-2 S protein. Cultures producing fusion peptide-specific mAbs were detected at low frequency and only in 19 individuals (fig. S4A). Although nearly all fusion peptide-reactive mAbs bound to SARS-CoV-2 S, only 9 out of 30 were broadly reactive and the remaining mAbs showed different degrees of cross-reactivity (fig. S4B). Thus, although the coronavirus S fusion peptide is conserved (*34*–*39*), broad reactivity is the property of a minority of fusion peptide-specific mAbs, consistent with the observation that only three out of the seven Group 3 mAbs cross-reacted with the PDCoV and IBV fusion peptides (fig. S3C).

## Anti-fusion peptide mAbs have heterogeneous neutralizing activities and can reduce viral burden in vivo

We next tested the neutralizing activity of all seven Group 3 mAbs against pseudotyped viruses carrying S proteins of alpha- and betacoronaviruses. Despite binding to the same motif, these mAbs exhibited distinct neutralizing potencies. Most notably, VN01H1 and VP12E7 neutralized all hCoV S pseudotyped viruses tested (SARS-CoV-2, SARS-CoV, MERS-CoV, NL63 and 229E), as well as bat sarbecovirus WIV-1 S pseudotyped viruses (Fig. 2C and fig. S5, A to C). In addition, VN01H1 also inhibited bat merbecovirus PDF-2180 (*40*) S-mediated entry into cells (fig. S5B). C77G12 neutralized SARS-CoV-2 with the highest relative potency and all betacoronavirus S pseudotype viruses tested (Fig. 2C and fig. S5, A to C). SARS-CoV-2 neutralizing activity of VN01H1 and C77G12 was reduced by the addition of soluble fusion peptide (fig. S5D). Furthermore, VN01H1, C77G12 and C28F8 also inhibited SARS-CoV-2 S-mediated cell-cell fusion (Fig. 2D), suggesting that fusion peptide-specific mAbs could prevent S proteolytic activation or fusogenic rearrangements, thereby inhibiting membrane fusion and viral entry.

We next assessed the protective efficacy of the VN01H1 and C77G12 mAbs in vivo. In the Syrian hamster model of SARS-CoV-2 P.1 (Gamma) infection, prophylactic administration of either mAb at high doses reduced viral RNA copies and lung titers, and ameliorated lung pathology at statistically significant levels (Fig. 2E). These findings demonstrate that fusion peptide-specific mAbs can reduce viral burden in vivo, albeit with moderate potency.

## Structural basis for fusion peptide recognition

To gain insight into the epitope recognized by fusion peptide-specific broadly reactive coronavirus mAbs, we performed substitution scan analysis on the six clonally unrelated mAbs and structural analysis. All mAbs bound to a core motif comprising I_818_EDLLFNK_825_ (fig. S6A), with C28F8, C77G12, VN01H1, and VP12E7 having a 3-amino acid expanded footprint spanning the N-terminal R_815_SF_817_ residues which includes the S_2_’ cleavage site R_815_.

**Fig. 3.**
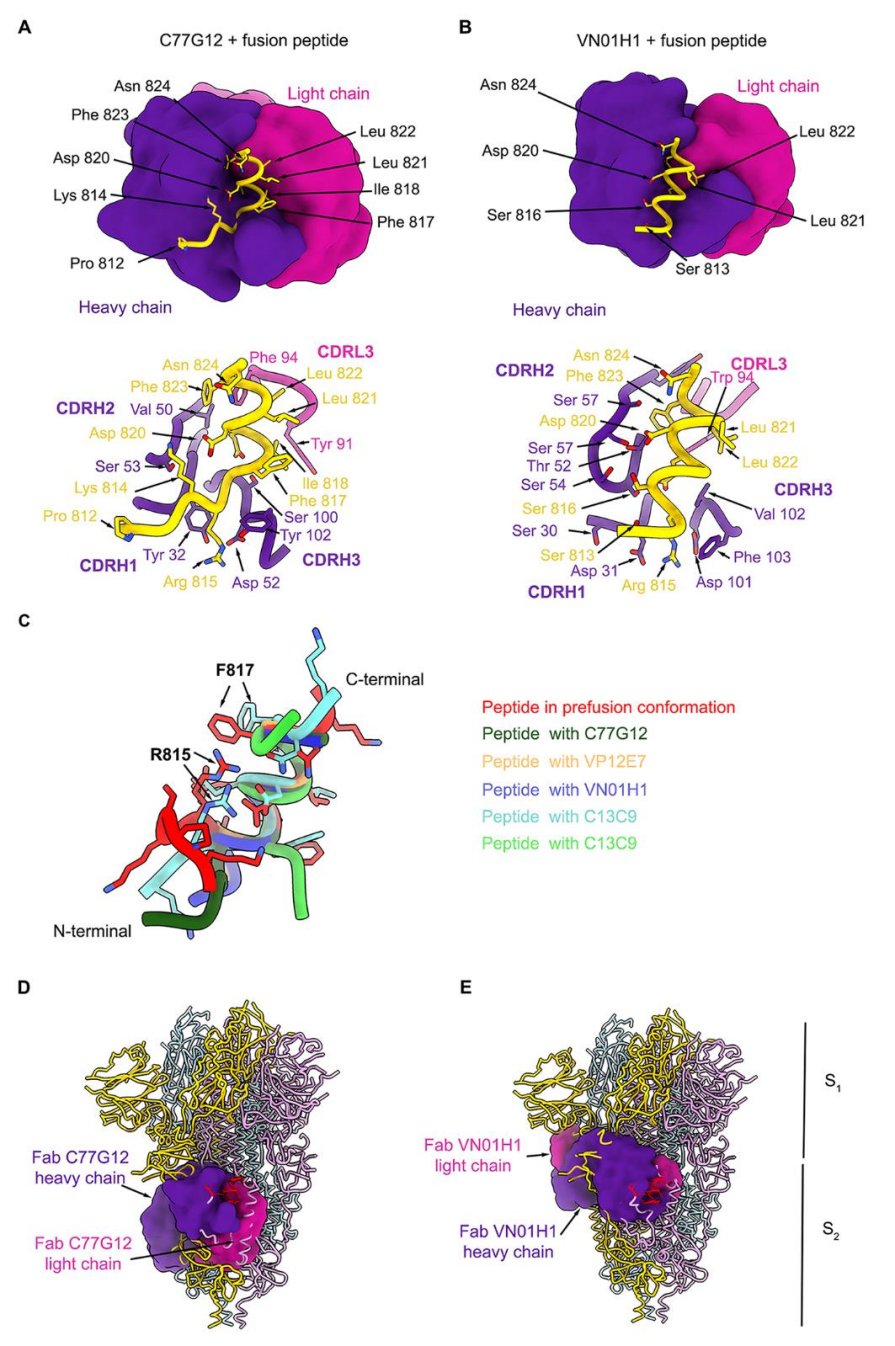
Fusion peptide antibodies target a cryptic epitope. (**A** and **B**) (Top) Surface representation of the crystal structures of the C77G12 (**A**) and VN01H1 (**B**) Fabs in complex with SARS-CoV-2 fusion peptide epitope. (Bottom) Ribbon representation of the corresponding structures highlighting the interactions of Fab heavy and light chain CDRs with the fusion peptide (selected regions are shown for clarity). (**C**), Ribbon representation of the fusion peptides in the Fab-bound complexes superimposed with the fusion peptide in prefusion SARS-CoV-2 S (PDB 6VXX). (**D** and **E**) Superimposition of the C77G12-bound (**D**) or VN01H1-bound (**E**) fusion peptide structures to prefusion SARS-CoV-2 S uncovering the cryptic nature of the epitope. The Fabs are shown as surfaces whereas S is rendered as ribbons. Each SARS-CoV-2 S protomer is colored distinctly (light blue, pink, and gold) and Fab heavy and light chains are colored purple and magenta, respectively.

We determined the crystal structures of five Fabs (C13B8, C13C9, C77G12, VN01H1 and VP12E7) in complex with the K_811_PSKRSFIEDLLFNK_825_ fusion peptide at 2.1 Å, 2.1 Å, 1.7 Å, 1.86 Å and 2.5 Å, resolution, respectively (Fig. 3; fig. S6, B to D; and table S3). All five Fabs bind to overlapping epitopes in the fusion peptide through interactions involving the heavy and light chains. The strict conservation and the conservative substitution of key residues involved in mAb recognition (R_815_, S_816_, I_818,_ E_819_, D_820_, L_821_, L_822_, F_823_, N_824_ and K_825_) across the Orthocoronavirinae subfamily explains the broad cross-reactivity of these fusion peptide-specific mAbs (figs. S3 and S6 and table S4). The overall architecture of the fusion peptide in the C13C9-bound complex structure is most similar to that observed in the prefusion S trimer (PDB 6VXX) (*4*) (Fig. 3C and fig. S6B). In the two structures determined in complex with VN01H1 or VP12E7, the SARS-CoV-2 S fusion peptide adopts a similar conformation, which is distinct from the conformation observed in prefusion SARS-CoV-2 S trimeric structures (*4*) (Fig. 3, B and C, and fig. S6C). Specifically, residues _813_SKR_815_ refold from an extended conformation in prefusion S to an ɑ-helical conformation in the two Fab-bound peptide structures, thereby extending the ɑ-helix found at the N-terminal region of the fusion peptide. In the C77G12-bound complex structure, the fusion peptide residues P_812_-R_815_ adopt an extended conformation, distinct from prefusion S, (Fig. 3, A and C), whereas these residues are disordered in the C13B8-bound structure (Fig. 3C and fig. S6D). The conserved residue R_815_, which is the S_2_’ site of proteolytic processing upon receptor binding for membrane fusion activation, is engaged in electrostatic interactions with the C13C9, VN01H1, VP12E7 and C77G12 Fabs and therefore buried at the interface with their paratopes (table S4). Since pre-incubation of a soluble native-like SARS-CoV-2 S ectodomain trimer with fusion peptide-specific mAbs did not prevent S2X58-induced triggering of fusogenic conformational changes (*41*, *42*) (fig. S7), these mAbs likely inhibit TMPRSS2 cleavage of the S_2_’ site (via steric hindrance) and in turn activation of membrane fusion. Although residue F817 is not part of the epitope (table S4), the F817P substitution present in the HexaPro construct likely prevents the adoption of the extended ɑ-helical conformation observed in the structures bound to VN01H1 or VP12E7 (due to restricted backbone torsion angles), resulting in dampened binding in ELISA (Fig. 2A and fig. S2D).

Superimposition of the Fab-fusion peptide complexes with available prefusion S structure (PDB 6VXX) (*4*, *5*) revealed that the targeted epitope is buried toward the core of the S trimer and is therefore inaccessible (Fig. 3, D and E, and fig. S6, B to D), likely explaining the lack of detectable complexes of Fabs with prefusion S trimers during single particle electron microscopy analysis. Taken together, these findings suggest that the epitope recognized by fusion peptide-specific mAbs is cryptic and may become accessible only transiently (*43*).

## The fusion peptide is unmasked by ACE2 binding

**Fig. 4.**
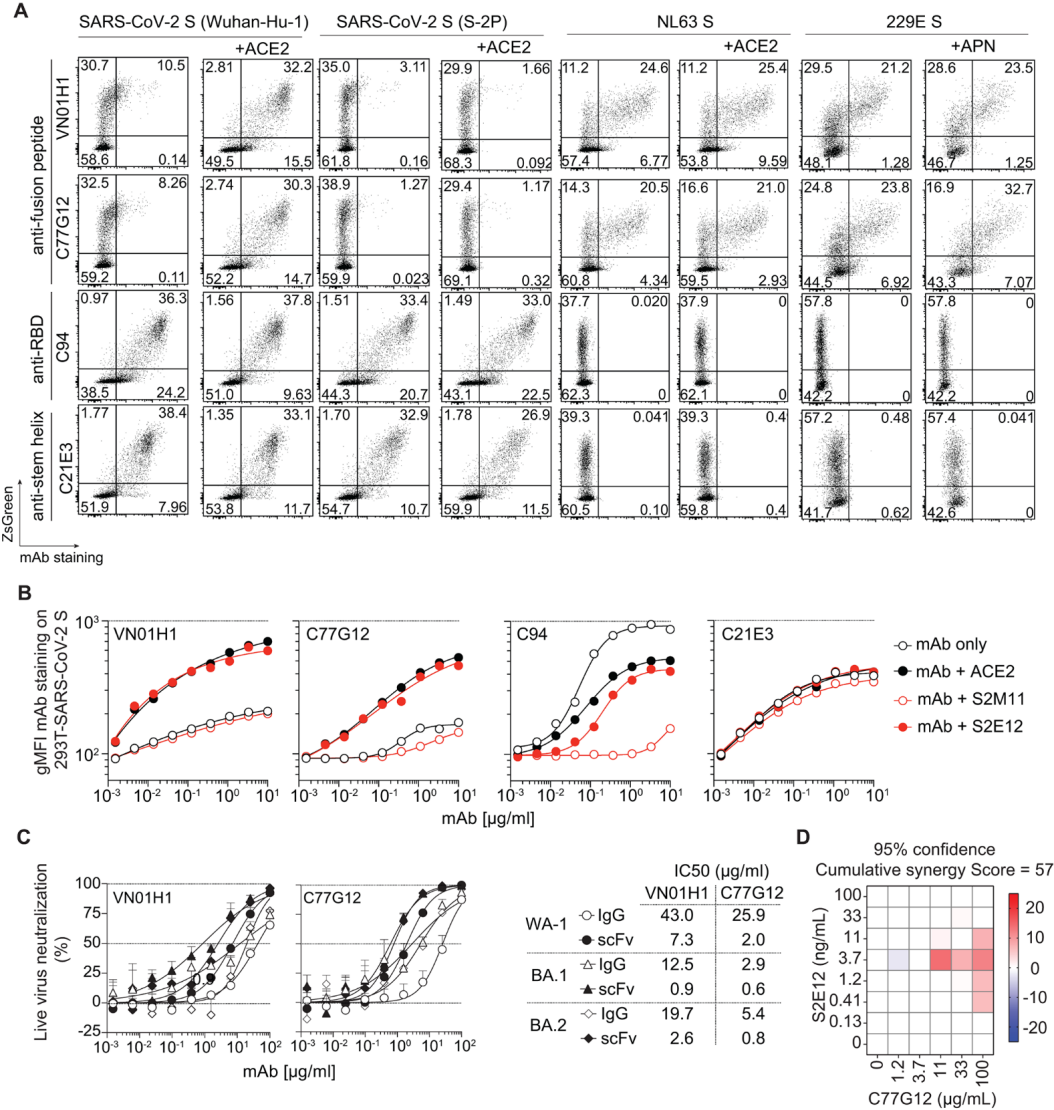
The SARS-CoV-2 fusion peptide is unmasked following ACE2 receptor engagement. (**A**) Binding of fusion peptide-specific mAbs VN01H1 and C77G12 (8 μg/ml), as well as RBD-specific C94 and stem helix-specific C21E3 mAbs (8 μg/ml) on HEK293T cells transiently co-transfected with plasmid encoding ZsGreen and plasmids encoding full length SARS-CoV-2 S Wuhan-Hu-1 (GenBank: NC_045512), SARS-CoV-2 S-2P (K986P, V987P), NL63 S (GenBank: APF29071.1) or 229E S (GenBank: APT69883.1), in the presence or absence of receptor ACE2 or APN, as measured by flow cytometry. (**B**) Titrating doses of fluorescently labeled mAbs were co-incubated with HEK293T cells expressing full-length SARS-CoV-2 S for 2 hours at room temperature in the presence or absence of recombinant ACE2-mFc (27 μg/ml), ACE2-mimicking mAb S2E12 (20 μg/ml) or S2M11 (20 μg/ml), the latter mAb locks the S trimer in a closed conformation. The line in the scatter plot is a reference for the maximum gMFI of staining with control anti-RBD C94 mAb. One representative experiment out of two is shown. (**C**) VN01H1 and C77G12 in full-length IgG or scFv formats were tested for their neutralization of authentic SARS-CoV-2 Washington-1, Omicron BA.1 and BA.2 variants. IC_50_ values are displayed as a table. (**D**) Synergy experiment was performed against SARS-CoV-2 (Wuhan-Hu-1) pseudovirus (0.1 MOI) on VeroE6-TMPRSS2 at indicated concentrations of S2E12 and C77G12 mAbs alone or in combination, in a checkerboard manner. Analysis of mAb synergy was carried out using MacSynergy II (*70*). Synergy plots adjusted with Bonferroni correction at 95% confidence were used for reporting. A synergy score between 50 and 100 was defined as moderate.

To investigate the neutralization mechanism of fusion peptide-specific mAbs, we transfected HEK293T cells to express coronavirus S proteins on their cell surface. All fusion peptide-specific mAbs showed only marginal binding to SARS-CoV-2 S-expressing HEK293T cells, as compared to control mAbs targeting the RBD (C94) (fig. S2C) or the stem helix (C21E3) (Fig. 4A and fig. S8A). However, addition of soluble ACE2 enhanced binding of all fusion peptide mAbs to SARS-CoV-2 S to levels comparable to that of the C94 and C21E3 control mAbs, suggesting that receptor engagement induces a conformational change that exposes the cryptic fusion peptide epitope (Fig. 4, A and B, and fig. S8A). This ACE2-dependent enhancement of binding was also observed with SARS-CoV-2 S harboring a deletion in the polybasic S_1_/S_2_ cleavage site (ΔPRRA) (*44*, *45*) (fig. S8A), suggesting that these mAbs can bind prior to S_1_ shedding or S fusogenic refolding to postfusion state, consistent with the preferential binding to prefusion S_2_ relative to postfusion S_2_ (fig. S2A). However, the 2P (K986P, V987P) prefusion-stabilizing mutations (*4*, *5*) that lay outside of the epitope (Fig. 4A and fig. S8A), inhibited ACE2-mediated enhancement of mAb binding, implying an impediment of receptor-induced allosteric conformational changes, in line with recent findings (*46*). Enhanced binding of fusion peptide-specific mAbs was also observed with SARS-CoV S- and MERS-CoV S-expressing HEK293T cells in the presence of ACE2 (*47*) and DPP4 (*48*). In contrast, these mAbs bound efficiently to HEK293T cells displaying the alphacoronaviruses NL63 and 229E S, independently of receptor engagement by ACE2 (*49*) or APN (*50*), respectively (Fig. 4A and fig. S8A). We observed that VN01H1 and C77G12 neutralized authentic SARS-CoV-2 Omicron BA.1 and BA.2 more potently than Wuhan-Hu-1, likely due to increased accessibility of the fusion peptide in Omicron S (*46*). Furthermore, the neutralization potencies of VN01H1 and C77G12 could be further improved by engineering mAbs with smaller formats (such as scFv), suggesting possible limited accessibility of the fusion peptide region (Fig. 4C).

It was previously shown that certain mAbs can mimic ACE2 binding and trigger fusogenic activity (*51*, *52*). Indeed, the addition of S2E12, an ACE2-mimicking mAb, but not S2M11, a mAb which locks SARS-CoV-2 S trimer in the closed state (*51*) enhanced binding of the fusion peptide mAbs (Fig. 4B). Consistent with this finding, we observed a synergy in pseudovirus neutralization when C77G12 was used in combination with S2E12 (Fig. 4D), suggesting that fusion-peptide mAbs may be more effective in the context of a polyclonal antibody response against the RBD. Collectively, these results indicate that the fusion peptide epitope only becomes accessible upon receptor-induced S conformational changes in sarbecovirus SARS-CoV-2, SARS-CoV and merbecovirus MERS-CoV, whereas it is more readily accessible in the alphacoronaviruses 229E and NL63, possibly due to molecular breathing to different extents in these S trimers.

## Discussion

Using a high-throughput screening of memory B cells from immune donors with a panel of S proteins, we identified a new class of mAbs that target the fusion peptide region. These mAbs have different binding modalities, some of which exhibit unprecedented cross-reactivity to the fusion peptide of all four coronavirus genera. The finding that the UCAs of some of these mAbs bind preferentially to common cold coronaviruses and that the mAbs acquire affinity and breadth through somatic mutations suggest that their elicitation may require multiple rounds of selection, possibly by heterologous coronavirus infections. A complex developmental pathway has been reported for HIV-1 neutralizing mAbs and may be a general requirement for mAbs that recognize highly conserved epitopes (*53*, *54*).

Previous studies have identified serum antibodies binding to the fusion peptide of SARS-CoV-2 and showed, through depletion or peptide inhibition experiments, that such antibodies can contribute to the serum neutralizing activity in a polyclonal setting (*34*–*38*). We show here that some fusion peptide-specific mAbs have direct neutralizing activity in vitro against alpha- and beta-coronaviruses and can ameliorate pathology and viral burden in vivo at high doses. Although the neutralizing activity of these mAbs is low when used alone, it is possible that in the context of a polyclonal response they may synergize with other antibodies that favor the exposure of the fusion peptide region, as shown here with S2E12.

The biochemical events associated with S_2_’ proteolytic processing for SARS-CoV, SARS-CoV-2 and MERS-CoV and that are critical for unmasking the fusion peptide region have been described previously (*55*–*59*). The mAbs isolated in this study provide tools to characterize the S allosteric conformational changes triggered by receptor binding or by receptor-mimicking mAbs (*51*, *52*), a process that has thus far eluded structural definition. These events lead to exposure of the fusion peptide region for proteolytic cleavage by TMPRSS2 or endosomal cathepsins preceding membrane fusion (*32*). The finding that this region is readily accessible in alphacoronaviruses is consistent with the higher neutralizing activity of some fusion peptide-specific mAbs against NL63 and 229E and proposes that such conformational change might occur spontaneously as a consequence of molecular breathing.

Broadly neutralizing mAbs recognizing the fusion peptide region of influenza virus hemagglutinin and HIV-1 gp120 have guided the design of universal vaccine candidates against these highly variable pathogens (*60*–*66*). Similarly, the conservation of the fusion peptide region across the *Orthocoronavirinae* subfamily provides a rationale to develop a vaccine capable of inducing fusion peptide-specific responses that may be less prone to viral escape due to the potential fitness loss of escape mutants (*67*, *68*). The finding that prefusion stabilizing 2P mutations (*4*, *5*) abolishes receptor-induced exposure of the fusion peptide suggests that current mRNA vaccines may be suboptimal in inducing antibody responses against this region. Broadly neutralizing coronavirus mAbs described in this study may be used as probes to design immunogens that can better unmask the fusion peptide region and elicit broadly protective antibody responses. Interestingly, the fusion peptide region was also found to stimulate broadly reactive CD4 T cells (*69*), providing a cue for intramolecular help in the generation of such antibodies. Collectively, our study identifies neutralizing mAbs with unprecedented breadth and sheds light on a receptor-triggered conformational change that limits the immunogenicity of this conserved region, with potential impact on vaccine design.

## Supplementary Materials

### This PDF file includes:

Materials and Methods Figs. S1 to S8 Tables S1 to S5 References (*71* *–* *106*)

### Other Supplementary Material for this manuscript includes the following:

MDAR Reproducibility Checklist

## References

[1] A. N. Vlasova , A. Diaz , D. Damtie , L. Xiu , T.-H. Toh , J. S.-Y. Lee , L. J. Saif , G. C. Gray , Novel canine coronavirus isolated from a hospitalized patient with pneumonia in East Malaysia. Clin. Infect. Dis. 74, 446–454 (2022). 10.1093/cid/ciab456 34013321 PMC8194511

[2] J. A. Lednicky , M. S. Tagliamonte , S. K. White , M. A. Elbadry , M. M. Alam , C. J. Stephenson , T. S. Bonny , J. C. Loeb , T. Telisma , S. Chavannes , D. A. Ostrov , C. Mavian , V. M. Beau De Rochars , M. Salemi , J. G. Morris Jr ., Independent infections of porcine deltacoronavirus among Haitian children. Nature 600, 133–137 (2021). 10.1038/s41586-021-04111-z 34789872 PMC8636265

[3] M. A. Tortorici , A. C. Walls , A. Joshi , Y.-J. Park , R. T. Eguia , M. C. Miranda , E. Kepl , A. Dosey , T. Stevens-Ayers , M. J. Boeckh , A. Telenti , A. Lanzavecchia , N. P. King , D. Corti , J. D. Bloom , D. Veesler , Structure, receptor recognition, and antigenicity of the human coronavirus CCoV-HuPn-2018 spike glycoprotein. Cell 185, 2279–2291.e17 (2022). 10.1016/j.cell.2022.05.019 35700730 PMC9135795

[4] A. C. Walls , Y.-J. Park , M. A. Tortorici , A. Wall , A. T. McGuire , D. Veesler , Structure, function, and antigenicity of the SARS-CoV-2 spike glycoprotein. Cell 181, 281–292.e6 (2020). 10.1016/j.cell.2020.02.058 32155444 PMC7102599

[5] D. Wrapp , N. Wang , K. S. Corbett , J. A. Goldsmith , C.-L. Hsieh , O. Abiona , B. S. Graham , J. S. McLellan , Cryo-EM structure of the 2019-nCoV spike in the prefusion conformation. Science 367, 1260–1263 (2020). 10.1126/science.abb2507 32075877 PMC7164637

[6] L. Piccoli , Y. J. Park , M. A. Tortorici , N. Czudnochowski , A. C. Walls , M. Beltramello , C. Silacci-Fregni , D. Pinto , L. E. Rosen , J. E. Bowen , O. J. Acton , S. Jaconi , B. Guarino , A. Minola , F. Zatta , N. Sprugasci , J. Bassi , A. Peter , A. De Marco , J. C. Nix , F. Mele , S. Jovic , B. F. Rodriguez , S. V. Gupta , F. Jin , G. Piumatti , G. Lo Presti , A. F. Pellanda , M. Biggiogero , M. Tarkowski , M. S. Pizzuto , E. Cameroni , C. Havenar-Daughton , M. Smithey , D. Hong , V. Lepori , E. Albanese , A. Ceschi , E. Bernasconi , L. Elzi , P. Ferrari , C. Garzoni , A. Riva , G. Snell , F. Sallusto , K. Fink , H. W. Virgin , A. Lanzavecchia , D. Corti , D. Veesler , Mapping neutralizing and immunodominant sites on the SARS-CoV-2 spike receptor-binding domain by structure-guided high-resolution serology. Cell 183, 1024–1042.e21 (2020). 10.1016/j.cell.2020.09.037 32991844 PMC7494283

[7] A. J. Greaney , T. N. Starr , P. Gilchuk , S. J. Zost , E. Binshtein , A. N. Loes , S. K. Hilton , J. Huddleston , R. Eguia , K. H. D. Crawford , A. S. Dingens , R. S. Nargi , R. E. Sutton , N. Suryadevara , P. W. Rothlauf , Z. Liu , S. P. J. Whelan , R. H. Carnahan , J. E. Crowe Jr ., J. D. Bloom , Complete mapping of mutations to the SARS-CoV-2 spike receptor-binding domain that escape antibody recognition. Cell Host Microbe 29, 44–57.e9 (2021). 10.1016/j.chom.2020.11.007 33259788 PMC7676316

[8] A. Z. Wec , D. Wrapp , A. S. Herbert , D. P. Maurer , D. Haslwanter , M. Sakharkar , R. K. Jangra , M. E. Dieterle , A. Lilov , D. Huang , L. V. Tse , N. V. Johnson , C.-L. Hsieh , N. Wang , J. H. Nett , E. Champney , I. Burnina , M. Brown , S. Lin , M. Sinclair , C. Johnson , S. Pudi , R. Bortz III , A. S. Wirchnianski , E. Laudermilch , C. Florez , J. M. Fels , C. M. O’Brien , B. S. Graham , D. Nemazee , D. R. Burton , R. S. Baric , J. E. Voss , K. Chandran , J. M. Dye , J. S. McLellan , L. M. Walker , Broad neutralization of SARS-related viruses by human monoclonal antibodies. Science 369, 731–736 (2020). 10.1126/science.abc7424 32540900 PMC7299279

[9] D. Pinto , Y. J. Park , M. Beltramello , A. C. Walls , M. A. Tortorici , S. Bianchi , S. Jaconi , K. Culap , F. Zatta , A. De Marco , A. Peter , B. Guarino , R. Spreafico , E. Cameroni , J. B. Case , R. E. Chen , C. Havenar-Daughton , G. Snell , A. Telenti , H. W. Virgin , A. Lanzavecchia , M. S. Diamond , K. Fink , D. Veesler , D. Corti , Cross-neutralization of SARS-CoV-2 by a human monoclonal SARS-CoV antibody. Nature 583, 290–295 (2020). 10.1038/s41586-020-2349-y 32422645

[10] T. N. Starr , N. Czudnochowski , Z. Liu , F. Zatta , Y.-J. Park , A. Addetia , D. Pinto , M. Beltramello , P. Hernandez , A. J. Greaney , R. Marzi , W. G. Glass , I. Zhang , A. S. Dingens , J. E. Bowen , M. A. Tortorici , A. C. Walls , J. A. Wojcechowskyj , A. De Marco , L. E. Rosen , J. Zhou , M. Montiel-Ruiz , H. Kaiser , J. R. Dillen , H. Tucker , J. Bassi , C. Silacci-Fregni , M. P. Housley , J. di Iulio , G. Lombardo , M. Agostini , N. Sprugasci , K. Culap , S. Jaconi , M. Meury , E. Dellota Jr ., R. Abdelnabi , S. C. Foo , E. Cameroni , S. Stumpf , T. I. Croll , J. C. Nix , C. Havenar-Daughton , L. Piccoli , F. Benigni , J. Neyts , A. Telenti , F. A. Lempp , M. S. Pizzuto , J. D. Chodera , C. M. Hebner , H. W. Virgin , S. P. J. Whelan , D. Veesler , D. Corti , J. D. Bloom , G. Snell , SARS-CoV-2 RBD antibodies that maximize breadth and resistance to escape. Nature 597, 97–102 (2021). 10.1038/s41586-021-03807-6 34261126 PMC9282883

[11] C. A. Jette , A. A. Cohen , P. N. P. Gnanapragasam , F. Muecksch , Y. E. Lee , K. E. Huey-Tubman , F. Schmidt , T. Hatziioannou , P. D. Bieniasz , M. C. Nussenzweig , A. P. West Jr ., J. R. Keeffe , P. J. Bjorkman , C. O. Barnes , Broad cross-reactivity across sarbecoviruses exhibited by a subset of COVID-19 donor-derived neutralizing antibodies. Cell Rep. 36, 109760 (2021). 10.1016/j.celrep.2021.109760 34534459 PMC8423902

[12] M. A. Tortorici , N. Czudnochowski , T. N. Starr , R. Marzi , A. C. Walls , F. Zatta , J. E. Bowen , S. Jaconi , J. Di Iulio , Z. Wang , A. De Marco , S. K. Zepeda , D. Pinto , Z. Liu , M. Beltramello , I. Bartha , M. P. Housley , F. A. Lempp , L. E. Rosen , E. Dellota Jr ., H. Kaiser , M. Montiel-Ruiz , J. Zhou , A. Addetia , B. Guarino , K. Culap , N. Sprugasci , C. Saliba , E. Vetti , I. Giacchetto-Sasselli , C. S. Fregni , R. Abdelnabi , S. C. Foo , C. Havenar-Daughton , M. A. Schmid , F. Benigni , E. Cameroni , J. Neyts , A. Telenti , H. W. Virgin , S. P. J. Whelan , G. Snell , J. D. Bloom , D. Corti , D. Veesler , M. S. Pizzuto , Broad sarbecovirus neutralization by a human monoclonal antibody. Nature 597, 103–108 (2021). 10.1038/s41586-021-03817-4 34280951 PMC9341430

[13] Y.-J. Park , A. De Marco , T. N. Starr , Z. Liu , D. Pinto , A. C. Walls , F. Zatta , S. K. Zepeda , J. E. Bowen , K. R. Sprouse , A. Joshi , M. Giurdanella , B. Guarino , J. Noack , R. Abdelnabi , S. C. Foo , L. E. Rosen , F. A. Lempp , F. Benigni , G. Snell , J. Neyts , S. P. J. Whelan , H. W. Virgin , J. D. Bloom , D. Corti , M. S. Pizzuto , D. Veesler , Antibody-mediated broad sarbecovirus neutralization through ACE2 molecular mimicry. Science 375, 449–454 (2022). 10.1126/science.abm8143 34990214 PMC9400459

[14] D. R. Martinez , A. Schäfer , S. Gobeil , D. Li , G. De la Cruz , R. Parks , X. Lu , M. Barr , V. Stalls , K. Janowska , E. Beaudoin , K. Manne , K. Mansouri , R. J. Edwards , K. Cronin , B. Yount , K. Anasti , S. A. Montgomery , J. Tang , H. Golding , S. Shen , T. Zhou , P. D. Kwong , B. S. Graham , J. R. Mascola , D. C. Montefiori , S. M. Alam , G. Sempowski , G. D. Sempowski , S. Khurana , K. Wiehe , K. O. Saunders , P. Acharya , B. F. Haynes , R. S. Baric , A broadly cross-reactive antibody neutralizes and protects against sarbecovirus challenge in mice. Sci. Transl. Med. 14, eabj7125 (2022). 10.1126/scitranslmed.abj7125 34726473 PMC8899823

[15] D. Pinto , M. M. Sauer , N. Czudnochowski , J. S. Low , M. A. Tortorici , M. P. Housley , J. Noack , A. C. Walls , J. E. Bowen , B. Guarino , L. E. Rosen , J. di Iulio , J. Jerak , H. Kaiser , S. Islam , S. Jaconi , N. Sprugasci , K. Culap , R. Abdelnabi , C. Foo , L. Coelmont , I. Bartha , S. Bianchi , C. Silacci-Fregni , J. Bassi , R. Marzi , E. Vetti , A. Cassotta , A. Ceschi , P. Ferrari , P. E. Cippà , O. Giannini , S. Ceruti , C. Garzoni , A. Riva , F. Benigni , E. Cameroni , L. Piccoli , M. S. Pizzuto , M. Smithey , D. Hong , A. Telenti , F. A. Lempp , J. Neyts , C. Havenar-Daughton , A. Lanzavecchia , F. Sallusto , G. Snell , H. W. Virgin , M. Beltramello , D. Corti , D. Veesler , Broad betacoronavirus neutralization by a stem helix-specific human antibody. Science 373, 1109–1116 (2021). 10.1126/science.abj3321 34344823 PMC9268357

[16] C. Wang , R. van Haperen , J. Gutiérrez-Álvarez , W. Li , N. M. A. Okba , I. Albulescu , I. Widjaja , B. van Dieren , R. Fernandez-Delgado , I. Sola , D. L. Hurdiss , O. Daramola , F. Grosveld , F. J. M. van Kuppeveld , B. L. Haagmans , L. Enjuanes , D. Drabek , B. J. Bosch , A conserved immunogenic and vulnerable site on the coronavirus spike protein delineated by cross-reactive monoclonal antibodies. Nat. Commun. 12, 1715 (2021). 10.1038/s41467-021-21968-w 33731724 PMC7969777

[17] G. Song , W. He , S. Callaghan , F. Anzanello , D. Huang , J. Ricketts , J. L. Torres , N. Beutler , L. Peng , S. Vargas , J. Cassell , M. Parren , L. Yang , C. Ignacio , D. M. Smith , J. E. Voss , D. Nemazee , A. B. Ward , T. Rogers , D. R. Burton , R. Andrabi , Cross-reactive serum and memory B-cell responses to spike protein in SARS-CoV-2 and endemic coronavirus infection. Nat. Commun. 12, 2938 (2021). 10.1038/s41467-021-23074-3 34011939 PMC8134462

[18] M. M. Sauer , M. A. Tortorici , Y.-J. Park , A. C. Walls , L. Homad , O. J. Acton , J. E. Bowen , C. Wang , X. Xiong , W. de van der Schueren , J. Quispe , B. G. Hoffstrom , B.-J. Bosch , A. T. McGuire , D. Veesler , Structural basis for broad coronavirus neutralization. Nat. Struct. Mol. Biol. 28, 478–486 (2021). 10.1038/s41594-021-00596-4 33981021

[19] P. Zhou , M. Yuan , G. Song , N. Beutler , N. Shaabani , D. Huang , W. T. He , X. Zhu , S. Callaghan , P. Yong , F. Anzanello , L. Peng , J. Ricketts , M. Parren , E. Garcia , S. A. Rawlings , D. M. Smith , D. Nemazee , J. R. Teijaro , T. F. Rogers , I. A. Wilson , D. R. Burton , R. Andrabi , A human antibody reveals a conserved site on beta-coronavirus spike proteins and confers protection against SARS-CoV-2 infection. Sci. Transl. Med. 14, eabi9215 (2022). 10.1126/scitranslmed.abi9215 35133175 PMC8939767

[20] P. Zhou, G. Song, W.-T. He, N. Beutler, L. v Tse, D. R. Martinez, A. Schäfer, F. Anzanello, P. Yong, L. Peng, K. Dueker, R. Musharrafieh, S. Callaghan, T. Capozzola, M. Yuan, H. Liu, O. Limbo, M. Parren, E. Garcia, S. A. Rawlings, D. M. Smith, D. Nemazee, J. G. Jardine, I. A. Wilson, Y. Safonova, T. F. Rogers, R. S. Baric, L. E. Gralinski, D. R. Burton, R. Andrabi, Broadly neutralizing anti-S2 antibodies protect against all three human betacoronaviruses that cause severe disease. bioRxiv 479488 [Preprint] (2022); . 10.1101/2022.03.04.479488

[21] D. Corti , J. Voss , S. J. Gamblin , G. Codoni , A. Macagno , D. Jarrossay , S. G. Vachieri , D. Pinna , A. Minola , F. Vanzetta , C. Silacci , B. M. Fernandez-Rodriguez , G. Agatic , S. Bianchi , I. Giacchetto-Sasselli , L. Calder , F. Sallusto , P. Collins , L. F. Haire , N. Temperton , J. P. M. Langedijk , J. J. Skehel , A. Lanzavecchia , A neutralizing antibody selected from plasma cells that binds to group 1 and group 2 influenza A hemagglutinins. Science 333, 850–856 (2011). 10.1126/science.1205669 21798894

[22] M. M. Sajadi , A. Dashti , Z. Rikhtegaran Tehrani , W. D. Tolbert , M. S. Seaman , X. Ouyang , N. Gohain , M. Pazgier , D. Kim , G. Cavet , J. Yared , R. R. Redfield , G. K. Lewis , A. L. DeVico , Identification of near-pan-neutralizing antibodies against HIV-1 by deconvolution of plasma humoral responses. Cell 173, 1783–1795.e14 (2018). 10.1016/j.cell.2018.03.061 29731169 PMC6003858

[23] H.-X. Liao , R. Lynch , T. Zhou , F. Gao , S. M. Alam , S. D. Boyd , A. Z. Fire , K. M. Roskin , C. A. Schramm , Z. Zhang , J. Zhu , L. Shapiro , J. C. Mullikin , S. Gnanakaran , P. Hraber , K. Wiehe , G. Kelsoe , G. Yang , S.-M. Xia , D. C. Montefiori , R. Parks , K. E. Lloyd , R. M. Scearce , K. A. Soderberg , M. Cohen , G. Kamanga , M. K. Louder , L. M. Tran , Y. Chen , F. Cai , S. Chen , S. Moquin , X. Du , M. G. Joyce , S. Srivatsan , B. Zhang , A. Zheng , G. M. Shaw , B. H. Hahn , T. B. Kepler , B. T. M. Korber , P. D. Kwong , J. R. Mascola , B. F. Haynes , Co-evolution of a broadly neutralizing HIV-1 antibody and founder virus. Nature 496, 469–476 (2013). 10.1038/nature12053 23552890 PMC3637846

[24] J. F. Scheid , H. Mouquet , B. Ueberheide , R. Diskin , F. Klein , T. Y. K. Oliveira , J. Pietzsch , D. Fenyo , A. Abadir , K. Velinzon , A. Hurley , S. Myung , F. Boulad , P. Poignard , D. R. Burton , F. Pereyra , D. D. Ho , B. D. Walker , M. S. Seaman , P. J. Bjorkman , B. T. Chait , M. C. Nussenzweig , Sequence and structural convergence of broad and potent HIV antibodies that mimic CD4 binding. Science 333, 1633–1637 (2011). 10.1126/science.1207227 21764753 PMC3351836

[25] J. Huang , G. Ofek , L. Laub , M. K. Louder , N. A. Doria-Rose , N. S. Longo , H. Imamichi , R. T. Bailer , B. Chakrabarti , S. K. Sharma , S. M. Alam , T. Wang , Y. Yang , B. Zhang , S. A. Migueles , R. Wyatt , B. F. Haynes , P. D. Kwong , J. R. Mascola , M. Connors , Broad and potent neutralization of HIV-1 by a gp41-specific human antibody. Nature 491, 406–412 (2012). 10.1038/nature11544 23151583 PMC4854285

[26] C. Dreyfus , N. S. Laursen , T. Kwaks , D. Zuijdgeest , R. Khayat , D. C. Ekiert , J. H. Lee , Z. Metlagel , M. V. Bujny , M. Jongeneelen , R. van der Vlugt , M. Lamrani , H. J. W. M. Korse , E. Geelen , Ö. Sahin , M. Sieuwerts , J. P. J. Brakenhoff , R. Vogels , O. T. W. Li , L. L. M. Poon , M. Peiris , W. Koudstaal , A. B. Ward , I. A. Wilson , J. Goudsmit , R. H. E. Friesen , Highly conserved protective epitopes on influenza B viruses. Science 337, 1343–1348 (2012). 10.1126/science.1222908 22878502 PMC3538841

[27] D. Sok , D. R. Burton , Recent progress in broadly neutralizing antibodies to HIV. Nat. Immunol. 19, 1179–1188 (2018). 10.1038/s41590-018-0235-7 30333615 PMC6440471

[28] D. Corti , A. Lanzavecchia , Broadly neutralizing antiviral antibodies. Annu. Rev. Immunol. 31, 705–742 (2013). 10.1146/annurev-immunol-032712-095916 23330954

[29] D. Pinna , D. Corti , D. Jarrossay , F. Sallusto , A. Lanzavecchia , Clonal dissection of the human memory B-cell repertoire following infection and vaccination. Eur. J. Immunol. 39, 1260–1270 (2009). 10.1002/eji.200839129 19404981 PMC3864550

[30] A. C. Walls , K. R. Sprouse , J. E. Bowen , A. Joshi , N. Franko , M. J. Navarro , C. Stewart , E. Cameroni , M. McCallum , E. A. Goecker , E. J. Degli-Angeli , J. Logue , A. Greninger , D. Corti , H. Y. Chu , D. Veesler , SARS-CoV-2 breakthrough infections elicit potent, broad, and durable neutralizing antibody responses. Cell 185, 872–880.e3 (2022). 10.1016/j.cell.2022.01.011 35123650 PMC8769922

[31] E. M. Anderson , E. C. Goodwin , A. Verma , C. P. Arevalo , M. J. Bolton , M. E. Weirick , S. Gouma , C. M. McAllister , S. R. Christensen , J. Weaver , P. Hicks , T. B. Manzoni , O. Oniyide , H. Ramage , D. Mathew , A. E. Baxter , D. A. Oldridge , A. R. Greenplate , J. E. Wu , C. Alanio , K. D’Andrea , O. Kuthuru , J. Dougherty , A. Pattekar , J. Kim , N. Han , S. A. Apostolidis , A. C. Huang , L. A. Vella , L. Kuri-Cervantes , M. B. Pampena , M. R. Betts , E. J. Wherry , N. J. Meyer , S. Cherry , P. Bates , D. J. Rader , S. E. Hensley ; UPenn COVID Processing Unit , Seasonal human coronavirus antibodies are boosted upon SARS-CoV-2 infection but not associated with protection. Cell 184, 1858–1864.e10 (2021). 10.1016/j.cell.2021.02.010 33631096 PMC7871851

[32] M. Hoffmann , H. Kleine-Weber , S. Schroeder , N. Krüger , T. Herrler , S. Erichsen , T. S. Schiergens , G. Herrler , N. H. Wu , A. Nitsche , M. A. Müller , C. Drosten , S. Pöhlmann , SARS-CoV-2 cell entry depends on ACE2 and TMPRSS2 and is blocked by a clinically proven protease inhibitor. Cell 181, 271–280.e8 (2020). 10.1016/j.cell.2020.02.052 32142651 PMC7102627

[33] C.-L. Hsieh , J. A. Goldsmith , J. M. Schaub , A. M. DiVenere , H.-C. Kuo , K. Javanmardi , K. C. Le , D. Wrapp , A. G. Lee , Y. Liu , C.-W. Chou , P. O. Byrne , C. K. Hjorth , N. V. Johnson , J. Ludes-Meyers , A. W. Nguyen , J. Park , N. Wang , D. Amengor , J. J. Lavinder , G. C. Ippolito , J. A. Maynard , I. J. Finkelstein , J. S. McLellan , Structure-based design of prefusion-stabilized SARS-CoV-2 spikes. Science 369, 1501–1505 (2020). 10.1126/science.abd0826 32703906 PMC7402631

[34] E. Shrock , E. Fujimura , T. Kula , R. T. Timms , I.-H. Lee , Y. Leng , M. L. Robinson , B. M. Sie , M. Z. Li , Y. Chen , J. Logue , A. Zuiani , D. McCulloch , F. J. N. Lelis , S. Henson , D. R. Monaco , M. Travers , S. Habibi , W. A. Clarke , P. Caturegli , O. Laeyendecker , A. Piechocka-Trocha , J. Z. Li , A. Khatri , H. Y. Chu , A.-C. Villani , K. Kays , M. B. Goldberg , N. Hacohen , M. R. Filbin , X. G. Yu , B. D. Walker , D. R. Wesemann , H. B. Larman , J. A. Lederer , S. J. Elledge , Viral epitope profiling of COVID-19 patients reveals cross-reactivity and correlates of severity. Science 370, eabd4250 (2020). 10.1126/science.abd4250 32994364 PMC7857405

[35] K. W. Ng , N. Faulkner , G. H. Cornish , A. Rosa , R. Harvey , S. Hussain , R. Ulferts , C. Earl , A. G. Wrobel , D. J. Benton , C. Roustan , W. Bolland , R. Thompson , A. Agua-Doce , P. Hobson , J. Heaney , H. Rickman , S. Paraskevopoulou , C. F. Houlihan , K. Thomson , E. Sanchez , G. Y. Shin , M. J. Spyer , D. Joshi , N. O’Reilly , P. A. Walker , S. Kjaer , A. Riddell , C. Moore , B. R. Jebson , M. Wilkinson , L. R. Marshall , E. C. Rosser , A. Radziszewska , H. Peckham , C. Ciurtin , L. R. Wedderburn , R. Beale , C. Swanton , S. Gandhi , B. Stockinger , J. McCauley , S. J. Gamblin , L. E. McCoy , P. Cherepanov , E. Nastouli , G. Kassiotis , Preexisting and de novo humoral immunity to SARS-CoV-2 in humans. Science 370, 1339–1343 (2020). 10.1126/science.abe1107 33159009 PMC7857411

[36] C. M. Poh , G. Carissimo , B. Wang , S. N. Amrun , C. Y.-P. Lee , R. S.-L. Chee , S.-W. Fong , N. K.-W. Yeo , W.-H. Lee , A. Torres-Ruesta , Y.-S. Leo , M. I.-C. Chen , S.-Y. Tan , L. Y. A. Chai , S. Kalimuddin , S. S. G. Kheng , S.-Y. Thien , B. E. Young , D. C. Lye , B. J. Hanson , C.-I. Wang , L. Renia , L. F. P. Ng , Two linear epitopes on the SARS-CoV-2 spike protein that elicit neutralising antibodies in COVID-19 patients. Nat. Commun. 11, 2806 (2020). 10.1038/s41467-020-16638-2 32483236 PMC7264175

[37] C. Daniel , R. Anderson , M. J. Buchmeier , J. O. Fleming , W. J. Spaan , H. Wege , P. J. Talbot , Identification of an immunodominant linear neutralization domain on the S2 portion of the murine coronavirus spike glycoprotein and evidence that it forms part of complex tridimensional structure. J. Virol. 67, 1185–1194 (1993). 10.1128/jvi.67.3.1185-1194.1993 7679743 PMC237483

[38] H. Zhang , G. Wang , J. Li , Y. Nie , X. Shi , G. Lian , W. Wang , X. Yin , Y. Zhao , X. Qu , M. Ding , H. Deng , Identification of an antigenic determinant on the S2 domain of the severe acute respiratory syndrome coronavirus spike glycoprotein capable of inducing neutralizing antibodies. J. Virol. 78, 6938–6945 (2004). 10.1128/JVI.78.13.6938-6945.2004 15194770 PMC421668

[39] A. C. Walls , M. A. Tortorici , B. J. Bosch , B. Frenz , P. J. M. Rottier , F. DiMaio , F. A. Rey , D. Veesler , Cryo-electron microscopy structure of a coronavirus spike glycoprotein trimer. Nature 531, 114–117 (2016). 10.1038/nature16988 26855426 PMC5018210

[40] Q. Xiong, L. Cao, C. Ma, C. Liu, J. Si, P. Liu, M. Gu, C. Wang, L. Shi, F. Tong, M. Huang, J. Li, C. Zhao, C. Shen, Y. Chen, H. Zhao, K. Lan, X. Wang, H. Yan, Close relatives of MERS-CoV in bats use ACE2 as their functional receptors. bioRxiv 477490 [Preprint] (2022); . 10.1101/2022.01.24.477490 PMC9734910 36477529

[41] F. A. Lempp , L. B. Soriaga , M. Montiel-Ruiz , F. Benigni , J. Noack , Y.-J. Park , S. Bianchi , A. C. Walls , J. E. Bowen , J. Zhou , H. Kaiser , A. Joshi , M. Agostini , M. Meury , E. Dellota Jr ., S. Jaconi , E. Cameroni , J. Martinez-Picado , J. Vergara-Alert , N. Izquierdo-Useros , H. W. Virgin , A. Lanzavecchia , D. Veesler , L. A. Purcell , A. Telenti , D. Corti , Lectins enhance SARS-CoV-2 infection and influence neutralizing antibodies. Nature 598, 342–347 (2021). 10.1038/s41586-021-03925-1 34464958

[42] H. V. Dang , Y.-P. Chan , Y.-J. Park , J. Snijder , S. C. Da Silva , B. Vu , L. Yan , Y.-R. Feng , B. Rockx , T. W. Geisbert , C. E. Mire , C. C. Broder , D. Veesler , An antibody against the F glycoprotein inhibits Nipah and Hendra virus infections. Nat. Struct. Mol. Biol. 26, 980–987 (2019). 10.1038/s41594-019-0308-9 31570878 PMC6858553

[43] A. C. Walls , M. A. Tortorici , J. Snijder , X. Xiong , B.-J. Bosch , F. A. Rey , D. Veesler , Tectonic conformational changes of a coronavirus spike glycoprotein promote membrane fusion. Proc. Natl. Acad. Sci. U.S.A. 114, 11157–11162 (2017). 10.1073/pnas.1708727114 29073020 PMC5651768

[44] B. A. Johnson , X. Xie , A. L. Bailey , B. Kalveram , K. G. Lokugamage , A. Muruato , J. Zou , X. Zhang , T. Juelich , J. K. Smith , L. Zhang , N. Bopp , C. Schindewolf , M. Vu , A. Vanderheiden , E. S. Winkler , D. Swetnam , J. A. Plante , P. Aguilar , K. S. Plante , V. Popov , B. Lee , S. C. Weaver , M. S. Suthar , A. L. Routh , P. Ren , Z. Ku , Z. An , K. Debbink , M. S. Diamond , P.-Y. Shi , A. N. Freiberg , V. D. Menachery , Loss of furin cleavage site attenuates SARS-CoV-2 pathogenesis. Nature 591, 293–299 (2021). 10.1038/s41586-021-03237-4 33494095 PMC8175039

[45] M. Hoffmann , H. Kleine-Weber , S. Pöhlmann , A multibasic cleavage site in the spike protein of SARS-CoV-2 is essential for infection of human lung cells. Mol. Cell 78, 779–784.e5 (2020). 10.1016/j.molcel.2020.04.022 32362314 PMC7194065

[46] S. M.-C. Gobeil , R. Henderson , V. Stalls , K. Janowska , X. Huang , A. May , M. Speakman , E. Beaudoin , K. Manne , D. Li , R. Parks , M. Barr , M. Deyton , M. Martin , K. Mansouri , R. J. Edwards , A. Eaton , D. C. Montefiori , G. D. Sempowski , K. O. Saunders , K. Wiehe , W. Williams , B. Korber , B. F. Haynes , P. Acharya , Structural diversity of the SARS-CoV-2 Omicron spike. Mol. Cell 82, 2050–2068.e6 (2022). 10.1016/j.molcel.2022.03.028 35447081 PMC8947964

[47] W. Li , M. J. Moore , N. Vasilieva , J. Sui , S. K. Wong , M. A. Berne , M. Somasundaran , J. L. Sullivan , K. Luzuriaga , T. C. Greenough , H. Choe , M. Farzan , Angiotensin-converting enzyme 2 is a functional receptor for the SARS coronavirus. Nature 426, 450–454 (2003). 10.1038/nature02145 14647384 PMC7095016

[48] V. S. Raj , H. Mou , S. L. Smits , D. H. W. Dekkers , M. A. Müller , R. Dijkman , D. Muth , J. A. A. Demmers , A. Zaki , R. A. M. Fouchier , V. Thiel , C. Drosten , P. J. M. Rottier , A. D. M. E. Osterhaus , B. J. Bosch , B. L. Haagmans , Dipeptidyl peptidase 4 is a functional receptor for the emerging human coronavirus-EMC. Nature 495, 251–254 (2013). 10.1038/nature12005 23486063 PMC7095326

[49] H. Hofmann , K. Pyrc , L. van der Hoek , M. Geier , B. Berkhout , S. Pöhlmann , Human coronavirus NL63 employs the severe acute respiratory syndrome coronavirus receptor for cellular entry. Proc. Natl. Acad. Sci. U.S.A. 102, 7988–7993 (2005). 10.1073/pnas.0409465102 15897467 PMC1142358

[50] C. L. Yeager , R. A. Ashmun , R. K. Williams , C. B. Cardellichio , L. H. Shapiro , A. T. Look , K. V. Holmes , Human aminopeptidase N is a receptor for human coronavirus 229E. Nature 357, 420–422 (1992). 10.1038/357420a0 1350662 PMC7095410

[51] M. A. Tortorici , M. Beltramello , F. A. Lempp , D. Pinto , H. V. Dang , L. E. Rosen , M. McCallum , J. Bowen , A. Minola , S. Jaconi , F. Zatta , A. De Marco , B. Guarino , S. Bianchi , E. J. Lauron , H. Tucker , J. Zhou , A. Peter , C. Havenar-Daughton , J. A. Wojcechowskyj , J. B. Case , R. E. Chen , H. Kaiser , M. Montiel-Ruiz , M. Meury , N. Czudnochowski , R. Spreafico , J. Dillen , C. Ng , N. Sprugasci , K. Culap , F. Benigni , R. Abdelnabi , S. C. Foo , M. A. Schmid , E. Cameroni , A. Riva , A. Gabrieli , M. Galli , M. S. Pizzuto , J. Neyts , M. S. Diamond , H. W. Virgin , G. Snell , D. Corti , K. Fink , D. Veesler , Ultrapotent human antibodies protect against SARS-CoV-2 challenge via multiple mechanisms. Science 370, 950–957 (2020). 10.1126/science.abe3354 32972994 PMC7857395

[52] A. C. Walls , X. Xiong , Y. J. Park , M. A. Tortorici , J. Snijder , J. Quispe , E. Cameroni , R. Gopal , M. Dai , A. Lanzavecchia , M. Zambon , F. A. Rey , D. Corti , D. Veesler , Unexpected receptor functional mimicry elucidates activation of coronavirus fusion. Cell 176, 1026–1039.e15 (2019). 10.1016/j.cell.2018.12.028 30712865 PMC6751136

[53] D. R. Burton , Advancing an HIV vaccine; advancing vaccinology. Nat. Rev. Immunol. 19, 77–78 (2019). 10.1038/s41577-018-0103-6 30560910 PMC6425752

[54] M. Bonsignori , H.-X. Liao , F. Gao , W. B. Williams , S. M. Alam , D. C. Montefiori , B. F. Haynes , Antibody-virus co-evolution in HIV infection: Paths for HIV vaccine development. Immunol. Rev. 275, 145–160 (2017). 10.1111/imr.12509 28133802 PMC5302796

[55] J.-E. Park , K. Li , A. Barlan , A. R. Fehr , S. Perlman , P. B. McCray Jr ., T. Gallagher , Proteolytic processing of Middle East respiratory syndrome coronavirus spikes expands virus tropism. Proc. Natl. Acad. Sci. U.S.A. 113, 12262–12267 (2016). 10.1073/pnas.1608147113 27791014 PMC5086990

[56] J. K. Millet , G. R. Whittaker , Host cell entry of Middle East respiratory syndrome coronavirus after two-step, furin-mediated activation of the spike protein. Proc. Natl. Acad. Sci. U.S.A. 111, 15214–15219 (2014). 10.1073/pnas.1407087111 25288733 PMC4210292

[57] P. V. Raghuvamsi , N. K. Tulsian , F. Samsudin , X. Qian , K. Purushotorman , G. Yue , M. M. Kozma , W. Y. Hwa , J. Lescar , P. J. Bond , P. A. MacAry , G. S. Anand , SARS-CoV-2 S protein:ACE2 interaction reveals novel allosteric targets. eLife 10, e63646 (2021). 10.7554/eLife.63646 33554856 PMC7932696

[58] S. Yu , X. Zheng , B. Zhou , J. Li , M. Chen , R. Deng , G. Wong , D. Lavillette , G. Meng , SARS-CoV-2 spike engagement of ACE2 primes S2′ site cleavage and fusion initiation. Proc. Natl. Acad. Sci. U.S.A. 119, e2111199119 (2022). 10.1073/pnas.2111199119 34930824 PMC8740742

[59] E. Qing , P. Li , L. Cooper , S. Schulz , H.-M. Jäck , L. Rong , S. Perlman , T. Gallagher , Inter-domain communication in SARS-CoV-2 spike proteins controls protease-triggered cell entry. Cell Rep. 39, 110786 (2022). 10.1016/j.celrep.2022.110786 35477024 PMC9015963

[60] C. Cheng , K. Xu , R. Kong , G. Y. Chuang , A. R. Corrigan , H. Geng , K. R. Hill , A. J. Jafari , S. O’Dell , L. Ou , R. Rawi , A. P. Rowshan , E. K. Sarfo , M. Sastry , K. O. Saunders , S. D. Schmidt , S. Wang , W. Wu , B. Zhang , N. A. Doria-Rose , B. F. Haynes , D. G. Scorpio , L. Shapiro , J. R. Mascola , P. D. Kwong , Consistent elicitation of cross-clade HIV-neutralizing responses achieved in guinea pigs after fusion peptide priming by repetitive envelope trimer boosting. PLOS ONE 14, e0215163 (2019). 10.1371/journal.pone.0215163 30995238 PMC6469787

[61] K. Xu , P. Acharya , R. Kong , C. Cheng , G. Y. Chuang , K. Liu , M. K. Louder , S. O’Dell , R. Rawi , M. Sastry , C. H. Shen , B. Zhang , T. Zhou , M. Asokan , R. T. Bailer , M. Chambers , X. Chen , C. W. Choi , V. P. Dandey , N. A. Doria-Rose , A. Druz , E. T. Eng , S. K. Farney , K. E. Foulds , H. Geng , I. S. Georgiev , J. Gorman , K. R. Hill , A. J. Jafari , Y. D. Kwon , Y. T. Lai , T. Lemmin , K. McKee , T. Y. Ohr , L. Ou , D. Peng , A. P. Rowshan , Z. Sheng , J. P. Todd , Y. Tsybovsky , E. G. Viox , Y. Wang , H. Wei , Y. Yang , A. F. Zhou , R. Chen , L. Yang , D. G. Scorpio , A. B. McDermott , L. Shapiro , B. Carragher , C. S. Potter , J. R. Mascola , P. D. Kwong , Epitope-based vaccine design yields fusion peptide-directed antibodies that neutralize diverse strains of HIV-1. Nat. Med. 24, 857–867 (2018). 10.1038/s41591-018-0042-6 29867235 PMC6358635

[62] R. Kong , K. Xu , T. Zhou , P. Acharya , T. Lemmin , K. Liu , G. Ozorowski , C. Soto , J. D. Taft , R. T. Bailer , E. M. Cale , L. Chen , C. W. Choi , G.-Y. Chuang , N. A. Doria-Rose , A. Druz , I. S. Georgiev , J. Gorman , J. Huang , M. G. Joyce , M. K. Louder , X. Ma , K. McKee , S. O’Dell , M. Pancera , Y. Yang , S. C. Blanchard , W. Mothes , D. R. Burton , W. C. Koff , M. Connors , A. B. Ward , P. D. Kwong , J. R. Mascola , Fusion peptide of HIV-1 as a site of vulnerability to neutralizing antibody. Science 352, 828–833 (2016). 10.1126/science.aae0474 27174988 PMC4917739

[63] L. Ou , W.-P. Kong , G.-Y. Chuang , M. Ghosh , K. Gulla , S. O’Dell , J. Varriale , N. Barefoot , A. Changela , C. W. Chao , C. Cheng , A. Druz , R. Kong , K. McKee , R. Rawi , E. K. Sarfo , A. Schön , A. Shaddeau , Y. Tsybovsky , R. Verardi , S. Wang , T. G. Wanninger , K. Xu , G. J. Yang , B. Zhang , Y. Zhang , T. Zhou , F. J. Arnold , N. A. Doria-Rose , Q. P. Lei , E. T. Ryan , W. F. Vann , J. R. Mascola , P. D. Kwong ; VRC Production Program , Preclinical development of a fusion peptide conjugate as an HIV vaccine immunogen. Sci. Rep. 10, 3032 (2020). 10.1038/s41598-020-59711-y 32080235 PMC7033230

[64] R. Nachbagauer , J. Feser , A. Naficy , D. I. Bernstein , J. Guptill , E. B. Walter , F. Berlanda-Scorza , D. Stadlbauer , P. C. Wilson , T. Aydillo , M. A. Behzadi , D. Bhavsar , C. Bliss , C. Capuano , J. M. Carreño , V. Chromikova , C. Claeys , L. Coughlan , A. W. Freyn , C. Gast , A. Javier , K. Jiang , C. Mariottini , M. McMahon , M. McNeal , A. Solórzano , S. Strohmeier , W. Sun , M. Van der Wielen , B. L. Innis , A. García-Sastre , P. Palese , F. Krammer , A chimeric hemagglutinin-based universal influenza virus vaccine approach induces broad and long-lasting immunity in a randomized, placebo-controlled phase I trial. Nat. Med. 27, 106–114 (2021). 10.1038/s41591-020-1118-7 33288923

[65] H. M. Yassine , J. C. Boyington , P. M. McTamney , C. J. Wei , M. Kanekiyo , W. P. Kong , J. R. Gallagher , L. Wang , Y. Zhang , M. G. Joyce , D. Lingwood , S. M. Moin , H. Andersen , Y. Okuno , S. S. Rao , A. K. Harris , P. D. Kwong , J. R. Mascola , G. J. Nabel , B. S. Graham , Hemagglutinin-stem nanoparticles generate heterosubtypic influenza protection. Nat. Med. 21, 1065–1070 (2015). 10.1038/nm.3927 26301691

[66] A. Impagliazzo , F. Milder , H. Kuipers , M. V. Wagner , X. Zhu , R. M. B. Hoffman , R. van Meersbergen , J. Huizingh , P. Wanningen , J. Verspuij , M. de Man , Z. Ding , A. Apetri , B. Kükrer , E. Sneekes-Vriese , D. Tomkiewicz , N. S. Laursen , P. S. Lee , A. Zakrzewska , L. Dekking , J. Tolboom , L. Tettero , S. van Meerten , W. Yu , W. Koudstaal , J. Goudsmit , A. B. Ward , W. Meijberg , I. A. Wilson , K. Radošević , A stable trimeric influenza hemagglutinin stem as a broadly protective immunogen. Science 349, 1301–1306 (2015). 10.1126/science.aac7263 26303961

[67] S. Belouzard , V. C. Chu , G. R. Whittaker , Activation of the SARS coronavirus spike protein via sequential proteolytic cleavage at two distinct sites. Proc. Natl. Acad. Sci. U.S.A. 106, 5871–5876 (2009). 10.1073/pnas.0809524106 19321428 PMC2660061

[68] I. G. Madu , S. L. Roth , S. Belouzard , G. R. Whittaker , Characterization of a highly conserved domain within the severe acute respiratory syndrome coronavirus spike protein S2 domain with characteristics of a viral fusion peptide. J. Virol. 83, 7411–7421 (2009). 10.1128/JVI.00079-09 19439480 PMC2708636

[69] J. S. Low , D. Vaqueirinho , F. Mele , M. Foglierini , J. Jerak , M. Perotti , D. Jarrossay , S. Jovic , L. Perez , R. Cacciatore , T. Terrot , A. F. Pellanda , M. Biggiogero , C. Garzoni , P. Ferrari , A. Ceschi , A. Lanzavecchia , F. Sallusto , A. Cassotta , Clonal analysis of immunodominance and cross-reactivity of the CD4 T cell response to SARS-CoV-2. Science 372, 1336–1341 (2021). 10.1126/science.abg8985 34006597 PMC8168615

[70] M. N. Prichard , C. Shipman Jr ., A three-dimensional model to analyze drug-drug interactions. Antiviral Res. 14, 181–205 (1990). 10.1016/0166-3542(90)90001-N 2088205

[71] T. Tiller , E. Meffre , S. Yurasov , M. Tsuiji , M. C. Nussenzweig , H. Wardemann , Efficient generation of monoclonal antibodies from single human B cells by single cell RT-PCR and expression vector cloning. J. Immunol. Methods 329, 112–124 (2008). 10.1016/j.jim.2007.09.017 17996249 PMC2243222

[72] J. E. Bowen, A. C. Walls, A. Joshi, K. R. Sprouse, C. Stewart, M. A. Tortorici, N. M. Franko, J. K. Logue, I. G. Mazzitelli, S. W. Tiles, K. Ahmed, A. Shariq, G. Snell, N. T. Iqbal, J. Geffner, A. Bandera, A. Gori, R. Grifantini, H. Y. Chu, W. C. van Voorhis, D. Corti, D. Veesler, SARS-CoV-2 spike conformation determines plasma neutralizing activity. bioRxiv 473391 [Preprint] (2021); . 10.1101/2021.12.19.473391

[73] E. Olmedillas, C. J. Mann, W. Peng, Y.-T. Wang, R. D. Avalos, D. Bedinger, K. Valentine, N. Shafee, S. L. Schendel, M. Yuan, G. Lang, R. Rouet, D. Christ, W. Jiang, I. A. Wilson, T. Germann, S. Shresta, J. Snijder, E. O. Saphire, Structure-based design of a highly stable, covalently-linked SARS-CoV-2 spike trimer with improved structural properties and immunogenicity. bioRxiv 441046 [Preprint] (2021); . 10.1101/2021.05.06.441046

[74] J. Pallesen , N. Wang , K. S. Corbett , D. Wrapp , R. N. Kirchdoerfer , H. L. Turner , C. A. Cottrell , M. M. Becker , L. Wang , W. Shi , W.-P. Kong , E. L. Andres , A. N. Kettenbach , M. R. Denison , J. D. Chappell , B. S. Graham , A. B. Ward , J. S. McLellan , Immunogenicity and structures of a rationally designed prefusion MERS-CoV spike antigen. Proc. Natl. Acad. Sci. U.S.A. 114, E7348–E7357 (2017). 10.1073/pnas.1707304114 28807998 PMC5584442

[75] H. X. Liao , M. C. Levesque , A. Nagel , A. Dixon , R. Zhang , E. Walter , R. Parks , J. Whitesides , D. J. Marshall , K. K. Hwang , Y. Yang , X. Chen , F. Gao , S. Munshaw , T. B. Kepler , T. Denny , M. A. Moody , B. F. Haynes , High-throughput isolation of immunoglobulin genes from single human B cells and expression as monoclonal antibodies. J. Virol. Methods 158, 171–179 (2009). 10.1016/j.jviromet.2009.02.014 19428587 PMC2805188

[76] J. Ye , N. Ma , T. L. Madden , J. M. Ostell , IgBLAST: An immunoglobulin variable domain sequence analysis tool. Nucleic Acids Res. 41, W34–W40 (2013). 10.1093/nar/gkt382 23671333 PMC3692102

[77] M.-P. Lefranc, G. Lefranc, *The Immunoglobulin FactsBook* (Academic, 2001).

[78] K. B. Hoehn , J. A. Vander Heiden , J. Q. Zhou , G. Lunter , O. G. Pybus , S. H. Kleinstein , Repertoire-wide phylogenetic models of B cell molecular evolution reveal evolutionary signatures of aging and vaccination. Proc. Natl. Acad. Sci. U.S.A. 116, 22664–22672 (2019). 10.1073/pnas.1906020116 31636219 PMC6842591

[79] C. Suloway , J. Pulokas , D. Fellmann , A. Cheng , F. Guerra , J. Quispe , S. Stagg , C. S. Potter , B. Carragher , Automated molecular microscopy: The new Leginon system. J. Struct. Biol. 151, 41–60 (2005). 10.1016/j.jsb.2005.03.010 15890530

[80] K. H. D. Crawford , R. Eguia , A. S. Dingens , A. N. Loes , K. D. Malone , C. R. Wolf , H. Y. Chu , M. A. Tortorici , D. Veesler , M. Murphy , D. Pettie , N. P. King , A. B. Balazs , J. D. Bloom , Protocol and reagents for pseudotyping lentiviral particles with SARS-CoV-2 spike protein for neutralization assays. Viruses 12, 513 (2020). 10.3390/v12050513 32384820 PMC7291041

[81] Y. Kaname , H. Tani , C. Kataoka , M. Shiokawa , S. Taguwa , T. Abe , K. Moriishi , T. Kinoshita , Y. Matsuura , Acquisition of complement resistance through incorporation of CD55/decay-accelerating factor into viral particles bearing baculovirus GP64. J. Virol. 84, 3210–3219 (2010). 10.1128/JVI.02519-09 20071581 PMC2838113

[82] W. Kabsch , XDS. Acta Crystallogr. D Biol. Crystallogr. 66, 125–132 (2010). 10.1107/S0907444909047337 20124692 PMC2815665

[83] T. G. G. Battye , L. Kontogiannis , O. Johnson , H. R. Powell , A. G. W. Leslie , iMOSFLM: A new graphical interface for diffraction-image processing with MOSFLM. Acta Crystallogr. D Biol. Crystallogr. 67, 271–281 (2011). 10.1107/S0907444910048675 21460445 PMC3069742

[84] P. R. Evans , G. N. Murshudov , How good are my data and what is the resolution? Acta Crystallogr. D Biol. Crystallogr. 69, 1204–1214 (2013). 10.1107/S0907444913000061 23793146 PMC3689523

[85] A. J. McCoy , R. W. Grosse-Kunstleve , P. D. Adams , M. D. Winn , L. C. Storoni , R. J. Read , Phaser crystallographic software. J. Appl. Crystallogr. 40, 658–674 (2007). 10.1107/S0021889807021206 19461840 PMC2483472

[86] P. Emsley , B. Lohkamp , W. G. Scott , K. Cowtan , Features and development of Coot. Acta Crystallogr. D Biol. Crystallogr. 66, 486–501 (2010). 10.1107/S0907444910007493 20383002 PMC2852313

[87] D. Liebschner , P. V. Afonine , M. L. Baker , G. Bunkóczi , V. B. Chen , T. I. Croll , B. Hintze , L. W. Hung , S. Jain , A. J. McCoy , N. W. Moriarty , R. D. Oeffner , B. K. Poon , M. G. Prisant , R. J. Read , J. S. Richardson , D. C. Richardson , M. D. Sammito , O. V. Sobolev , D. H. Stockwell , T. C. Terwilliger , A. G. Urzhumtsev , L. L. Videau , C. J. Williams , P. D. Adams , Macromolecular structure determination using X-rays, neutrons and electrons: Recent developments in Phenix. Acta Crystallogr. D Struct. Biol. 75, 861–877 (2019). 10.1107/S2059798319011471 31588918 PMC6778852

[88] E. Blanc , P. Roversi , C. Vonrhein , C. Flensburg , S. M. Lea , G. Bricogne , Refinement of severely incomplete structures with maximum likelihood in BUSTER-TNT. Acta Crystallogr. D Biol. Crystallogr. 60, 2210–2221 (2004). 10.1107/S0907444904016427 15572774

[89] T. Giroglou , J. Cinatl Jr ., H. Rabenau , C. Drosten , H. Schwalbe , H. W. Doerr , D. von Laer , Retroviral vectors pseudotyped with severe acute respiratory syndrome coronavirus S protein. J. Virol. 78, 9007–9015 (2004). 10.1128/JVI.78.17.9007-9015.2004 15308697 PMC506966

[90] R. Boudewijns , H. J. Thibaut , S. J. F. Kaptein , R. Li , V. Vergote , L. Seldeslachts , J. Van Weyenbergh , C. De Keyzer , L. Bervoets , S. Sharma , L. Liesenborghs , J. Ma , S. Jansen , D. Van Looveren , T. Vercruysse , X. Wang , D. Jochmans , E. Martens , K. Roose , D. De Vlieger , B. Schepens , T. Van Buyten , S. Jacobs , Y. Liu , J. Martí-Carreras , B. Vanmechelen , T. Wawina-Bokalanga , L. Delang , J. Rocha-Pereira , L. Coelmont , W. Chiu , P. Leyssen , E. Heylen , D. Schols , L. Wang , L. Close , J. Matthijnssens , M. Van Ranst , V. Compernolle , G. Schramm , K. Van Laere , X. Saelens , N. Callewaert , G. Opdenakker , P. Maes , B. Weynand , C. Cawthorne , G. Vande Velde , Z. Wang , J. Neyts , K. Dallmeier , STAT2 signaling restricts viral dissemination but drives severe pneumonia in SARS-CoV-2 infected hamsters. Nat. Commun. 11, 5838 (2020). 10.1038/s41467-020-19684-y 33203860 PMC7672082

[91] L. Sanchez-Felipe , T. Vercruysse , S. Sharma , J. Ma , V. Lemmens , D. Van Looveren , M. P. Arkalagud Javarappa , R. Boudewijns , B. Malengier-Devlies , L. Liesenborghs , S. J. F. Kaptein , C. De Keyzer , L. Bervoets , S. Debaveye , M. Rasulova , L. Seldeslachts , L.-H. Li , S. Jansen , M. B. Yakass , B. E. Verstrepen , K. P. Böszörményi , G. Kiemenyi-Kayere , N. van Driel , O. Quaye , X. Zhang , S. Ter Horst , N. Mishra , W. Deboutte , J. Matthijnssens , L. Coelmont , C. Vandermeulen , E. Heylen , V. Vergote , D. Schols , Z. Wang , W. Bogers , T. Kuiken , E. Verschoor , C. Cawthorne , K. Van Laere , G. Opdenakker , G. Vande Velde , B. Weynand , D. E. Teuwen , P. Matthys , J. Neyts , H. Jan Thibaut , K. Dallmeier , A single-dose live-attenuated YF17D-vectored SARS-CoV-2 vaccine candidate. Nature 590, 320–325 (2021). 10.1038/s41586-020-3035-9 33260195

[92] S. J. F. Kaptein , S. Jacobs , L. Langendries , L. Seldeslachts , S. Ter Horst , L. Liesenborghs , B. Hens , V. Vergote , E. Heylen , K. Barthelemy , E. Maas , C. De Keyzer , L. Bervoets , J. Rymenants , T. Van Buyten , X. Zhang , R. Abdelnabi , J. Pang , R. Williams , H. J. Thibaut , K. Dallmeier , R. Boudewijns , J. Wouters , P. Augustijns , N. Verougstraete , C. Cawthorne , J. Breuer , C. Solas , B. Weynand , P. Annaert , I. Spriet , G. Vande Velde , J. Neyts , J. Rocha-Pereira , L. Delang , Favipiravir at high doses has potent antiviral activity in SARS-CoV-2-infected hamsters, whereas hydroxychloroquine lacks activity. Proc. Natl. Acad. Sci. U.S.A. 117, 26955–26965 (2020). 10.1073/pnas.2014441117 33037151 PMC7604414

[93] R. Abdelnabi , C. S. Foo , D. Jochmans , L. Vangeel , S. De Jonghe , P. Augustijns , R. Mols , B. Weynand , T. Wattanakul , R. M. Hoglund , J. Tarning , C. E. Mowbray , P. Sjö , F. Escudié , I. Scandale , E. Chatelain , J. Neyts , The oral protease inhibitor (PF-07321332) protects Syrian hamsters against infection with SARS-CoV-2 variants of concern. Nat. Commun. 13, 719 (2022). 10.1038/s41467-022-28354-0 35169114 PMC8847371

[94] L. J. Reed , H. Muench , A simple method of estimating fifty per cent endpoints. Am. J. Epidemiol. 27, 493–497 (1938). 10.1093/oxfordjournals.aje.a118408

[95] J. Tan , B. K. Sack , D. Oyen , I. Zenklusen , L. Piccoli , S. Barbieri , M. Foglierini , C. S. Fregni , J. Marcandalli , S. Jongo , S. Abdulla , L. Perez , G. Corradin , L. Varani , F. Sallusto , B. K. L. Sim , S. L. Hoffman , S. H. I. Kappe , C. Daubenberger , I. A. Wilson , A. Lanzavecchia , A public antibody lineage that potently inhibits malaria infection through dual binding to the circumsporozoite protein. Nat. Med. 24, 401–407 (2018). 10.1038/nm.4513 29554084 PMC5893353

[96] R. Abdelnabi , R. Boudewijns , C. S. Foo , L. Seldeslachts , L. Sanchez-Felipe , X. Zhang , L. Delang , P. Maes , S. J. F. Kaptein , B. Weynand , G. Vande Velde , J. Neyts , K. Dallmeier , Comparing infectivity and virulence of emerging SARS-CoV-2 variants in Syrian hamsters. EBioMedicine 68, 103403 (2021). 10.1016/j.ebiom.2021.103403 34049240 PMC8143995

[97] E. F. Pettersen , T. D. Goddard , C. C. Huang , G. S. Couch , D. M. Greenblatt , E. C. Meng , T. E. Ferrin , UCSF Chimera—A visualization system for exploratory research and analysis. J. Comput. Chem. 25, 1605–1612 (2004). 10.1002/jcc.20084 15264254

[98] V. B. Chen , W. B. Arendall 3rd , J. J. Headd , D. A. Keedy , R. M. Immormino , G. J. Kapral , L. W. Murray , J. S. Richardson , D. C. Richardson , MolProbity: All-atom structure validation for macromolecular crystallography. Acta Crystallogr. D Biol. Crystallogr. 66, 12–21 (2010). 10.1107/S0907444909042073 20057044 PMC2803126

[99] T. D. Goddard , C. C. Huang , E. C. Meng , E. F. Pettersen , G. S. Couch , J. H. Morris , T. E. Ferrin , UCSF ChimeraX: Meeting modern challenges in visualization and analysis. Protein Sci. 27, 14–25 (2018). 10.1002/pro.3235 28710774 PMC5734306

[100] D. Pinto , E. Montani , M. Bolli , G. Garavaglia , F. Sallusto , A. Lanzavecchia , D. Jarrossay , A functional BCR in human IgA and IgM plasma cells. Blood 121, 4110–4114 (2013). 10.1182/blood-2012-09-459289 23550036

[101] X. Y. Ge , J. L. Li , X.-L. Yang , A. A. Chmura , G. Zhu , J. H. Epstein , J. K. Mazet , B. Hu , W. Zhang , C. Peng , Y. J. Zhang , C. M. Luo , B. Tan , N. Wang , Y. Zhu , G. Crameri , S. Y. Zhang , L. F. Wang , P. Daszak , Z. L. Shi , Isolation and characterization of a bat SARS-like coronavirus that uses the ACE2 receptor. Nature 503, 535–538 (2013). 10.1038/nature12711 24172901 PMC5389864

[102] R. Vlasak , W. Luytjes , W. Spaan , P. Palese , Human and bovine coronaviruses recognize sialic acid-containing receptors similar to those of influenza C viruses. Proc. Natl. Acad. Sci. U.S.A. 85, 4526–4529 (1988). 10.1073/pnas.85.12.4526 3380803 PMC280463

[103] X. Huang , W. Dong , A. Milewska , A. Golda , Y. Qi , Q. K. Zhu , W. A. Marasco , R. S. Baric , A. C. Sims , K. Pyrc , W. Li , J. Sui , Human coronavirus HKU1 spike protein uses O-acetylated sialic acid as an attachment receptor determinant and employs hemagglutinin-esterase protein as a receptor-destroying enzyme. J. Virol. 89, 7202–7213 (2015). 10.1128/JVI.00854-15 25926653 PMC4473545

[104] I. G. Madu , V. C. Chu , H. Lee , A. D. Regan , B. E. Bauman , G. R. Whittaker , Heparan sulfate is a selective attachment factor for the avian coronavirus infectious bronchitis virus Beaudette. Avian Dis. 51, 45–51 (2007). 10.1637/0005-2086(2007)051[0045:HSIASA]2.0.CO;2 17461266

[105] C. Winter , C. Schwegmann-Weßels , D. Cavanagh , U. Neumann , G. Herrler , Sialic acid is a receptor determinant for infection of cells by avian Infectious bronchitis virus. J. Gen. Virol. 87, 1209–1216 (2006). 10.1099/vir.0.81651-0 16603523

[106] W. Li , R. J. G. Hulswit , S. P. Kenney , I. Widjaja , K. Jung , M. A. Alhamo , B. van Dieren , F. J. M. van Kuppeveld , L. J. Saif , B.-J. Bosch , Broad receptor engagement of an emerging global coronavirus may potentiate its diverse cross-species transmissibility. Proc. Natl. Acad. Sci. U.S.A. 115, E5135–E5143 (2018). 10.1073/pnas.1802879115 29760102 PMC5984533

