# Supplementary material for: ACE2-binding exposes the SARS-CoV-2 fusion peptide to broadly neutralizing coronavirus antibodies
Source: Science. 2022 Jul 12:eabq2679. doi: 10.1126/science.abq2679 (PMC9348755; doi:10.1126/science.abq2679)
Supplement: Supplementary file 1 — Materials and Methods Figs. S1 to S8 Tables S1 to S5 References ( 71 – 106 ) [file science.abq2679_sm.pdf]

Science
AAAS

Supplementary Materials for

ACE2-binding exposes the SARS-CoV-2 fusion peptide to broadly neutralizing coronavirus antibodies

Jun Siong Low *et al.*

Corresponding authors: Federica Sallusto, sallustf@ethz.ch; Jun Siong Low, junsiong.low@biol.ethz.ch; David Veesler, dveesler@uw.edu

DOI: 10.1126/science.abq2679

The PDF file includes:

Materials and Methods
Figs. S1 to S8
Tables S1 to S5
References

Other Supplementary Material for this manuscript includes the following:

MDAR Reproducibility Checklist (.pdf)

Materials and Methods

Samples and donors

Convalescent samples were obtained from individuals with prior SARS-CoV-2 infection, validated either by positive PCR test or by anti-spike IgG serology. Samples from vaccinated individuals (naïve or pre-immune) were collected 14-29 days after the second booster shot of Pfizer/BioNTech BNT162b2 vaccine (**table S5**). The study protocols were approved by the Cantonal Ethics Committee of Ticino, Switzerland (CE-TI-3428, 2018-02166; CE-TI-3687, 2020-01572). All blood donors provided written informed consent for participation in the study. Human primary cell protocols were approved by the Federal Office of Public Health (no. A000197/2 to F.S.).

Cell lines and culture media

HEK293T and A549 cells were cultured in high glucose DMEM (Gibco, catalog no. 61965-026) supplemented with 10% FBS, 1% (vol/vol), nonessential amino acids, 1% (vol/vol), sodium pyruvate and PenStrep (50 U/ml penicillin, 50 μg/ml streptomycin). HuH-7 cell line was obtained from JCRB Cell Bank and cultured in DMEM (Gibco, catalog no. 31885-023) supplemented with 10% FBS, 1% (vol/vol), nonessential amino acids, 2 mM glutamine and PenStrep (50 U/ml penicillin, 50 μg/ml streptomycin). Vero-TMPRSS2 cells were cultured in DMEM (Gibco, catalog no. 11995-040) supplemented with 10% FBS (VWR, catalog no. 97068-085) and PenStrep (Gibco, catalog no. 15140-122) (*41*). Expi293F™ cells (ThermoFisher Scientific) were grown in DMEM (Gibco, catalog no. 11965-0) supplemented with 10% FBS and PenStrep (50 U/ml penicillin, 50 μg/ml streptomycin).

Generation of stable cell lines

pLVX-puro-ACE2 transfer plasmid was kindly provided by Manfred Kopf (ETH Zurich). pLVX-EF1a-TMPRSS2-IRES-ZsGreen1 transfer plasmid was generated from the reference pWPI-IRES-Bla-Ak-TMPRSS2 plasmid (Addgene, catalog no. 154982). pLVX-puro-spike transfer plasmid was generated from reference pHDM-SARS-CoV-2 spike (BEI resources, catalog no. NR-52514). Stable cell lines were generated using VSV-based lentivirus transduction. Briefly, HEK293T cells at 70-80% confluency in T75 flask were co-transfected

with transfer plasmids encoding genes of interest (ACE2, TMPRSS2 or SARS-CoV-2 S) and packaging plasmid psPax2 and envelope plasmid pMD.2G with polyethylenimine (PEI) (at a ratio of 1:2.3 DNA:PEI) (Polysciences, catalog no. 24765-2) in OptiMEM (Life Technologies Europe BV, catalog no. 31985047). Supernatants containing lentiviral particles were harvested 36 h post-transfection, filtered through 0.22 µm filter and precipitated using 40% (W/V) PEG-8000 (Promega, catalog no. V3011) and 1.2M NaCl (Sigma-Aldrich, catalog no. 71380) for 4-6 h on a shaker at 4°C, and then centrifuged for 1h at 1,600 g at 4ºC. The lentivirus-containing pellet was resuspended in 100 µl media and was used to transduce HEK293T or A549 cell lines. HEK293T-ACE2, 293T-S, A549-ACE2 and A549-S cell lines were selected using 10 µg/ml puromycin (InvivoGen, catalog no. ant-pr-1) 4 days post-transduction. HEK293T-ACE2-TMPRSS2-GFP cell line was generated from HEK293T-ACE2 cells by subsequent transduction of pLVX-EF1a-TMPRSS2-IRES-ZsGreen1-containing lentivral prep and sorted using BD FACSAria III. A549-ACE2-TMPRSS2 and Huh7-TMPRSS2 stable cell lines were generated using commercial lentivirus (Addgene, catalog no. 154982-LV) and selected using 10 µg/ml blasticidin (InvivoGen, catalog no. ant-bl-1) 4 days post-transduction.

Protein expression and purification

For monoclonal antibodies and Fab expression and purification, Expi293 (Gibco) cells were transiently transfected with heavy and light chain expression vectors, as previously described (*71*). The mAbs S309 and S2X58 were produced by Vir Switzerland using a similar protocol (*9*, *10*). Affinity purification was performed on ÄKTA Pure 25 (Cytiva) operated by UNICORN 6.4, using HiTrap Protein A columns (Cytiva, catalog no. GE17-5079-01) for human IgG1 and CaptureSelect™ CH1-XL (Thermo Fisher, catalog no. 494346201) for Fab fragments. Buffer exchange to PBS was performed with a HiPrep 26/10 Desalting (Cytiva, catalog no. GE17-5087-01). The final products were sterilized by filtration through 0.22 µm filters and stored at 4ºC.

SARS-CoV-2 S HexaPro gene was synthetized by GenScript containing the following six stabilizing mutations: K986P, V987P, F817P, A892P, A899P, A942P (*33*) in a codon optimized gene with a C-terminal his-tag for purification and cloned in pcDNA3.1 (-) as previously described (*12*). Briefly, 200 ml of Expi293F cells at 3 × 10^6/ml were transiently transfected with 200 µg of the respective plasmid mixed with 640 µl of ExpiFectamine 293 Reagent, following the manufacturer’s instructions. Four days post-transfection, supernatants were clarified by

centrifugation at 800 g for 10 min, supplemented with 20 mM imidazole, 300 mM NaCl and 25 mM Tris-HCl pH 8.0, further centrifuged at 14,000 g for 30 min and passed through a 1 ml His trap HP column (Cytiva) previously equilibrated with binding buffer (25 mM Tris pH 7.4 and 350 mM NaCl). Proteins were eluted using a linear gradient of 500 mM imidazole using an AKTA Xpress FPLC (Cytiva) operated by UNICORN software version 5.11 (Build 407).

SARS-CoV-2 S PentaPro gene was synthetized by GenScript, codon optimized for mammalian expression and with all the stabilizing mutations of the Hexapro construct except for the F817P mutation and cloned into pcDNA3.1 (-) expression vector. SARS-CoV-2 S PentaPro was expressed and purified following the same protocol as described for SARS-CoV-2 S HexaPro.

SARS-CoV-2 S_2 in postfusion conformation was produced as previously described (*72*) starting from a SARS-CoV-2 S (D614G) ectodomain trimer construct synthetized by GenScript with a mutated S_1/S_2 cleavage site ($_{681}$PRRAR$_{685}$ to $_{681}$PSGAR$_{685}$) and comprising residues Q14 to K1211 followed by a TEV cleavage site, a foldon trimerization motif and an 8× His tag. Briefly, SARS-CoV-2 D614G S ectodomain was produced in Expi293F cells grown in suspension using Expi293 expression medium (Life Technologies) according to manufacturer's instruction. Cell cultures at 3×10^6/ml were transiently transfected using Expifectamine (Life Technologies) following manufacturer's instructions. Three days post-transfection, supernatant was clarified and the S glycoprotein ectodomain was affinity purified using a 1 ml HisTrapHP column (Cytiva). To isolate postfusion, S glycoprotein ectodomain was incubated with the S2X58 triggering Fab (*10*) and 1 μg/ml trypsin. After 1 h incubation, the reaction mixture was further purified using Superose 6 Increase 10/24 column (Cytiva). Purified SARS-CoV-2 S_2 in postfusion conformation, was concentrated, buffer exchanged into PBS (137 mM NaCl, 2.7 mM KCl, 10 mM Na_2HPO_4, and 1.8 mM KH_2PO_4) and quantified using absorption at 280 nm.

The gene to express SARS-CoV-2 S_2 in prefusion conformation was synthetized by GenScript, codon optimized for mammalian expression and includes an N terminal mu phosphatase signal peptide and residues 686 to 1208 from SARS-CoV-2 Wuhan-Hu-1 S in which the following stabilizing mutations were included: Y707C/T883C, A892P, A899P, A942P, K986P and V987P, disulfide F970C-G999C (*33*, *72–74*). The gene was fused to a foldon trimerization domain followed by a C terminal avi- and his-tag. Expression and purification were carried out from 200 ml of Expi293F cells. Expi293F cells grown to a density of 3×10^6/ml

were transiently transfected with 640 µl of Expifectamine (Thermo Fisher) and 200 µg of the corresponding expression plasmid. Cells were grown according to manufacturer's instruction. Four days post-transfection, supernatants were clarified by centrifugation at 800 g for 10 min, supplemented with 350 mM NaCl and 25 mM Tris-HCl pH 8.0, further centrifuged at 14,000 g for 30 min and passed through a 1 ml His trap HP column (Cytiva) previously equilibrated with binding buffer (25 mM Tris pH 7.4 and 350 mM NaCl). SARS-CoV-2 S_2 PentaPro was eluted using a linear gradient of 500 mM imidazole. Purified protein was concentrated, buffer exchanged into 25 mM Tris pH 8, 150 mM NaCl, and quantified using absorption at 280 nm.

Enzyme-linked immunosorbent assay (ELISA)

Costar 96-well half-area plates with high protein binding treatment (Corning, catalog no. 3690) or custom made 384-well high-binding plates (Perkin Elmer) were coated overnight at 4ºC with 2.5 µg/ml of the commercially available recombinant proteins (in PBS) purchased from Sino Biological Inc: SARS-CoV-2 (2019-nCoV) S protein (S_1+S_2 ECD, catalog no. 40589-V08B1), SARS-CoV (S577A, Isolate Tor2) S protein (S_1+S_2 ECD, catalog no. 40634-V08B), MERS-CoV S protein (S_1+S_2 ECD, catalog no. 40069-V08B), OC43 S protein (S_1+S_2 ECD, catalog no. 40607-V08B), HKU1 (isolate N5) S protein (S_1+S_2 ECD, catalog no. 40606-V08B), NL63 S protein (S_1+S_2 ECD, catalog no. 40604-V08B), 229E S protein (S_1+S_2 ECD, catalog no. 40605-V08B), SARS-CoV-2 (2019-nCoV) S_1-His protein (catalog no. 40591-V08H), SARS-CoV-2 (2019-nCoV) S_2 ECD-His protein (catalog no. 40590-V08B), SARS-CoV-2 (2019-nCoV) RBD-His (catalog no. 40592-V08H), Influenza A H1N1 (A/California/4/2009) Hemagglutinin HA-His (catalog no. 11055-V08B1); or with 8 µg/ml of synthetic peptides of indicated sequences: TPPIKDFGGFNFSQI, DFGGFNFSQILPDPS, NFSQILPDPSKPSKR, LPDPSKPSKRSFIED, KPSKRSFIEDLLFNK, SFIEDLLFNKVTLAD, LLFNKVTLADAGFIK, VTLADAGFIKQYGDC, AGFIKQYGDCLGDIA for epitope mapping; or with a peptides of the indicted sequences: KPSKRSFIEDLLFNK (SARS-CoV-2), KPTKRSFIEDLLFNK (SARS-CoV), SRSARSAIEDLLFDK (MERS-CoV), KASSRSAIEDLLFDK (OC43), GSSSRSLLEDLLFNK (HKU1), RIAGRSALEDLLFSK (NL63), RVAGRSAIEDILFSK (229E), SPRRRSFIEDLLFTS (IBV), RLGGRSAIEDLLFNK (PdCOV); or with 1 µg/ml of SARS-CoV-2 prefusion and postfusion proteins. Plates were subsequently washed and blocked with Blocker Casein in PBS (Thermo Fisher Scientific, catalog no. 37528) supplemented with

0.05% Tween 20 (Sigma Aldrich, catalog no. 93773) for 1 h at room temperature. For primary antibody incubations, in serological ELISA, the coated plates were incubated with 25 µl of serial 1:3 dilutions of human plasma (12-point dilutions starting at 1:20 in Casein), for 1 h at room temperature; in cellular profiling ELISA, the coated plates were incubated with 5 µl of undiluted supernatant for 1 h at room temperature. The plates were then washed with PBS containing 0.1% Tween-20 (PBS-T), and Alkaline Phosphatase-conjugated Goat Anti-Human IgG (dilution 1:500, catalog no. 2040-04) from Southern Biotech was added and incubated for 45 min at room temperature. Plates were washed three times with PBS-T, and 4-NitroPhenyl Phosphate (pNPP, Sigma-Aldrich, catalog no. N2765) substrate was added and the absorbance of 405 nm was measured by a microplate reader (BioTek). ED50 (serum dilution) values were extrapolated by nonlinear regression curve fit (4PL) using GraphPad Prism 9 software.

For PentaPro and HexaPro S ELISA, 30 µl of the proteins at 5 ng/µl were plated onto 384-well Nunc Maxisorp (Thermo Fisher) plates in PBS and sealed overnight at room temperature. The next day, plates were washed 4 × in Tris-Buffered Saline Tween (TBST) using a plate washer (BioTek) and blocked with Casein for 1 h at 37°C. Plates were washed 4 × in TBST and 1:4 serial dilutions of the corresponding mAbs starting from 0.3 mg/ml were made in 30 µl TBST, added to the plate and incubated at 37°C for 1 h. Plates were washed 4 × in TBST and 30 µl of anti-human (Invitrogen) horseradish peroxidase-conjugated antibodies diluted 1:5,000 was added to each well and incubated at 37°C. After 1 h, plates were washed 4 × in TBST and 30 µl of TMB (SeraCare) was added to every well for 5 min at room temperature. For SARS-CoV-2 S_2 in prefusion and postfusion conformation, the same protocol was followed except that 30 µl at 3 ng/µl of purified proteins were plated onto 384-well Nunc Maxisorp plates in PBS and sealed overnight at 4ºC. Reactions were quenched with the addition of 30 µl of 1 N HCl. Plates were immediately read at 450 nm on a BioTek plate reader and data plotted and fit in Prism 9 (GraphPad) using nonlinear regression sigmoidal, 4PL, X is the concentration to determine EC50 values from curve fits.

Cellular profiling of memory B cell repertoire analysis

Peripheral blood mononuclear cells (PBMCs) were isolated by Ficoll-Paque Plus (Cytiva, catalog no. 17-1440-03) gradient and seeded in replicative cultures of 96-well U-bottom plates (Corning, catalog no. 3799) at 10^4 cells/well or 3×10^4 cells/well. Cells were stimulated with 2.5

μg/ml of R848 (Invivogen, catalog no. tlrl-r848-5) in complete medium (RPMI 1640 medium (catalog no. 31870-025) supplemented with 2 mM glutamine (catalog no. 35050-038), 1% (vol/vol) nonessential amino acids (catalog no. 11140050, 1% (vol/vol) sodium pyruvate (catalog no. 11360-039), PenStrep (50 U/ml penicillin, 50 μg/ml streptomycin, catalog no. 15070-063), Kanamycin (50 U/ml, catalog no. 15160-047), 0.1% beta-mercaptoethanol (catalog no. 31350-010) (all from Gibco), 10% Hyclone (Cytiva, catalog no. SH30070.03), 0.5% Transferrin (30 μg/ml) (LubioScience, catalog no. 0905-100), and 1,000 U/ml IL-2 (produced in house from transfected J558L cells). Cells were cultured at 37°C, 5% CO_2 for six days and 5 μl of undiluted supernatant were used for ELISA (primary screening). Cultures containing antibodies with binding-profile-of-interest were selected, washed with MACS buffer (PBS with 5% FBS and 2 mM EDTA), and stained with CD19-PE-Cy7 (BD, catalog no. 341113, 1:100), IgM-AF647 (Jackson Immuno, catalog no. 109-606-129, 1:500) and IgA-AF488 (Jackson Immuno, catalog no. 109-546-011, 1:500). IgG^{+} memory B cells were isolated by negative gating strategy as CD19^{+} IgM^{-} IgA^{-} and cloned by limiting dilutions at 0.7 cells/well in complete medium. Two days later, the supernatants of the clones were screened by secondary screening ELISA to validate the binding profile.

BCR retrieval and molecular cloning

Clones with binding-profile-of interest after secondary screening were selected and their VH and VL sequences were obtained by reverse transcription PCR (RT-PCR) using Superscript III (Thermo Fisher, catalog no. 18080044) according to manufacturer’s instruction, using the following VH, VK and VL-specific RT primers: HuIgG-const-anti 5’-TCTTGTCCACCTTGGTGTTGCT-3’; Hu-CK 5’-ACACTCTCCCCTGTTGAAGCTCTT-3’ and Hu-CL 5’-ACTGTCTTCTCCACGGTGCT-3’. VH and VK/L sequences were amplified in two nested PCR reactions using adapted VH/VK/VL primers (*75*). To facilitate high-throughput molecular cloning of BCR sequences into the VH/VK/VL expression vectors, the internal primers contain complementary 30 nucleotides of the respective expression vectors in order to be ligated by Gibson reaction using NEBuilder® HiFi DNA Assembly Master Mix (New England Biolabs, catalog no. E2621L). Ligated products were transformed into One Shot Stbl3 competent *E. coli* (Thermo Fisher, catalog no. C737303). Cloned VH, VK and VL plasmids were validated by Sanger sequencing.

Inference for unmutated common ancestor (UCA)

UCA inference were performed on both heavy and light chains for each mAb. Data were processed and analyzed using the Immcantation Framework (http://immcantation.org) with Change-O v1.0.2. First, the sequences were annotated using IgBlast version 1.16 (*76*) and IMGT as reference sequences (*77*). Second, clones were assigned based on IGHV genes, IGHJ gene, and junction distance with the Change-O DefineClones function. Germlines were then reconstructed using the Change-O CreateGermlines function. Finally, the phylogenic trees of each clone with their complete UCA sequences were generated with Igphyml (*78*).

Negative-stain electron microscopy

Three micromolar of the aforementioned purified SARS-CoV-2 S (D614G) ectodomain trimer were incubated with 3 µM of fusion peptide Fab for 1 h at room temperature prior the addition of 2.6 µM of S2X58 Fab. Incubation was continued at room temperature for 1 h after which samples were diluted to 0.01 mg/ml immediately before protein was adsorbed to glow-discharged manually carbon-coated copper grids for ~30 s before 2% uranyl formate staining. Micrographs were recorded using the Leginon software (*79*) on a 120kV FEI Tecnai G2 Spirit with a Gatan Ultrascan 4000 4k × 4k CCD camera at 67,000 nominal magnification. The defocus ranged from 2.0 to 4.0 µm and the pixel size was 1.6 Å.

Epitope substitution scan

Epitope substitution scan was performed by PEPperPRINT GmbH, Heidelberg, Germany. Briefly, each amino acid in the fusion peptide sequence $K_{811}PSKRSFIEDLLFNKVTLAD_{830}$ was substituted stepwise with all 20 main amino acids. These peptide variants and wildtype peptide, as well as HA control peptides was printed on a microarray chip in triplicate. Primary antibodies at 1 µg/ml (C13B8 and C13C9) or 100 µg/ml (VN01H1 and VP12E7) were incubated with the microarray chip for 16 h at 4ºC with orbital shaking at 140 rpm. After washing, secondary antibody goat anti-human IgG (H+L) DyLight680 (0.2 µg/ml) was incubated for 45 min at room temperature before reading on Innopsys InnoScan 710-IR Microarray Scanner.

Generation of recombinant ACE2-mFc

Residues 18-615 of human ACE2 (UniProtKB - Q9BYF1) were synthesized by Genscript and cloned into pINFUSE-mIgG2b-Fc2 expression plasmid (InvivoGen). Recombinant protein was produced by transient transfection of Expi293 cells and purified using HiTrap Protein A column. Buffer exchange was performed using HiPrep 26/10 Desalting column and final product was sterilized through a 0.22 µm filter.

Pseudotyped virus production

To produce SARS-CoV-2, SARS-CoV, MERS-CoV and 229 S pseudotyped virus, full-length spike-encoding plasmids were obtained from the following manufacturers: SARS-CoV-2 Wuhan-Hu-1 (catalog no. NR-52514) from Bei Resources; SARS-CoV (VG40150-G-N), MERS-CoV (VG40069-G-N) and 229E (VG40605-UT) from SinoBiological. NL63 (YP_003767.1) and WIV-1 (Uniprot–U5WI05) S full-length genes were synthesized from GenScript. The gene encoding PDF-2180 S (YP_009361857.1) full-length was synthesized by GenScript, comprises residues 1 to 1345 and was cloned into pcDNA3.1 (-).

HIV-based hCoV spike glycoprotein-pseudotyped viruses were prepared as previously described (*80*) with slight modifications. Briefly, HEK293T cells were co-transfected with a lentiviral backbone encoding luciferase reporter (pHAGE-CMV-Luc2-IRES-ZsGreen-W (Bei Resources, catalog no. NR-52516), HIV-based packaging plasmids (Tat, Gag-Pol and Rev) (Bei Resources, catalog no. NR-52518, NR-52517 and NR-52519) and various spike expression plasmids using PEI in OptiMEM. Supernatants were harvested 36 h post-transfection and pseudotyped viral particles were precipitated as described above.

To produce NL63, PDF-2180, Wuhan-Hu-1 and Omicron S pseudotyped VSV virus, HEK293T cells were transfected with a pcDNA3.1 expression vector encoding full-length S harboring a truncation of the 20 C-terminal residues to improve membrane transport except for PDF-2180 which did not have a truncation. The day after transfection, cells were transduced with VSVΔG/Luc (*81*). After 2 h, infected cells were washed four times with DMEM before adding medium supplemented with anti-VSV-G antibody (I1- mouse hybridoma supernatant diluted 1 to 25, from CRL- 2700, ATCC). Supernatant was harvested 18-24 h post inoculation, clarified from cellular debris by centrifugation at 2,000 g for 5 min, filtered using a 0.45 µm membrane,

concentrated 10 times using a 30 kDa cut off membrane (Amicon), aliquoted and frozen at -80°C until use.

Pseudotyped virus neutralization

For SARS-CoV-2, SARS-CoV, MERS-CoV, 229E, NL63 and WIV-1 S pseudotyped virus neutralization assay, target cells (HEK293T-ACE2 or HEK293T-ACE2-TMPRSS2 for SARS-CoV-2 and SARS-CoV; HuH-7-TMPRSS2 for MERS-CoV and 229E; HEK293T-ACE2-TMPRSS2 for WIV-1) were seeded in white 96-well plate (Perkin Elmer, catalog no. 6005688) at 40,000 cells/well. The day after, concentrated viruses were titrated in serial dilutions with the respective target cell lines and the luciferase reporter signal was determined 48 h later using Luciferase Assay System (Promega, catalog no. E1501) on Cytation 3 (BioTek). Virus concentrations that gave signal higher than 10^5 RLUs/well were used in neutralization experiments. Serial 1:3 dilutions of mAbs (10-point dilutions starting at 200 μg/ml) were pre-incubated with equal volumes of pseudotyped viruses at 37°C for 30 min. The pseudotyped virus-mAb mixture were then overlayed onto target cell lines in the presence of 5 μg/ml polybrene (Sigma Aldrich, catalog no. TR-1003-G) and analyzed 48 h post-infection. For VSV-Wuhan-Hu-1 and NL63 S pseudotyped virus neutralization assay, VeroE6-TMPRSS2 cells maintained in DMEM supplemented with 10% FBS and 1% PenStrep, were seeded into white 96-well plates at 45,000 cells/well and cultured overnight at 37°C. Eleven-point 3-fold serial dilutions of the corresponding mAbs were prepared in DMEM. For PDF-2180 S pseudovirus neutralization assays, two 10 cm^2 dishes at 70-90% confluence of HEK293T cells were transfected with 24 µg of a plasmid encoding full-length human ACE2 (Addgene, catalog no. 1786) mixed with 60 µl of lipofectamine 2000. Five hours post-transfection, cells were trypsinized and seeded in white 96-well plate at 40,000 cells/well. PDF-2180 and NL63 S pseudotyped viruses were added 1:1 (v/v) to each dilution (final volume 50 μl) and the mixtures were incubated at 37°C. After 45-60 min incubation, 40 μl of each reaction mixture were added to the cells which were incubated at 37°C. After 2 h incubation, 40 μl DMEM were added to avoid evaporation and incubation was continued at 37°C. After 17-20 h, 60 μl/well of One-Glo-EX substrate (Promega) were added to the cells and incubated in the dark for 5-10 min prior reading on a Varioskan LUX plate reader (Thermo Fisher). Data was processed using GraphPad Prism v9.0.

Authentic live virus neutralization

SARS-CoV-2 isolates WA-1 (USA-WA1/2020) (BEI ref. NR-52281), BA.1 (hCoV-19/USA/MD-HP20874/2021) (BEI ref. NR-56461) and BA.2 (hCoV-19/USA/MD-HP24556/2022) (BEI ref. NRS-56511) were incubated with anti-fusion peptide mAbs at indicated concentrations prior to infection on target Vero-TMPRSS2 cells at MOI 0.01. Readout was performed by IFA of anti-nucleocapsid at 18 hr (WA-1) or 24 hr (BA.1 and BA.2).

Inhibition of cell-to-cell fusion

For testing inhibition of spike-mediated cell–cell fusion, A549-S and A549-ACE2-TMPRSS2 cells were stained with CFSE (Thermo Fisher, catalog no. C1157) and CellTrace™ Far Red (Thermo Fisher, catalog no. C34572), respectively, according to manufacturer's instruction. Stained cells were resuspended in complete media containing Hoechst 33342 (Thermo Fisher, catalog no. H1399) at a final concentration of 5 µg/ml. A549-S cells were co-cultured in indicated concentrations of mAbs for 30 min at 37°C before addition of stained-A549-ACE2-TMPRSS2. Fusion events were measured 2 h post incubation with Molecular Devices ImageXpress Micro 4 system. Acquisition was performed with a 20x/0.45 Super Plan Fluor ELWD objective, FITC and Cy5 filter and images collected with a Andor Zyla sCMOS camera. Nine fields per well were imaged and were subsequently processed with Metaxpress and Powecore softwares.

Crystallization, data collection, structure determination and analysis

Fabs C13B8, C13C9, C77G12, VN01H1 and VP12E7, at 20 mg/ml in PBS were mixed with the fusion peptide (KPSKRSFIEDLLFNK, GenScript) at 20 mM in 100% DMSO and incubated for 2 h at room temperature before setting up crystallization plates. Crystals of Fabs in complex with the fusion peptide were obtained at 22°C by sitting drop vapor diffusion. A total of 100 nl complex were mixed with 100 nl mother liquor solution containing 0.2M potassium acetate, 20% (w/v) PEG 3350, 0.2 M potassium chloride, 0.05 M HEPES-NaOH pH 7.5, 35% (v/v) pentaerythritol propoxylate 5/4/PO/OH (VN01H1 complex); 0.2 M Calcium Acetate Hydrate, 0.1 M MES NaOH, pH 6.0, 25% PEG 8000 (VP12E7 complex); 0.2 M Calcium Chloride 20 % (w/v) PEG 3350 (C77G12 complex); 0.17 M Ammonium Acetate, 0.085 M Sodium Acetate: HCl, pH 4.6, 25.5 % (w/v) PEG 4000, 15 % (v/v) glycerol (C13B8 complex) and 0.04 M

KH_2PO_4, 16 % (w/v) PEG 8000, 20 % (v/v) Glycerol (C13C9 complex). Drops were equilibrated against reservoir solutions for 1 week at room temperature after which crystals were flash cooled in liquid nitrogen using the mother liquor solution supplemented with 30% glycerol as a cryoprotectant. Data were remotely recorded at the beamline 5.0.1 at the Advanced Light Source synchrotron facility in Berkeley, CA. Individual datasets for each complex were processed with the XDS software package (*82*) and Mosflm (*83*) and scaled using SCALA or aimless (*84*). Initial phases were obtained by molecular replacement in Phaser (*85*) on the CCP4 suite, using crystal structures of Fabs as search models. Several subsequent rounds of model building, and refinement were performed using Coot (*86*), Phenix-Refine (*87*) and Buster (*88*) to arrive to the final model for each complex.

Transient expression and monoclonal antibody staining of hCoV S-expressing HEK293T cells

For transient expression of hCoV S proteins, HEK293T cells were co-transfected, with plasmid encoding ZsGreen (Bei Resources, catalog no. NR-52516) and corresponding hCoVs spike proteins: SARS-CoV-2 Wuhan-Hu-1 S (catalog no. NR-52514) from Bei Resources; MERS-CoV S (VG40069-G-N), 229E S (VG40605-UT), NL63 S (VG40604-UT) from SinoBiological; SARS-CoV S (VG40150-G-N) from SinoBiological that was cloned into pHDM expression plasmid with 19 amino-acid C-terminal truncation (*89*), using PEI in Opti-MEM as above. For the SARS-CoV-2 S2P mutant, K986P and V987P mutations were introduced into the wildtype backbone using Q5 Site-Directed Mutagenesis Kit (NEB, catalog no. E0554S). Transiently transfected cells were stained the following day with mAbs conjugated using DyLight® 650 Conjugation Kit (Fast)-Lightning-Link (Abcam, catalog no. ab201803) according to manufacturer's instructions. Dylight 650-conjugated mAbs (of indicated concentrations) were incubated with 50,000 un-trypsinized HEK293T cells expressing hCoV S in the presence or absence of ACE2 (27 μg/ml), S2E12 (20 μg/ml) (*51*), S2M11 (*51*) (20 μg/ml), DPP4 (27 μg/ml) and APN (27 μg/ml) in MACS buffer. After 2 h at room temperature, cells were washed and analyzed by flow cytometry using BD Symphony and FlowJo.

SARS-CoV-2 infection model in hamsters

KU LEUVEN R&D has developed and validated a SARS-CoV-2 Syrian Golden hamster infection model (*90*, *91*). This model is suitable for the evaluation of the potential antiviral activity and selectivity of novel compounds/antibodies (*92*). The SARS-CoV-2 strain used in the model, Gamma P.1 (EPI_ISL_1091366; 2021-03-08), was recovered from a nasopharyngeal swab taken from a traveler returning to Belgium in March 2021 (*93*). The variant was subjected to sequencing on a MinION platform (Oxford Nanopore) directly from the nasopharyngeal swabs; passage 2 virus on Vero E6 cells was used for the study described here. The titer of the virus stock was determined by end-point dilution on Vero-E6 cells by the Reed and Muench method (*94*). Live virus-related work was conducted in the high-containment A3 and BSL3+ facilities of the KU Leuven Rega Institute (3CAPS), under licenses AMV 30112018 SBB 219 2018 0892 and AMV 23102017 SBB 219 20170589, according to institutional guidelines.

Syrian Golden hamsters (Mesocricetus auratus) were purchased from Janvier Laboratories and were housed per two in ventilated isolator cages (IsoCage N Biocontainment System, Tecniplast) with ad libitum access to food and water and cage enrichment (wood block). The animals were acclimated for 4 days prior to study start. Housing conditions and experimental procedures were approved by the ethics committee of animal experimentation of KU Leuven (license P065-2020). Female hamsters of 6-8 weeks old were anesthetized with ketamine/xylazine/atropine and inoculated intranasally with 50 µl containing 1×10^4 TCID50 SARS-CoV-2 gamma variant (day 0). Animals were prophylactically treated 24 h before infection with VN01H1 (50 mg/kg), C77G12 (25 mg/kg and 50 mg/kg) or negative control anti-malaria mAb MGH2 (*95*) (50 mg/kg) via intraperitoneal (IP) administration. Hamsters were monitored for appearance, behavior, and weight. At day 4 post infection (pi), hamsters were euthanized by IP injection of 500 µl Dolethal (200 mg/ml sodium pentobarbital, Vétoquinol SA). Lungs were collected and viral RNA and infectious virus were quantified by RT-qPCR and end-point virus titration, respectively. Blood samples were collected for pharmacokinetics analysis. In this model, no significant body weight loss was observed at day 4 post infection in untreated SARS-CoV2 infected hamsters despite a fulminant lung infection (*90*, *96*).

SARS-CoV-2 RT-qPCR

Hamster lung tissues were collected after sacrifice and were homogenized using bead disruption (Precellys) in 350 µL TRK lysis buffer (E.Z.N.A.® Total RNA Kit, Omega Bio-tek) and centrifuged (11,000 g, 5 min) to pellet cell debris. RNA was extracted according to the manufacturer's instructions. Of 50 µl eluate, 4 µl was used as a template in RT-qPCR reactions. RT-qPCR was performed on a LightCycler96 platform (Roche) using the iTaq Universal Probes One-Step RT-qPCR kit (BioRad) with N2 primers and probes targeting the nucleocapsid (*90*). Standards of SARS-CoV-2 cDNA (IDT) were used to express viral genome copies per mg tissue or per ml serum.

End-point virus titrations

Lung tissues were homogenized using bead disruption (Precellys) in 350 µl minimal essential medium and centrifuged (11,000 g, 5min, 4ºC) to pellet the cell debris. To quantify infectious SARS-CoV-2 particles, endpoint titrations were performed on confluent Vero E6 cells in 96-well plates. Viral titers were calculated by the Reed and Muench method (*96*) using the Lindenbach calculator and were expressed as 50% tissue culture infectious dose ($TCID_{50}$) per mg tissue.

Histology

For histological examination, the lungs were fixed overnight in 4% formaldehyde and embedded in paraffin. Tissue sections (5 µm) were analyzed after staining with hematoxylin and eosin and scored blindly for lung damage by an expert pathologist. The scored parameters, to which a cumulative score of 1 to 3 was attributed, were the following: congestion, intra-alveolar hemorrhagic, apoptotic bodies in bronchus wall, necrotizing bronchiolitis, perivascular edema, bronchopneumonia, perivascular inflammation, peribranchial inflammation and vasculitis.

Models building and analysis

UCSF Chimera (*97*) and Coot (*86*) were used to align the crystal structures of Fabs-peptide complexes into the cryo-EM model PDB 6VXX. Model validation was done using Molprobity (*98*) and Phenix-Refine (*87*). Figures were generated using UCSF ChimeraX (*99*).

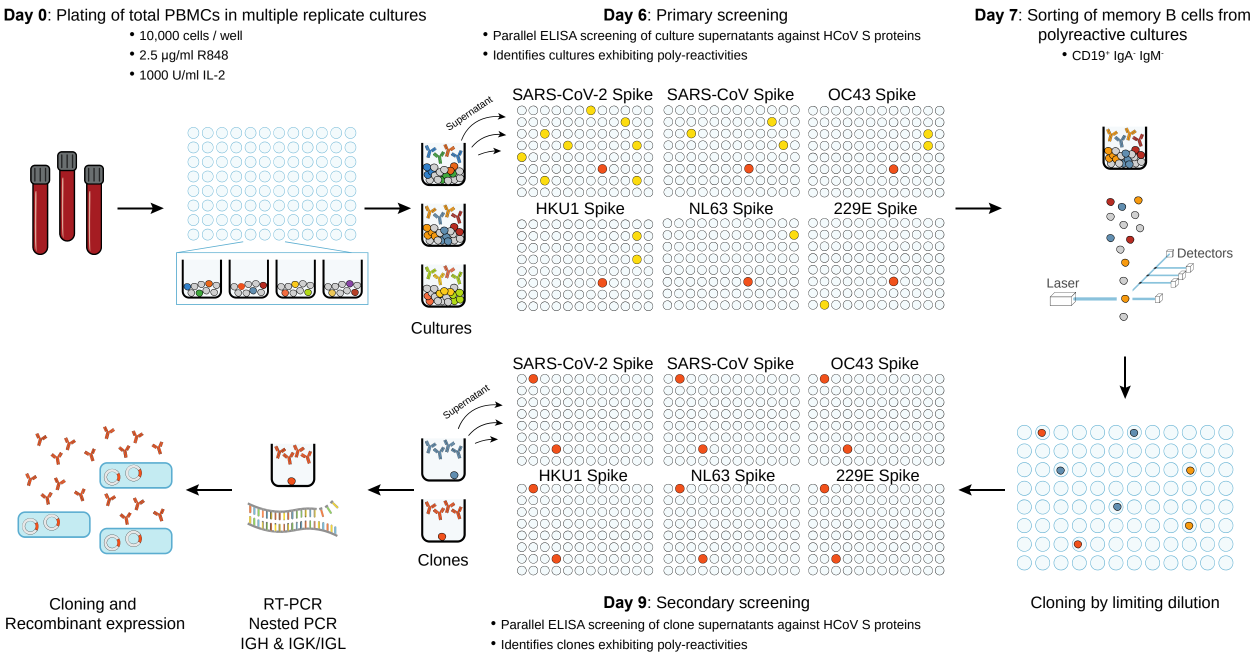

Fig. S1. An improved method that combines high-throughput screening and limiting dilution cloning to isolate B cells of unique characteristics.

Total PBMCs isolated from Ficoll density centrifugation were plated in replicate cultures at densities of 10^4 cells per well in the presence of 2.5 µg/ml of TLR agonist R848 and 1,000 U/ml IL-2. Six days later, the specificities of the secreted IgG antibodies in the culture supernatants from each culture were screened against different hCoVs S antigens in parallel (primary screening). Cultures that exhibit cross-reactive binding patterns (shown as red well) were next isolated as $CD19^+$ IgM^- and IgA^- to enrich for IgG-secreting memory B cell blasts (*100*) and cloned by limiting dilution at 0.7 cell/well. Two days post cloning, the culture supernatants of the clones underwent secondary screening with the same panel of antigens to validate their binding profiles observed during primary screening. Clones which exhibit the same binding profiles as during the primary screening were selected for BCR retrieval by reverse transcription followed by nested PCR reactions. Paired IGH and IGK/IGL were cloned into expression vector and transfected into Expi293 cells for recombinant antibody expression. Recombinantly expressed mAbs were tested for their binding specificities to the S antigens as validation.

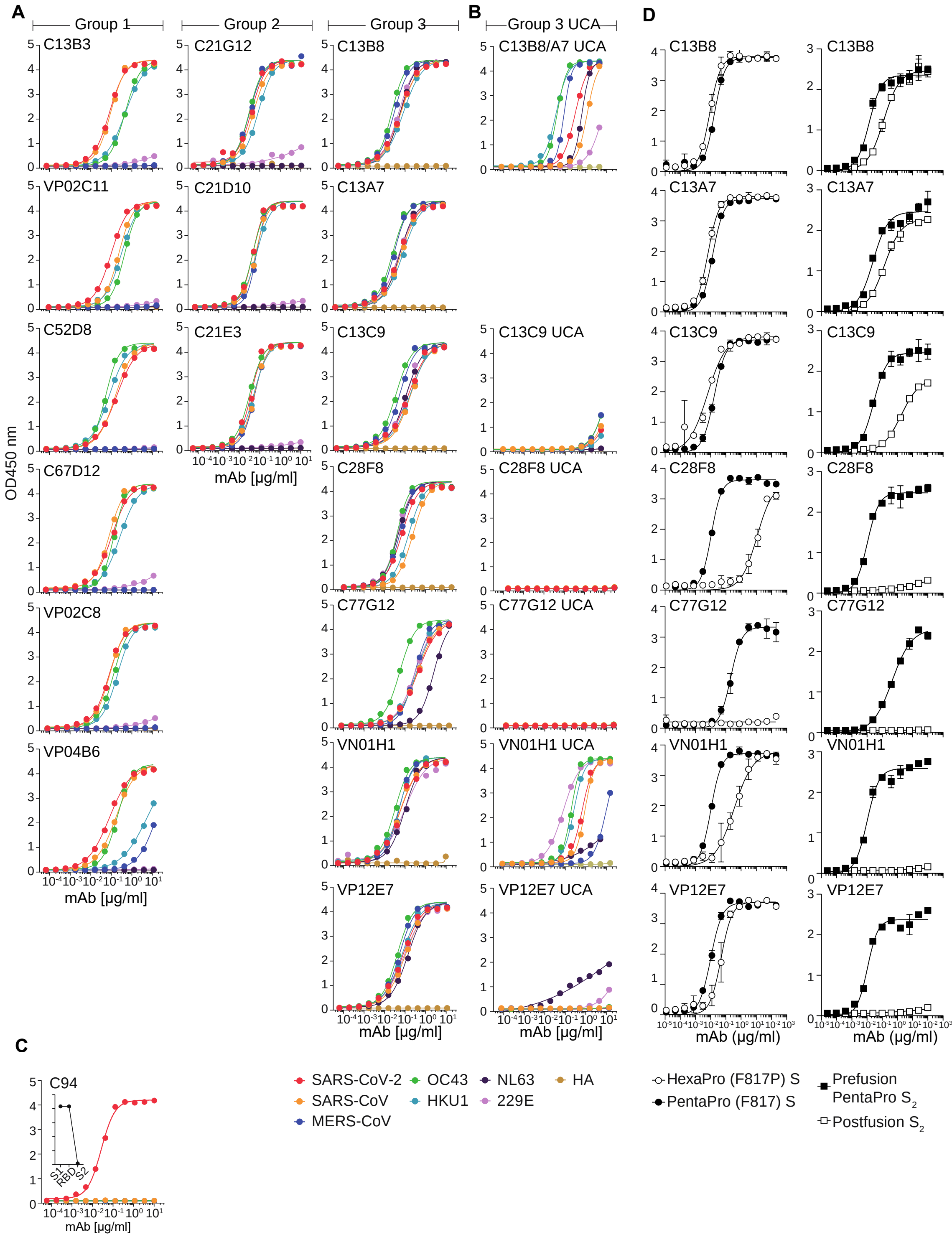

Fig. S2. The binding profiles of all 16 hCoV-cross-reactive mAbs.

(**A** and **B**) Binding profiles of all 16 cross-reactive mAbs (**A**) and respective unmutated common ancestors (UCAs) of Group 3 mAbs (**B**) were tested against immobilized hCoVs S proteins by ELISA. H1N1 haemagglutinin was used as negative control. (**C**) Binding profile of the anti-RBD

mAb C94 was used as a control. Inset represents the domain mapping (S_1, RBD, S_2) of mAb C94 where the relative maximum OD 405 nm value is shown. Data from one representative experiment out of at least two experiments are shown. (**D**) Binding of all seven anti-fusion peptide mAbs to prefusion SARS-CoV-2 S S HexaPro (F817P), SARS-CoV-2 S PentaPro (F817), SARS-CoV-2 S_2 PentaPro in prefusion conformation and SARS-CoV-2 S_2 in postfusion conformation were analyzed by ELISA. One representative experiment out of two is shown.

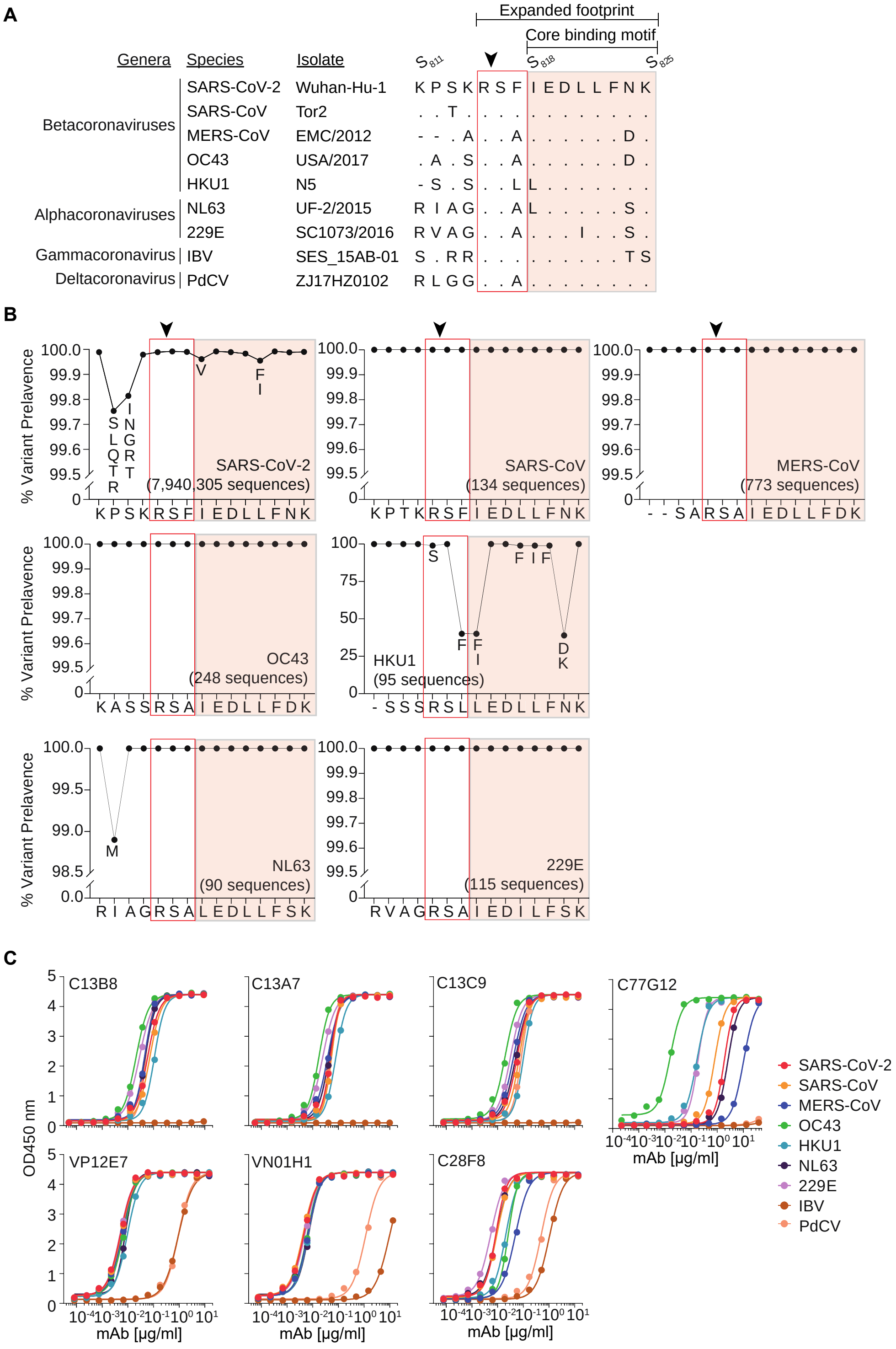

Fig. S3. The fusion peptide epitope is highly conserved among *Orthocoronavirinae* subfamily including circulating SARS-CoV-2 variants

(**A**) Alignment of the fusion peptide motif of alpha-, beta, gamma- and delta- coronaviruses. (**B**) Frequency of mutations in the fusion peptide region is analyzed based on deposited hCoVs sequences at GISAID (SARS-CoV-2) and NCBI virus database (all others), as of 9th February 2022. SARS-CoV-2 (7,940,305 sequences), SARS-CoV (134 sequences), MERS-CoV (773 sequences), OC43 (248 sequences), HKU1 (95 sequences), NL63 (90 sequences), 229E (115 sequences). Arrows indicate S_2' cleavage site. Core binding motif and expanded footprint identified from Fig. S6A are shown. (**C**) Binding profiles of all Group 3 mAbs to alpha, beta, gamma and deltacoronavirus fusion peptides.

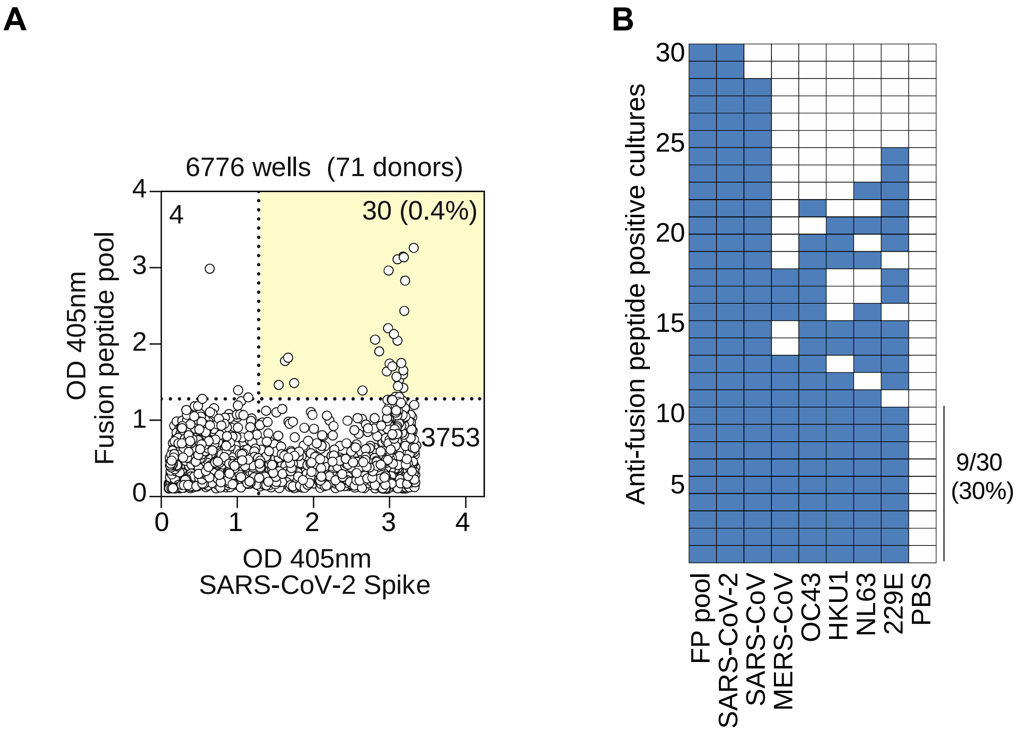

Fig. S4. **Fusion peptide reactive mAbs are rare and only a fraction of them has broad reactivity.**

(**A)** Total PBMCs from 71 COVID-19 convalescent individuals (table S5) were plated in replicate 96 wells (3 × 10^4 cells/well) and stimulated with R848 (2.5 µg/ml) and IL-2 (1,000 U/ml). Six days later, the supernatant of each culture was screened for the specificities of the secreted antibodies to a pool of 15-mer synthetic fusion peptides from SARS-CoV-2, SARS-CoV, MERS-CoV, OC43, HKU1, NL63 and 229E, as well as to SARS-CoV-2 S by ELISA. Each circle in the scatter plot represents one culture and its respective OD 405nm values to fusion peptide pool and to SARS-CoV-2 S. (**B**) Cultures exhibiting reactivities to both fusion peptide pool and SARS-CoV-2 S (yellow quadrant in (**A**)) were further screened for their reactivities to other hCoV S proteins. Each row represents a fusion peptide- and SARS-CoV-2 S-double positive culture and each column shows the reactivities to the indicated S protein antigens. If OD 405nm value exceeds the cut-off value determined by average OD 405nm of PBS wells + 4*standard deviation, the culture was considered reactive to the antigen as indicated by a colored cell.

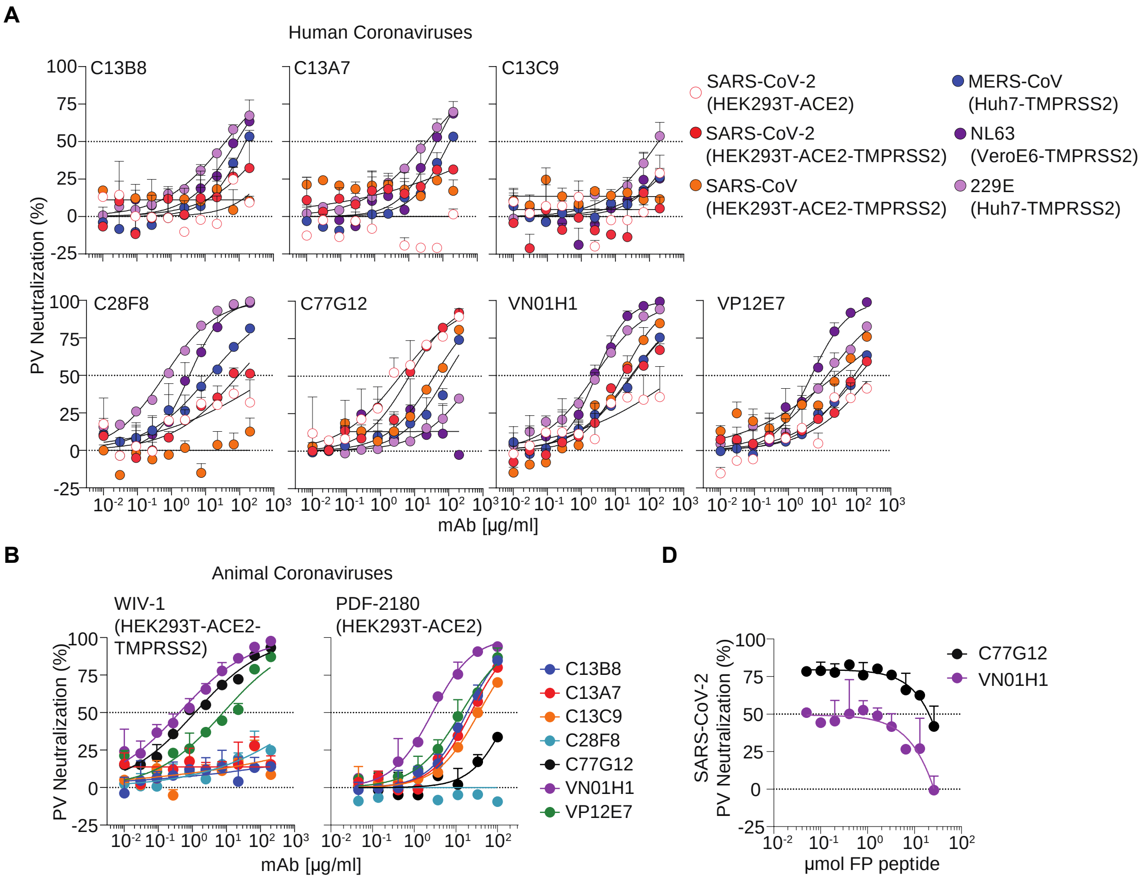

C

Pseudotyped virus	Target cells	IC50 (μg/ml) C13B8	C13A7	C13C9	C28F8	C77G12	VN01H1	VP12E7
SARS-CoV-2	HEK293T-ACE2	N.D.	N.D.	N.D.	N.D.	3.7	N.D.	N.D.
SARS-CoV-2	HEK293T-ACE2-TMPRSS2	N.D.	N.D.	N.D.	91.6	7.0	28.8	113.8
SARS-CoV	HEK293T-ACE2-TMPRSS2	N.D.	N.D.	N.D.	N.D.	37.6	16.1	24.1
MERS-CoV	Huh7-TMPRSS2	155.8	158.9	N.D.	13.2	83.2	31.3	84.4
NL63	VeroE6-TMPRSS2	83.0	65.7	N.D.	3.4	N.D.	2.7	2.7
229E	Huh7-TMPRSS2	40.2	33.0	183.2	0.6	N.D.	2.5	13.6
WIV-1	HEK293T-ACE2-TMPRSS2	N.D.	N.D.	N.D.	N.D.	1.2	0.4	8.0
PDF-2180	HEK293T-ACE2	18.5	23.0	35.5	N.D.	N.D.	2.7	13.5

Fig. S5. Anti-fusion peptide mAbs can neutralize human and animal alpha- and betacoronaviruses.

(**A**) Titrating doses of Group 3 mAbs were assessed for their ability to neutralize SARS-CoV-2, SARS-CoV, MERS-CoV, NL63 and 229E S pseudoviruses in the indicated target cell lines. For each hCoV pseudotyped assay, all mAbs were compared in parallel. (**B**) Titrating doses of pan-

reactive mAbs were assessed for their ability to neutralize the bat sarbecovirus WIV-1 in HEK293T-ACE2-TMPRSS2 cells and the bat merbecovirus PDF-2180 HEK293T cells transfected with full-length human ACE2. (**C**) Summary of IC50 values for each mAb against the indicated pseudotyped coronaviruses. N.D. indicates below neutralizing threshold. (**D**) SARS-CoV-2 neutralizing activity of VN01H1 and C77G12 (200 µg/ml) were competed with titrating doses of soluble fusion peptide.

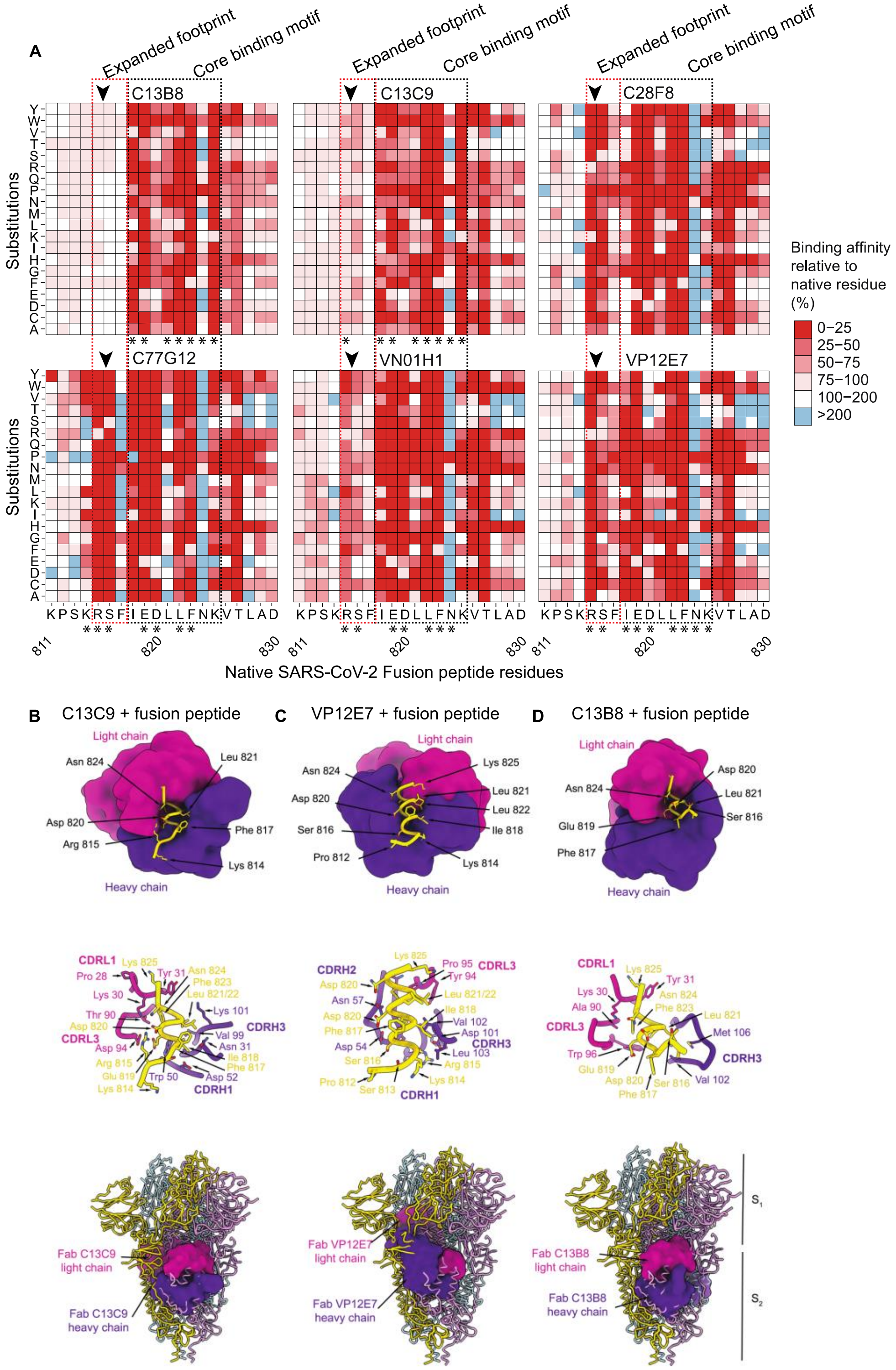

Fig. S6. Varied binding profiles and the cryptic nature of anti-fusion peptide mAb epitopes. (**A**) Heatmaps displaying the substitution scan analysis of six indicated mAbs (C13A7 and C13B8 are clonally related). Each amino acid in the fusion peptide epitope (*x*-axes) was

substituted, stepwise, with all amino acids (*y*-axes), and the binding affinity of the mAb to each peptide variant was measured. Legend shows the binding affinity relative to the native residue. Epitope residues for each mAb defined based on obtained crystal structures are highlighted with asterisks (*). Arrows indicate S_2' cleavage site. Identified core binding motif and expanded footprint are shown. (**B** to **D**) Crystal structures of the C13C9 (**B**), VP12E7 (**C**) and C13B8 (**D**) Fabs (surface representation) in complex with SARS-CoV-2 fusion peptide epitope in ribbon representation (top panels). Ribbon representation of the crystal structures of Fab-bound complexes highlighting the interactions with the CDRs of the Fab heavy and light chains; only selected regions are shown for clarity (middle panels). Alignment between the fusion peptide in SARS-CoV-2 S in prefusion conformation (PDB 6VXX) with the fusion peptide (both in ribbon representation) in the crystal structure of the Fab-bound complex with C13C9, VP12E7 and C13B8 Fabs (surface representation) uncovering the cryptic nature of the epitope (bottom panels). Each SARS-CoV-2 S protomer is colored distinctly (light blue, pink, and gold). Fab heavy and light chains are colored purple and magenta, respectively.

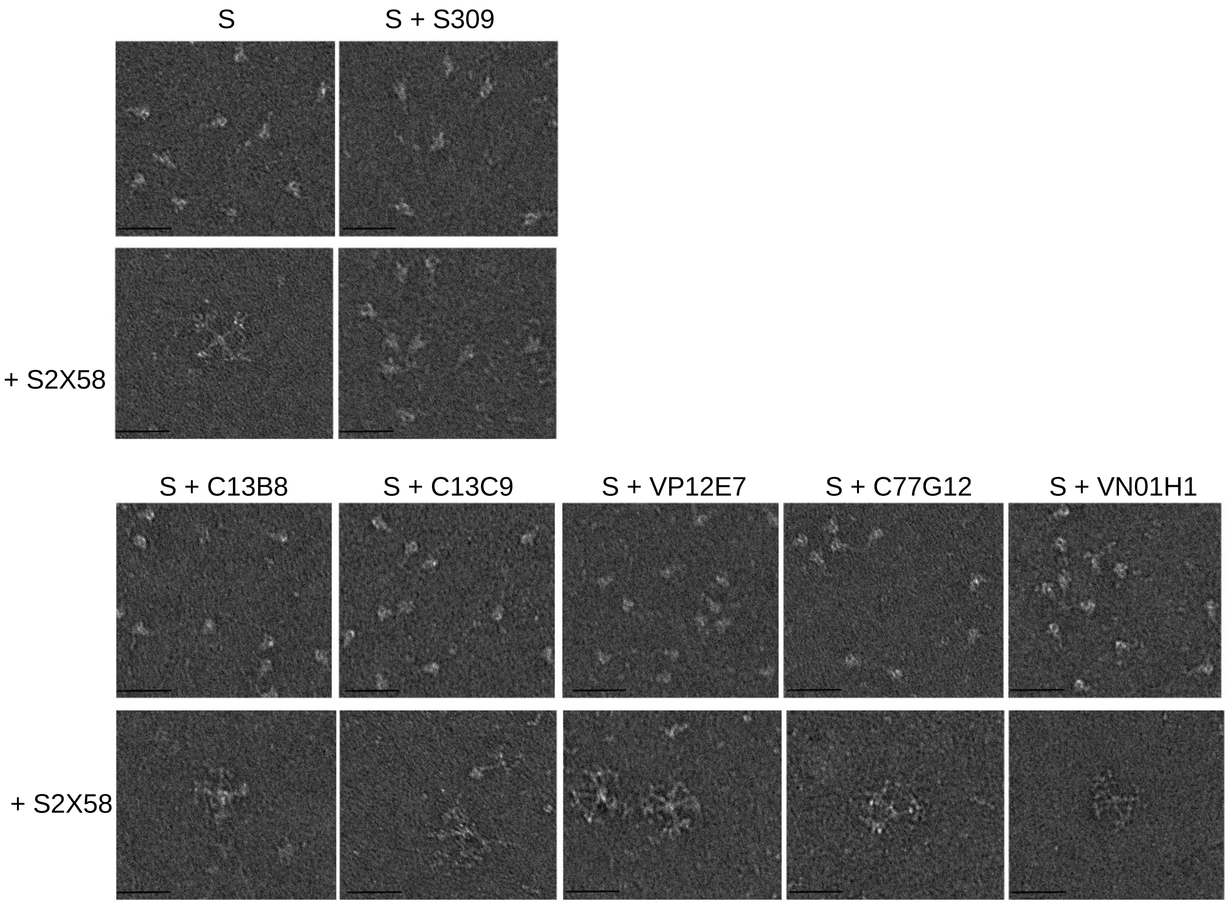

Fig. S7. Fusion peptide-specific Fabs do not block fusogenic rearrangement of the S protein.

Fusion peptide-specific Fabs were incubated for 1 h with native-like soluble prefusion SARS-CoV-2 S trimer prior to addition of the Fab S2X58 to induce fusogenic S rearrangements visible as rosettes by negative stain EM (scale bar: 50 nm). 30 micrographs per sample were analyzed. Negative control: S protein only incubated with S2X58. Positive control: S protein preincubated with Fab S309 before addition of S2X58.

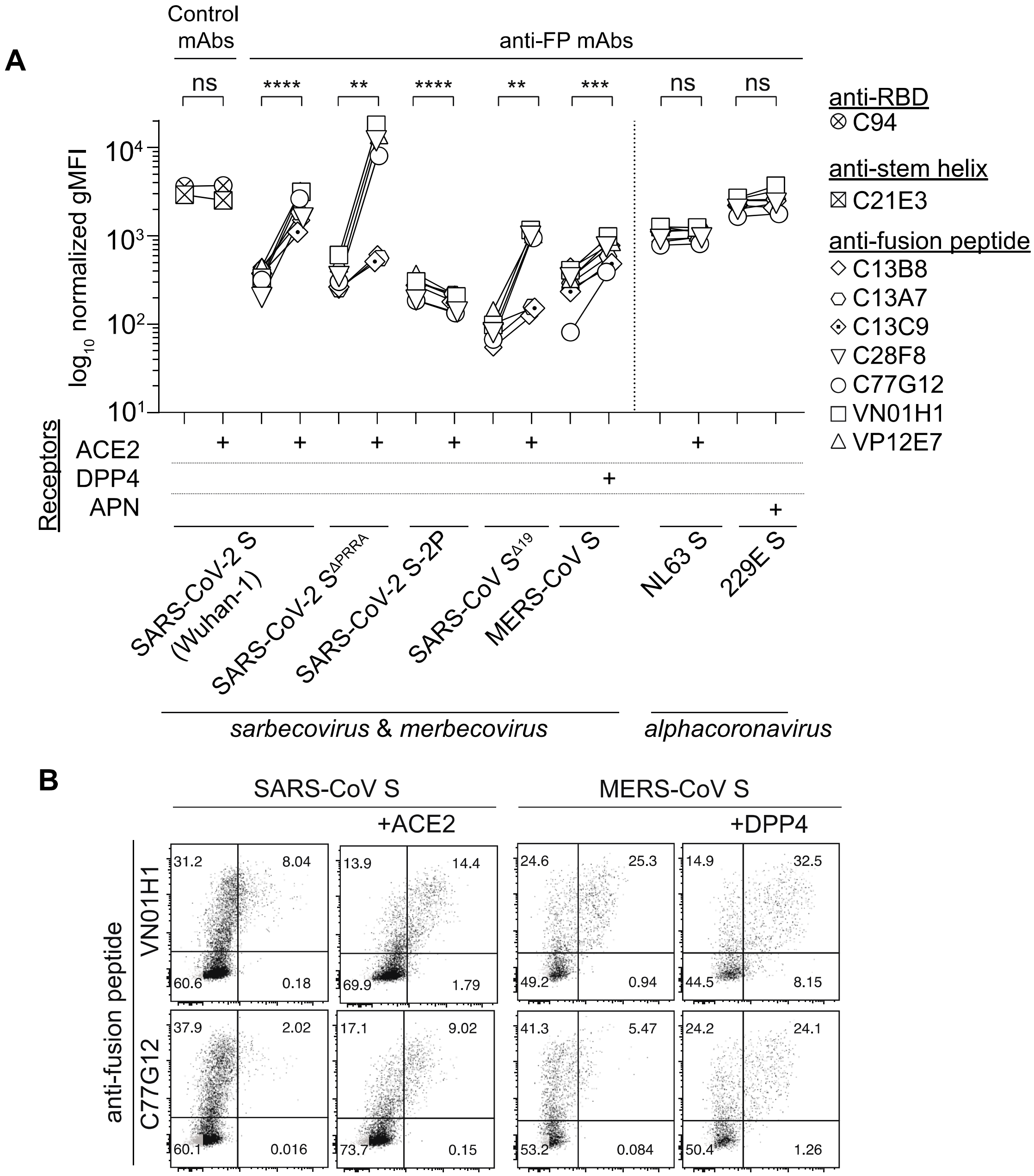

Fig. S8. Receptor binding to SARS-CoV-2, SARS-CoV and MERS-CoV S proteins induces conformational changes that unmask the fusion peptide epitope.

(**A**) Binding of anti-fusion peptide mAbs (8 µg/ml) to HEK293T transiently co-transfected with plasmids encoding ZsGreen and SARS-CoV-2 S, SARS-CoV-2 S$^{\Delta PRRA}$, SARS-CoV-2 S-2P, SARS-CoV$^{\Delta 19}$ S, MERS-CoV S, NL63 S and 229E S, in the presence or absence of receptors ACE2 (27 µg/ml) for SARS-CoV-2, SARS-CoV S and NL63 S, DPP4 (27 µg/ml) for MERS-

CoV S, and APN (27 μg/ml) for 229E S, as measured by flow cytometry. ns $P > 0.05$, **$P \leq 0.01$, ***$P \leq 0.001$, ****$P \leq 0.0001$ Ratio paired *t* test. (**B**) Representative raw binding data of VN01H1 and C77G12 mAb.

Table S1.
Coronaviruses and their receptors mentioned or used in this study

<table>
<tr><th>Genus</th><th>Subgenus</th><th>Virus</th><th>Receptor</th><th>References</th></tr>
<tr><td rowspan="3">Alphacoronavirus</td><td>Setracovirus</td><td>NL63</td><td>ACE2</td><td>(49)</td></tr>
<tr><td>Duvinacovirus</td><td>229E</td><td>APN</td><td>(50)</td></tr>
<tr><td>Tegacovirus</td><td>CCoV-HuPn-2018</td><td>APN</td><td>(1, 3)</td></tr>
<tr><td rowspan="7">Betacoronavirus</td><td rowspan="3">Sarbecovirus</td><td>SARS-CoV-2</td><td>ACE2</td><td>(4, 32)</td></tr>
<tr><td>SARS-CoV</td><td>ACE2</td><td>(47)</td></tr>
<tr><td>WIV-1</td><td>ACE2</td><td>(101)</td></tr>
<tr><td rowspan="2">Merbecovirus</td><td>MERS-CoV</td><td>DPP4</td><td>(48)</td></tr>
<tr><td>PDF-2180</td><td>ACE2</td><td>(40)</td></tr>
<tr><td rowspan="2">Embecovirus</td><td>OC43</td><td>Sialic acid</td><td>(102)</td></tr>
<tr><td>HKU1</td><td>Sialic acid</td><td>(103)</td></tr>
<tr><td>Gammacoronavirus</td><td>Igacovirus</td><td>IBV</td><td>Sialic acid,
heparan sulfate</td><td>(104, 105)</td></tr>
<tr><td>Deltacoronavirus</td><td>Buldecovirus</td><td>PDCoV</td><td>APN</td><td>(2, 106)</td></tr>
</table>

<u>Abbreviations</u>

NL63: NetherLand 63
229E: (named after a student specimen coded 229E)
CCoV-HuPn-2018: Canine Coronavirus Human Pneumonia 2018
SARS-CoV: Severe Acute Respiratory Syndrome Coronavirus
WIV-1: Wuhan Institute of Virology 1
MERS-CoV: Middle East Respiratory Syndrome coronavirus
OC43: Organ Culture 43
HKU1: Hong Kong University 1
IBV: Infectious bronchitis virus
PDCoV: Porcine deltacoronavirus

ACE2: angiotensin-converting enzyme 2
DPP4: dipeptidyl-peptidase 4
APN: aminopeptidase N

Table S2.
Specificity and V gene usage of the seven pan-coronavirus mAbs isolated

	HEAVY CHAIN (IMGT call)						LIGHT CHAIN (IMGT call)			
**mAbID**	**IGHV**	**IGHD**	**IGHJ**	**CDR3 length (aa)**	**CDR3 aa**	**V nucleotide mutations (%)**	**IGKV/IGLV**	**IGKJ/IGLJ**	**CDR3 length (aa)**	**V nucleotide mutations (%)**
**C13B8**	IGHV4-31	IGHD2/OR15-2a	IGHJ4	18	ATSIVLTGMSNKIQPFDY	9.62	IGLV3-16	IGLJ3	10	3.83
**C13A7**	IGHV4-31	IGHD3-9	IGHJ4	18	ATSIVLTGMSNKIQPFDY	10.31	IGLV3-16	IGLJ3	10	3.07
**C13C9**	IGHV1-58	IGHD3-9	IGHJ1, IGHJ4	19	AAVGKDDDVLTGGNKYFDH	14.44	IGLV3-10	IGLJ1	10	6.13
**C28F8**	IGHV1-3	IGHD2-21	IGHJ3	14	ATGGETVWLLAFDI	6.97	IGLV2-14	IGLJ2, IGLJ3	10	2.22
**C77G12**	IGHV3-30-3	IGHD3-10, IGHD4/OR15-4	IGHJ4	15	ARGSDYVDDSPPLHY	11.50	IGKV1-16	IGKJ3	9	7.95
**VN01H1**	IGHV3-64D	IGHD3-3	IGHJ2	16	VKNSDVFRFPHLYFDV	8.68	IGKV3-15	IGKJ5	10	3.03
**VP12E7**	IGHV3-64D	IGHD3/OR15-3	IGHJ3	25	VKGLDVLRFLDLSTPSGERLDAFDI	9.41	IGKV1-9	IGKJ3	10	3.03

Table S3.
X-ray crystallography data collection and refinement statistics

	**VN01H1+peptide**	**VP12E7+peptide**	**C13C9+peptide**	**C13B8+peptide**	**C77G12+peptide**
Space group	C 121	P 1	P 1	P 212121	P 21
Cell dimensions					
a, b, c (Å)	152.92, 60.71, 61.11	71.15, 81.36, 95.65	63.9, 65.7, 71	43.3 69.3 150.7	45.23, 64.06, 78.8
α, β, γ (°)	90, 93.62, 90	90.34, 95.96, 100.65	96.5, 108.06, 110.52	90 90 90	90, 93.3, 90
Resolution range(Å)	46.24-1.86 (1.90-1.86)	48.2-2.5 (2.56-2.50)	44.09-2.1 (2.16-2.1)	41.59-2.1 (2.18-2.1)	45.15-1.7 (1.73-1.7)
CC1/2	0.998 (0.367)	0.992 (0.737)	0.995 (0.661)	0.994 (0.513)	0.999 (0.783)
Rmerge	0.077 (1.6)	0.1 (0.662)	0.061 (0.310)	0.131 (1.117)	0.039 (0.497)
I/σ(I)	6.5 (0.5)	5.0 (1.3)	8.7 (2.3)	7.7 (1.2)	16.4 (1.9)
Completeness (%)	99.7 (99.7)	97.9 (97.6)	97.5 (96.6)	93.6 (98.7)	99.8 (99.7)
Redundancy	3.3 (3.3)	1.8 (1.9)	2.0 (2.0)	4.0 (4.0)	3.7 (3.4)
**Refinement**					
No. reflections	46943	70929	56695	26947	49412
Rwork/Rfree	19.9/23.4	19.5/23.7	17.1/21.2	19/23.9	15.4/18.7
N^{o} of atoms					
Protein	3400	13457	6724	3325	3524
Water	348	701	867	280	548
B factor (Wilson plot)	30.91	38.46	26.53	30.87	21.08
R.m.s. deviations					
Bond lengths (Å)	0.006	0.003	0.002	0.003	0.009
Bond angles (°)	0.85	0.6	0.59	0.69	0.99
Ramachandran favored (%)	98.43	97.9	98.63	98.41	99.1
Ramachandran allowed (%)	1.57	2.1	1.37	1.59	0.9
Ramachandran outliers (%)	0	0	0	0	0
PDB ID	7SKZ	7SL5	7U0E	7U09	7U0A

- Data in parentheses are for the highest resolution shell
- $R_{merge} = \sum(\sum|I_i - \langle I \rangle|/\sum|I|)$, where the first ∑ is the sum over all reflections, and the second ∑ is the sum over all measurements of a given reflection, with I_i being the *i*th measurement of the intensity of the reflection and $\langle I \rangle$ the average intensity of that reflection.
- $R_{work}/R_{free} = \sum(|F_o| - \langle |F_c| \rangle)/\sum|F_o|$, where $\langle |F_c| \rangle$ is the expectation of $|F_c|$ under the probability model used to define the likelihood function. The sum is overall reflections.

Table S4.
Epitope residues for each mAb as defined based on the crystal structures described in this study.

	S811	S812	S813	S814	S815	S816	S817	S818	S819	S820	S821	S822	S823	S824	S825
**mAbs**	**K**	**P**	**S**	**K**	**R**	**S**	**F**	**I**	**E**	**D**	**L**	**L**	**F**	**N**	**K**
C13B8								■	■		■	■	■	■	■
C13C9					■			■	■		■	■	■	■	■
C77G12				■	■	■			■	■ *		■	■		
VN01H1					■	■			■	■		■	■	■	
VP12E7					■	■		■	■	■		■	■	■	■

- Shaded box indicates contact residues. The asterisk indicates that contacts are mediated by a water molecule.

Table S5.
Demographics of study participants

**Convalescent donor demographics (Fig. 1, A and D)**		
**Participants**		21
**Gender**	Female	8
	Male	13
**Age**	Median	59
	Range	34-96
**Days after PCR positive test**	Range	21-54

**Vaccinee donor demographics (Fig. 1, B to D)**		
**Participants**		22
**Gender**	Female	14
	Male	8
**Age**	Median	63
	Range	29-96
**Days after 2nd dose Pfizer/BioNTech BNT16b2**		14-29
**Naive**		10
**Pre-immune**		12

**Convalescent donor demographics (Fig. S4)**		
**Participants**		71
**Gender**	Female	47
	Male	24
**Age**	Median	43
	Range	21-63

References and Notes

1. A. N. Vlasova, A. Diaz, D. Damtie, L. Xiu, T.-H. Toh, J. S.-Y. Lee, L. J. Saif, G. C. Gray, Novel canine coronavirus isolated from a hospitalized patient with pneumonia in East Malaysia. *Clin. Infect. Dis.* **74**, 446–454 (2022). doi:10.1093/cid/ciab456 Medline

2. J. A. Lednicky, M. S. Tagliamonte, S. K. White, M. A. Elbadry, M. M. Alam, C. J. Stephenson, T. S. Bonny, J. C. Loeb, T. Telisma, S. Chavannes, D. A. Ostrov, C. Mavian, V. M. Beau De Rochars, M. Salemi, J. G. Morris Jr., Independent infections of porcine deltacoronavirus among Haitian children. *Nature* **600**, 133–137 (2021). doi:10.1038/s41586-021-04111-z Medline

3. M. A. Tortorici, A. C. Walls, A. Joshi, Y.-J. Park, R. T. Eguia, M. C. Miranda, E. Kepl, A. Dosey, T. Stevens-Ayers, M. J. Boeckh, A. Telenti, A. Lanzavecchia, N. P. King, D. Corti, J. D. Bloom, D. Veesler, Structure, receptor recognition, and antigenicity of the human coronavirus CCoV-HuPn-2018 spike glycoprotein. *Cell* **185**, 2279–2291.e17 (2022). doi:10.1016/j.cell.2022.05.019 Medline

4. A. C. Walls, Y.-J. Park, M. A. Tortorici, A. Wall, A. T. McGuire, D. Veesler, Structure, function, and antigenicity of the SARS-CoV-2 spike glycoprotein. *Cell* **181**, 281–292.e6 (2020). doi:10.1016/j.cell.2020.02.058 Medline

5. D. Wrapp, N. Wang, K. S. Corbett, J. A. Goldsmith, C.-L. Hsieh, O. Abiona, B. S. Graham, J. S. McLellan, Cryo-EM structure of the 2019-nCoV spike in the prefusion conformation. *Science* **367**, 1260–1263 (2020). doi:10.1126/science.abb2507 Medline

6. L. Piccoli, Y. J. Park, M. A. Tortorici, N. Czudnochowski, A. C. Walls, M. Beltramello, C. Silacci-Fregni, D. Pinto, L. E. Rosen, J. E. Bowen, O. J. Acton, S. Jaconi, B. Guarino, A. Minola, F. Zatta, N. Sprugasci, J. Bassi, A. Peter, A. De Marco, J. C. Nix, F. Mele, S. Jovic, B. F. Rodriguez, S. V. Gupta, F. Jin, G. Piumatti, G. Lo Presti, A. F. Pellanda, M. Biggiogero, M. Tarkowski, M. S. Pizzuto, E. Cameroni, C. Havenar-Daughton, M. Smithey, D. Hong, V. Lepori, E. Albanese, A. Ceschi, E. Bernasconi, L. Elzi, P. Ferrari, C. Garzoni, A. Riva, G. Snell, F. Sallusto, K. Fink, H. W. Virgin, A. Lanzavecchia, D. Corti, D. Veesler, Mapping neutralizing and immunodominant sites on the SARS-CoV-2 spike receptor-binding domain by structure-guided high-resolution serology. *Cell* **183**, 1024–1042.e21 (2020). doi:10.1016/j.cell.2020.09.037 Medline

7. A. J. Greaney, T. N. Starr, P. Gilchuk, S. J. Zost, E. Binshtein, A. N. Loes, S. K. Hilton, J. Huddleston, R. Eguia, K. H. D. Crawford, A. S. Dingens, R. S. Nargi, R. E. Sutton, N. Suryadevara, P. W. Rothlauf, Z. Liu, S. P. J. Whelan, R. H. Carnahan, J. E. Crowe Jr., J. D. Bloom, Complete mapping of mutations to the SARS-CoV-2 spike receptor-binding domain that escape antibody recognition. *Cell Host Microbe* **29**, 44–57.e9 (2021). doi:10.1016/j.chom.2020.11.007 Medline

8. A. Z. Wec, D. Wrapp, A. S. Herbert, D. P. Maurer, D. Haslwanter, M. Sakharkar, R. K. Jangra, M. E. Dieterle, A. Lilov, D. Huang, L. V. Tse, N. V. Johnson, C.-L. Hsieh, N. Wang, J. H. Nett, E. Champney, I. Burnina, M. Brown, S. Lin, M. Sinclair, C. Johnson, S. Pudi, R. Bortz III, A. S. Wirchnianski, E. Laudermilch, C. Florez, J. M. Fels, C. M. O'Brien, B. S. Graham, D. Nemazee, D. R. Burton, R. S. Baric, J. E. Voss, K. Chandran, J. M. Dye, J. S. McLellan, L. M. Walker, Broad neutralization of SARS-related viruses by human monoclonal antibodies. *Science* **369**, 731–736 (2020). doi:10.1126/science.abc7424 Medline

9. D. Pinto, Y. J. Park, M. Beltramello, A. C. Walls, M. A. Tortorici, S. Bianchi, S. Jaconi, K. Culap, F. Zatta, A. De Marco, A. Peter, B. Guarino, R. Spreafico, E. Cameroni, J. B. Case, R. E. Chen, C. Havenar-Daughton, G. Snell, A. Telenti, H. W. Virgin, A. Lanzavecchia, M. S. Diamond, K. Fink, D. Veesler, D. Corti, Cross-neutralization of SARS-CoV-2 by a human monoclonal SARS-CoV antibody. *Nature* **583**, 290–295 (2020). doi:10.1038/s41586-020-2349-y Medline

10. T. N. Starr, N. Czudnochowski, Z. Liu, F. Zatta, Y.-J. Park, A. Addetia, D. Pinto, M. Beltramello, P. Hernandez, A. J. Greaney, R. Marzi, W. G. Glass, I. Zhang, A. S. Dingens, J. E. Bowen, M. A. Tortorici, A. C. Walls, J. A. Wojcechowskyj, A. De Marco, L. E. Rosen, J. Zhou, M. Montiel-Ruiz, H. Kaiser, J. R. Dillen, H. Tucker, J. Bassi, C. Silacci-Fregni, M. P. Housley, J. di Iulio, G. Lombardo, M. Agostini, N. Sprugasci, K. Culap, S. Jaconi, M. Meury, E. Dellota Jr., R. Abdelnabi, S. C. Foo, E. Cameroni, S. Stumpf, T. I. Croll, J. C. Nix, C. Havenar-Daughton, L. Piccoli, F. Benigni, J. Neyts, A. Telenti, F. A. Lempp, M. S. Pizzuto, J. D. Chodera, C. M. Hebner, H. W. Virgin, S. P. J. Whelan, D. Veesler, D. Corti, J. D. Bloom, G. Snell, SARS-CoV-2 RBD antibodies that maximize breadth and resistance to escape. *Nature* **597**, 97–102 (2021). doi:10.1038/s41586-021-03807-6 Medline

11. C. A. Jette, A. A. Cohen, P. N. P. Gnanapragasam, F. Muecksch, Y. E. Lee, K. E. Huey-Tubman, F. Schmidt, T. Hatziioannou, P. D. Bieniasz, M. C. Nussenzweig, A. P. West Jr., J. R. Keeffe, P. J. Bjorkman, C. O. Barnes, Broad cross-reactivity across sarbecoviruses exhibited by a subset of COVID-19 donor-derived neutralizing antibodies. *Cell Rep.* **36**, 109760 (2021). doi:10.1016/j.celrep.2021.109760 Medline

12. M. A. Tortorici, N. Czudnochowski, T. N. Starr, R. Marzi, A. C. Walls, F. Zatta, J. E. Bowen, S. Jaconi, J. Di Iulio, Z. Wang, A. De Marco, S. K. Zepeda, D. Pinto, Z. Liu, M. Beltramello, I. Bartha, M. P. Housley, F. A. Lempp, L. E. Rosen, E. Dellota Jr., H. Kaiser, M. Montiel-Ruiz, J. Zhou, A. Addetia, B. Guarino, K. Culap, N. Sprugasci, C. Saliba, E. Vetti, I. Giacchetto-Sasselli, C. S. Fregni, R. Abdelnabi, S. C. Foo, C. Havenar-Daughton, M. A. Schmid, F. Benigni, E. Cameroni, J. Neyts, A. Telenti, H. W. Virgin, S. P. J. Whelan, G. Snell, J. D. Bloom, D. Corti, D. Veesler, M. S. Pizzuto, Broad sarbecovirus neutralization by a human monoclonal antibody. *Nature* **597**, 103–108 (2021). doi:10.1038/s41586-021-03817-4 Medline

13. Y.-J. Park, A. De Marco, T. N. Starr, Z. Liu, D. Pinto, A. C. Walls, F. Zatta, S. K. Zepeda, J. E. Bowen, K. R. Sprouse, A. Joshi, M. Giurdanella, B. Guarino, J. Noack, R. Abdelnabi, S. C. Foo, L. E. Rosen, F. A. Lempp, F. Benigni, G. Snell, J. Neyts, S. P. J. Whelan, H. W. Virgin, J. D. Bloom, D. Corti, M. S. Pizzuto, D. Veesler, Antibody-mediated broad sarbecovirus neutralization through ACE2 molecular mimicry. *Science* **375**, 449–454 (2022). doi:10.1126/science.abm8143 Medline

14. D. R. Martinez, A. Schäfer, S. Gobeil, D. Li, G. De la Cruz, R. Parks, X. Lu, M. Barr, V. Stalls, K. Janowska, E. Beaudoin, K. Manne, K. Mansouri, R. J. Edwards, K. Cronin, B. Yount, K. Anasti, S. A. Montgomery, J. Tang, H. Golding, S. Shen, T. Zhou, P. D. Kwong, B. S. Graham, J. R. Mascola, D. C. Montefiori, S. M. Alam, G. Sempowski, G. D. Sempowski, S. Khurana, K. Wiehe, K. O. Saunders, P. Acharya, B. F. Haynes, R. S. Baric, A broadly cross-reactive antibody neutralizes and protects against sarbecovirus challenge in mice. *Sci. Transl. Med.* **14**, eabj7125 (2022). doi:10.1126/scitranslmed.abj7125 Medline

15. D. Pinto, M. M. Sauer, N. Czudnochowski, J. S. Low, M. A. Tortorici, M. P. Housley, J. Noack, A. C. Walls, J. E. Bowen, B. Guarino, L. E. Rosen, J. di Iulio, J. Jerak, H. Kaiser, S. Islam, S. Jaconi, N. Sprugasci, K. Culap, R. Abdelnabi, C. Foo, L. Coelmont, I. Bartha, S. Bianchi, C. Silacci-Fregni, J. Bassi, R. Marzi, E. Vetti, A. Cassotta, A. Ceschi, P. Ferrari, P. E. Cippà, O. Giannini, S. Ceruti, C. Garzoni, A. Riva, F. Benigni, E. Cameroni, L. Piccoli, M. S. Pizzuto, M. Smithey, D. Hong, A. Telenti, F. A. Lempp, J. Neyts, C. Havenar-Daughton, A. Lanzavecchia, F. Sallusto, G. Snell, H. W. Virgin, M. Beltramello, D. Corti, D. Veesler, Broad betacoronavirus neutralization by a stem helix-specific human antibody. *Science* **373**, 1109–1116 (2021). doi:10.1126/science.abj3321 Medline

16. C. Wang, R. van Haperen, J. Gutiérrez-Álvarez, W. Li, N. M. A. Okba, I. Albulescu, I. Widjaja, B. van Dieren, R. Fernandez-Delgado, I. Sola, D. L. Hurdiss, O. Daramola, F. Grosveld, F. J. M. van Kuppeveld, B. L. Haagmans, L. Enjuanes, D. Drabek, B. J. Bosch, A conserved immunogenic and vulnerable site on the coronavirus spike protein delineated by cross-reactive monoclonal antibodies. *Nat. Commun.* **12**, 1715 (2021). doi:10.1038/s41467-021-21968-w Medline

17. G. Song, W. He, S. Callaghan, F. Anzanello, D. Huang, J. Ricketts, J. L. Torres, N. Beutler, L. Peng, S. Vargas, J. Cassell, M. Parren, L. Yang, C. Ignacio, D. M. Smith, J. E. Voss, D. Nemazee, A. B. Ward, T. Rogers, D. R. Burton, R. Andrabi, Cross-reactive serum and memory B-cell responses to spike protein in SARS-CoV-2 and endemic coronavirus infection. *Nat. Commun.* **12**, 2938 (2021). doi:10.1038/s41467-021-23074-3 Medline

18. M. M. Sauer, M. A. Tortorici, Y.-J. Park, A. C. Walls, L. Homad, O. J. Acton, J. E. Bowen, C. Wang, X. Xiong, W. de van der Schueren, J. Quispe, B. G. Hoffstrom, B.-J. Bosch, A. T. McGuire, D. Veesler, Structural basis for broad coronavirus neutralization. *Nat. Struct. Mol. Biol.* **28**, 478–486 (2021). doi:10.1038/s41594-021-00596-4 Medline

19. P. Zhou, M. Yuan, G. Song, N. Beutler, N. Shaabani, D. Huang, W. T. He, X. Zhu, S. Callaghan, P. Yong, F. Anzanello, L. Peng, J. Ricketts, M. Parren, E. Garcia, S. A. Rawlings, D. M. Smith, D. Nemazee, J. R. Teijaro, T. F. Rogers, I. A. Wilson, D. R. Burton, R. Andrabi, A human antibody reveals a conserved site on beta-coronavirus spike proteins and confers protection against SARS-CoV-2 infection. *Sci. Transl. Med.* **14**, eabi9215 (2022). doi:10.1126/scitranslmed.abi9215 Medline

20. P. Zhou, G. Song, W.-T. He, N. Beutler, L. v Tse, D. R. Martinez, A. Schäfer, F. Anzanello, P. Yong, L. Peng, K. Dueker, R. Musharrafieh, S. Callaghan, T. Capozzola, M. Yuan, H. Liu, O. Limbo, M. Parren, E. Garcia, S. A. Rawlings, D. M. Smith, D. Nemazee, J. G. Jardine, I. A. Wilson, Y. Safonova, T. F. Rogers, R. S. Baric, L. E. Gralinski, D. R. Burton, R. Andrabi, Broadly neutralizing anti-S2 antibodies protect against all three human betacoronaviruses that cause severe disease. bioRxiv 479488 [Preprint] (2022); https://doi.org/10.1101/2022.03.04.479488.

21. D. Corti, J. Voss, S. J. Gamblin, G. Codoni, A. Macagno, D. Jarrossay, S. G. Vachieri, D. Pinna, A. Minola, F. Vanzetta, C. Silacci, B. M. Fernandez-Rodriguez, G. Agatic, S. Bianchi, I. Giacchetto-Sasselli, L. Calder, F. Sallusto, P. Collins, L. F. Haire, N. Temperton, J. P. M. Langedijk, J. J. Skehel, A. Lanzavecchia, A neutralizing antibody selected from plasma cells that binds to group 1 and group 2 influenza A hemagglutinins. *Science* **333**, 850–856 (2011). doi:10.1126/science.1205669 Medline

22. M. M. Sajadi, A. Dashti, Z. Rikhtegaran Tehrani, W. D. Tolbert, M. S. Seaman, X. Ouyang, N. Gohain, M. Pazgier, D. Kim, G. Cavet, J. Yared, R. R. Redfield, G. K. Lewis, A. L. DeVico, Identification of near-pan-neutralizing antibodies against HIV-1 by deconvolution of plasma humoral responses. *Cell* **173**, 1783–1795.e14 (2018). doi:10.1016/j.cell.2018.03.061 Medline

23. H.-X. Liao, R. Lynch, T. Zhou, F. Gao, S. M. Alam, S. D. Boyd, A. Z. Fire, K. M. Roskin, C. A. Schramm, Z. Zhang, J. Zhu, L. Shapiro, J. C. Mullikin, S. Gnanakaran, P. Hraber, K. Wiehe, G. Kelsoe, G. Yang, S.-M. Xia, D. C. Montefiori, R. Parks, K. E. Lloyd, R. M. Scearce, K. A. Soderberg, M. Cohen, G. Kamanga, M. K. Louder, L. M. Tran, Y. Chen, F. Cai, S. Chen, S. Moquin, X. Du, M. G. Joyce, S. Srivatsan, B. Zhang, A. Zheng, G. M. Shaw, B. H. Hahn, T. B. Kepler, B. T. M. Korber, P. D. Kwong, J. R. Mascola, B. F. Haynes, Co-evolution of a broadly neutralizing HIV-1 antibody and founder virus. *Nature* **496**, 469–476 (2013). doi:10.1038/nature12053 Medline

24. J. F. Scheid, H. Mouquet, B. Ueberheide, R. Diskin, F. Klein, T. Y. K. Oliveira, J. Pietzsch, D. Fenyo, A. Abadir, K. Velinzon, A. Hurley, S. Myung, F. Boulad, P. Poignard, D. R. Burton, F. Pereyra, D. D. Ho, B. D. Walker, M. S. Seaman, P. J. Bjorkman, B. T. Chait, M. C. Nussenzweig, Sequence and structural convergence of broad and potent HIV antibodies that mimic CD4 binding. *Science* **333**, 1633–1637 (2011). doi:10.1126/science.1207227 Medline

25. J. Huang, G. Ofek, L. Laub, M. K. Louder, N. A. Doria-Rose, N. S. Longo, H. Imamichi, R. T. Bailer, B. Chakrabarti, S. K. Sharma, S. M. Alam, T. Wang, Y. Yang, B. Zhang, S. A. Migueles, R. Wyatt, B. F. Haynes, P. D. Kwong, J. R. Mascola, M. Connors, Broad and potent neutralization of HIV-1 by a gp41-specific human antibody. *Nature* **491**, 406–412 (2012). doi:10.1038/nature11544 Medline

26. C. Dreyfus, N. S. Laursen, T. Kwaks, D. Zuijdgeest, R. Khayat, D. C. Ekiert, J. H. Lee, Z. Metlagel, M. V. Bujny, M. Jongeneelen, R. van der Vlugt, M. Lamrani, H. J. W. M. Korse, E. Geelen, Ö. Sahin, M. Sieuwerts, J. P. J. Brakenhoff, R. Vogels, O. T. W. Li, L. L. M. Poon, M. Peiris, W. Koudstaal, A. B. Ward, I. A. Wilson, J. Goudsmit, R. H. E. Friesen, Highly conserved protective epitopes on influenza B viruses. *Science* **337**, 1343–1348 (2012). doi:10.1126/science.1222908 Medline

27. D. Sok, D. R. Burton, Recent progress in broadly neutralizing antibodies to HIV. *Nat. Immunol.* **19**, 1179–1188 (2018). doi:10.1038/s41590-018-0235-7 Medline

28. D. Corti, A. Lanzavecchia, Broadly neutralizing antiviral antibodies. *Annu. Rev. Immunol.* **31**, 705–742 (2013). doi:10.1146/annurev-immunol-032712-095916 Medline

29. D. Pinna, D. Corti, D. Jarrossay, F. Sallusto, A. Lanzavecchia, Clonal dissection of the human memory B-cell repertoire following infection and vaccination. *Eur. J. Immunol.* **39**, 1260–1270 (2009). doi:10.1002/eji.200839129 Medline

30. A. C. Walls, K. R. Sprouse, J. E. Bowen, A. Joshi, N. Franko, M. J. Navarro, C. Stewart, E. Cameroni, M. McCallum, E. A. Goecker, E. J. Degli-Angeli, J. Logue, A. Greninger, D. Corti, H. Y. Chu, D. Veesler, SARS-CoV-2 breakthrough infections elicit potent, broad, and durable neutralizing antibody responses. *Cell* **185**, 872–880.e3 (2022). doi:10.1016/j.cell.2022.01.011 Medline

31. E. M. Anderson, E. C. Goodwin, A. Verma, C. P. Arevalo, M. J. Bolton, M. E. Weirick, S. Gouma, C. M. McAllister, S. R. Christensen, J. Weaver, P. Hicks, T. B. Manzoni, O.

Oniyide, H. Ramage, D. Mathew, A. E. Baxter, D. A. Oldridge, A. R. Greenplate, J. E. Wu, C. Alanio, K. D'Andrea, O. Kuthuru, J. Dougherty, A. Pattekar, J. Kim, N. Han, S. A. Apostolidis, A. C. Huang, L. A. Vella, L. Kuri-Cervantes, M. B. Pampena, M. R. Betts, E. J. Wherry, N. J. Meyer, S. Cherry, P. Bates, D. J. Rader, S. E. Hensley; UPenn COVID Processing Unit, Seasonal human coronavirus antibodies are boosted upon SARS-CoV-2 infection but not associated with protection. *Cell* **184**, 1858–1864.e10 (2021). doi:10.1016/j.cell.2021.02.010 Medline

32. M. Hoffmann, H. Kleine-Weber, S. Schroeder, N. Krüger, T. Herrler, S. Erichsen, T. S. Schiergens, G. Herrler, N. H. Wu, A. Nitsche, M. A. Müller, C. Drosten, S. Pöhlmann, SARS-CoV-2 cell entry depends on ACE2 and TMPRSS2 and is blocked by a clinically proven protease inhibitor. *Cell* **181**, 271–280.e8 (2020). doi:10.1016/j.cell.2020.02.052 Medline

33. C.-L. Hsieh, J. A. Goldsmith, J. M. Schaub, A. M. DiVenere, H.-C. Kuo, K. Javanmardi, K. C. Le, D. Wrapp, A. G. Lee, Y. Liu, C.-W. Chou, P. O. Byrne, C. K. Hjorth, N. V. Johnson, J. Ludes-Meyers, A. W. Nguyen, J. Park, N. Wang, D. Amengor, J. J. Lavinder, G. C. Ippolito, J. A. Maynard, I. J. Finkelstein, J. S. McLellan, Structure-based design of prefusion-stabilized SARS-CoV-2 spikes. *Science* **369**, 1501–1505 (2020). doi:10.1126/science.abd0826 Medline

34. E. Shrock, E. Fujimura, T. Kula, R. T. Timms, I.-H. Lee, Y. Leng, M. L. Robinson, B. M. Sie, M. Z. Li, Y. Chen, J. Logue, A. Zuiani, D. McCulloch, F. J. N. Lelis, S. Henson, D. R. Monaco, M. Travers, S. Habibi, W. A. Clarke, P. Caturegli, O. Laeyendecker, A. Piechocka-Trocha, J. Z. Li, A. Khatri, H. Y. Chu, A.-C. Villani, K. Kays, M. B. Goldberg, N. Hacohen, M. R. Filbin, X. G. Yu, B. D. Walker, D. R. Wesemann, H. B. Larman, J. A. Lederer, S. J. Elledge, Viral epitope profiling of COVID-19 patients reveals cross-reactivity and correlates of severity. *Science* **370**, eabd4250 (2020). doi:10.1126/science.abd4250 Medline

35. K. W. Ng, N. Faulkner, G. H. Cornish, A. Rosa, R. Harvey, S. Hussain, R. Ulferts, C. Earl, A. G. Wrobel, D. J. Benton, C. Roustan, W. Bolland, R. Thompson, A. Agua-Doce, P. Hobson, J. Heaney, H. Rickman, S. Paraskevopoulou, C. F. Houlihan, K. Thomson, E. Sanchez, G. Y. Shin, M. J. Spyer, D. Joshi, N. O'Reilly, P. A. Walker, S. Kjaer, A. Riddell, C. Moore, B. R. Jebson, M. Wilkinson, L. R. Marshall, E. C. Rosser, A. Radziszewska, H. Peckham, C. Ciurtin, L. R. Wedderburn, R. Beale, C. Swanton, S. Gandhi, B. Stockinger, J. McCauley, S. J. Gamblin, L. E. McCoy, P. Cherepanov, E. Nastouli, G. Kassiotis, Preexisting and de novo humoral immunity to SARS-CoV-2 in humans. *Science* **370**, 1339–1343 (2020). doi:10.1126/science.abe1107 Medline

36. C. M. Poh, G. Carissimo, B. Wang, S. N. Amrun, C. Y.-P. Lee, R. S.-L. Chee, S.-W. Fong, N. K.-W. Yeo, W.-H. Lee, A. Torres-Ruesta, Y.-S. Leo, M. I.-C. Chen, S.-Y. Tan, L. Y. A. Chai, S. Kalimuddin, S. S. G. Kheng, S.-Y. Thien, B. E. Young, D. C. Lye, B. J. Hanson, C.-I. Wang, L. Renia, L. F. P. Ng, Two linear epitopes on the SARS-CoV-2 spike protein that elicit neutralising antibodies in COVID-19 patients. *Nat. Commun.* **11**, 2806 (2020). doi:10.1038/s41467-020-16638-2 Medline

37. C. Daniel, R. Anderson, M. J. Buchmeier, J. O. Fleming, W. J. Spaan, H. Wege, P. J. Talbot, Identification of an immunodominant linear neutralization domain on the S2 portion of the murine coronavirus spike glycoprotein and evidence that it forms part of complex

tridimensional structure. *J. Virol.* **67**, 1185–1194 (1993). doi:10.1128/jvi.67.3.1185-1194.1993 Medline

38. H. Zhang, G. Wang, J. Li, Y. Nie, X. Shi, G. Lian, W. Wang, X. Yin, Y. Zhao, X. Qu, M. Ding, H. Deng, Identification of an antigenic determinant on the S2 domain of the severe acute respiratory syndrome coronavirus spike glycoprotein capable of inducing neutralizing antibodies. *J. Virol.* **78**, 6938–6945 (2004). doi:10.1128/JVI.78.13.6938-6945.2004 Medline

39. A. C. Walls, M. A. Tortorici, B. J. Bosch, B. Frenz, P. J. M. Rottier, F. DiMaio, F. A. Rey, D. Veesler, Cryo-electron microscopy structure of a coronavirus spike glycoprotein trimer. *Nature* **531**, 114–117 (2016). doi:10.1038/nature16988 Medline

40. Q. Xiong, L. Cao, C. Ma, C. Liu, J. Si, P. Liu, M. Gu, C. Wang, L. Shi, F. Tong, M. Huang, J. Li, C. Zhao, C. Shen, Y. Chen, H. Zhao, K. Lan, X. Wang, H. Yan, Close relatives of MERS-CoV in bats use ACE2 as their functional receptors. bioRxiv 477490 [Preprint] (2022); https://doi.org/10.1101/2022.01.24.477490.

41. F. A. Lempp, L. B. Soriaga, M. Montiel-Ruiz, F. Benigni, J. Noack, Y.-J. Park, S. Bianchi, A. C. Walls, J. E. Bowen, J. Zhou, H. Kaiser, A. Joshi, M. Agostini, M. Meury, E. Dellota Jr., S. Jaconi, E. Cameroni, J. Martinez-Picado, J. Vergara-Alert, N. Izquierdo-Useros, H. W. Virgin, A. Lanzavecchia, D. Veesler, L. A. Purcell, A. Telenti, D. Corti, Lectins enhance SARS-CoV-2 infection and influence neutralizing antibodies. *Nature* **598**, 342–347 (2021). doi:10.1038/s41586-021-03925-1 Medline

42. H. V. Dang, Y.-P. Chan, Y.-J. Park, J. Snijder, S. C. Da Silva, B. Vu, L. Yan, Y.-R. Feng, B. Rockx, T. W. Geisbert, C. E. Mire, C. C. Broder, D. Veesler, An antibody against the F glycoprotein inhibits Nipah and Hendra virus infections. *Nat. Struct. Mol. Biol.* **26**, 980–987 (2019). doi:10.1038/s41594-019-0308-9 Medline

43. A. C. Walls, M. A. Tortorici, J. Snijder, X. Xiong, B.-J. Bosch, F. A. Rey, D. Veesler, Tectonic conformational changes of a coronavirus spike glycoprotein promote membrane fusion. *Proc. Natl. Acad. Sci. U.S.A.* **114**, 11157–11162 (2017). doi:10.1073/pnas.1708727114 Medline

44. B. A. Johnson, X. Xie, A. L. Bailey, B. Kalveram, K. G. Lokugamage, A. Muruato, J. Zou, X. Zhang, T. Juelich, J. K. Smith, L. Zhang, N. Bopp, C. Schindewolf, M. Vu, A. Vanderheiden, E. S. Winkler, D. Swetnam, J. A. Plante, P. Aguilar, K. S. Plante, V. Popov, B. Lee, S. C. Weaver, M. S. Suthar, A. L. Routh, P. Ren, Z. Ku, Z. An, K. Debbink, M. S. Diamond, P.-Y. Shi, A. N. Freiberg, V. D. Menachery, Loss of furin cleavage site attenuates SARS-CoV-2 pathogenesis. *Nature* **591**, 293–299 (2021). doi:10.1038/s41586-021-03237-4 Medline

45. M. Hoffmann, H. Kleine-Weber, S. Pöhlmann, A multibasic cleavage site in the spike protein of SARS-CoV-2 is essential for infection of human lung cells. *Mol. Cell* **78**, 779–784.e5 (2020). doi:10.1016/j.molcel.2020.04.022 Medline

46. S. M.-C. Gobeil, R. Henderson, V. Stalls, K. Janowska, X. Huang, A. May, M. Speakman, E. Beaudoin, K. Manne, D. Li, R. Parks, M. Barr, M. Deyton, M. Martin, K. Mansouri, R. J. Edwards, A. Eaton, D. C. Montefiori, G. D. Sempowski, K. O. Saunders, K. Wiehe, W. Williams, B. Korber, B. F. Haynes, P. Acharya, Structural diversity of the SARS-CoV-2 Omicron spike. *Mol. Cell* **82**, 2050–2068.e6 (2022). doi:10.1016/j.molcel.2022.03.028 Medline

47. W. Li, M. J. Moore, N. Vasilieva, J. Sui, S. K. Wong, M. A. Berne, M. Somasundaran, J. L. Sullivan, K. Luzuriaga, T. C. Greenough, H. Choe, M. Farzan, Angiotensin-converting enzyme 2 is a functional receptor for the SARS coronavirus. *Nature* **426**, 450–454 (2003). doi:10.1038/nature02145 Medline

48. V. S. Raj, H. Mou, S. L. Smits, D. H. W. Dekkers, M. A. Müller, R. Dijkman, D. Muth, J. A. A. Demmers, A. Zaki, R. A. M. Fouchier, V. Thiel, C. Drosten, P. J. M. Rottier, A. D. M. E. Osterhaus, B. J. Bosch, B. L. Haagmans, Dipeptidyl peptidase 4 is a functional receptor for the emerging human coronavirus-EMC. *Nature* **495**, 251–254 (2013). doi:10.1038/nature12005 Medline

49. H. Hofmann, K. Pyrc, L. van der Hoek, M. Geier, B. Berkhout, S. Pöhlmann, Human coronavirus NL63 employs the severe acute respiratory syndrome coronavirus receptor for cellular entry. *Proc. Natl. Acad. Sci. U.S.A.* **102**, 7988–7993 (2005). doi:10.1073/pnas.0409465102 Medline

50. C. L. Yeager, R. A. Ashmun, R. K. Williams, C. B. Cardellichio, L. H. Shapiro, A. T. Look, K. V. Holmes, Human aminopeptidase N is a receptor for human coronavirus 229E. *Nature* **357**, 420–422 (1992). doi:10.1038/357420a0 Medline

51. M. A. Tortorici, M. Beltramello, F. A. Lempp, D. Pinto, H. V. Dang, L. E. Rosen, M. McCallum, J. Bowen, A. Minola, S. Jaconi, F. Zatta, A. De Marco, B. Guarino, S. Bianchi, E. J. Lauron, H. Tucker, J. Zhou, A. Peter, C. Havenar-Daughton, J. A. Wojcechowskyj, J. B. Case, R. E. Chen, H. Kaiser, M. Montiel-Ruiz, M. Meury, N. Czudnochowski, R. Spreafico, J. Dillen, C. Ng, N. Sprugasci, K. Culap, F. Benigni, R. Abdelnabi, S. C. Foo, M. A. Schmid, E. Cameroni, A. Riva, A. Gabrieli, M. Galli, M. S. Pizzuto, J. Neyts, M. S. Diamond, H. W. Virgin, G. Snell, D. Corti, K. Fink, D. Veesler, Ultrapotent human antibodies protect against SARS-CoV-2 challenge via multiple mechanisms. *Science* **370**, 950–957 (2020). doi:10.1126/science.abe3354 Medline

52. A. C. Walls, X. Xiong, Y. J. Park, M. A. Tortorici, J. Snijder, J. Quispe, E. Cameroni, R. Gopal, M. Dai, A. Lanzavecchia, M. Zambon, F. A. Rey, D. Corti, D. Veesler, Unexpected receptor functional mimicry elucidates activation of coronavirus fusion. *Cell* **176**, 1026–1039.e15 (2019). doi:10.1016/j.cell.2018.12.028 Medline

53. D. R. Burton, Advancing an HIV vaccine; advancing vaccinology. *Nat. Rev. Immunol.* **19**, 77–78 (2019). doi:10.1038/s41577-018-0103-6 Medline

54. M. Bonsignori, H.-X. Liao, F. Gao, W. B. Williams, S. M. Alam, D. C. Montefiori, B. F. Haynes, Antibody-virus co-evolution in HIV infection: Paths for HIV vaccine development. *Immunol. Rev.* **275**, 145–160 (2017). doi:10.1111/imr.12509 Medline

55. J.-E. Park, K. Li, A. Barlan, A. R. Fehr, S. Perlman, P. B. McCray Jr., T. Gallagher, Proteolytic processing of Middle East respiratory syndrome coronavirus spikes expands virus tropism. *Proc. Natl. Acad. Sci. U.S.A.* **113**, 12262–12267 (2016). doi:10.1073/pnas.1608147113 Medline

56. J. K. Millet, G. R. Whittaker, Host cell entry of Middle East respiratory syndrome coronavirus after two-step, furin-mediated activation of the spike protein. *Proc. Natl. Acad. Sci. U.S.A.* **111**, 15214–15219 (2014). doi:10.1073/pnas.1407087111 Medline

57. P. V. Raghuvamsi, N. K. Tulsian, F. Samsudin, X. Qian, K. Purushotorman, G. Yue, M. M. Kozma, W. Y. Hwa, J. Lescar, P. J. Bond, P. A. MacAry, G. S. Anand, SARS-CoV-2 S

protein:ACE2 interaction reveals novel allosteric targets. *eLife* **10**, e63646 (2021). doi:10.7554/eLife.63646 Medline

58. S. Yu, X. Zheng, B. Zhou, J. Li, M. Chen, R. Deng, G. Wong, D. Lavillette, G. Meng, SARS-CoV-2 spike engagement of ACE2 primes S2′ site cleavage and fusion initiation. *Proc. Natl. Acad. Sci. U.S.A.* **119**, e2111199119 (2022). doi:10.1073/pnas.2111199119 Medline

59. E. Qing, P. Li, L. Cooper, S. Schulz, H.-M. Jäck, L. Rong, S. Perlman, T. Gallagher, Inter-domain communication in SARS-CoV-2 spike proteins controls protease-triggered cell entry. *Cell Rep.* **39**, 110786 (2022). doi:10.1016/j.celrep.2022.110786 Medline

60. C. Cheng, K. Xu, R. Kong, G. Y. Chuang, A. R. Corrigan, H. Geng, K. R. Hill, A. J. Jafari, S. O'Dell, L. Ou, R. Rawi, A. P. Rowshan, E. K. Sarfo, M. Sastry, K. O. Saunders, S. D. Schmidt, S. Wang, W. Wu, B. Zhang, N. A. Doria-Rose, B. F. Haynes, D. G. Scorpio, L. Shapiro, J. R. Mascola, P. D. Kwong, Consistent elicitation of cross-clade HIV-neutralizing responses achieved in guinea pigs after fusion peptide priming by repetitive envelope trimer boosting. *PLOS ONE* **14**, e0215163 (2019). doi:10.1371/journal.pone.0215163 Medline

61. K. Xu, P. Acharya, R. Kong, C. Cheng, G. Y. Chuang, K. Liu, M. K. Louder, S. O'Dell, R. Rawi, M. Sastry, C. H. Shen, B. Zhang, T. Zhou, M. Asokan, R. T. Bailer, M. Chambers, X. Chen, C. W. Choi, V. P. Dandey, N. A. Doria-Rose, A. Druz, E. T. Eng, S. K. Farney, K. E. Foulds, H. Geng, I. S. Georgiev, J. Gorman, K. R. Hill, A. J. Jafari, Y. D. Kwon, Y. T. Lai, T. Lemmin, K. McKee, T. Y. Ohr, L. Ou, D. Peng, A. P. Rowshan, Z. Sheng, J. P. Todd, Y. Tsybovsky, E. G. Viox, Y. Wang, H. Wei, Y. Yang, A. F. Zhou, R. Chen, L. Yang, D. G. Scorpio, A. B. McDermott, L. Shapiro, B. Carragher, C. S. Potter, J. R. Mascola, P. D. Kwong, Epitope-based vaccine design yields fusion peptide-directed antibodies that neutralize diverse strains of HIV-1. *Nat. Med.* **24**, 857–867 (2018). doi:10.1038/s41591-018-0042-6 Medline

62. R. Kong, K. Xu, T. Zhou, P. Acharya, T. Lemmin, K. Liu, G. Ozorowski, C. Soto, J. D. Taft, R. T. Bailer, E. M. Cale, L. Chen, C. W. Choi, G.-Y. Chuang, N. A. Doria-Rose, A. Druz, I. S. Georgiev, J. Gorman, J. Huang, M. G. Joyce, M. K. Louder, X. Ma, K. McKee, S. O'Dell, M. Pancera, Y. Yang, S. C. Blanchard, W. Mothes, D. R. Burton, W. C. Koff, M. Connors, A. B. Ward, P. D. Kwong, J. R. Mascola, Fusion peptide of HIV-1 as a site of vulnerability to neutralizing antibody. *Science* **352**, 828–833 (2016). doi:10.1126/science.aae0474 Medline

63. L. Ou, W.-P. Kong, G.-Y. Chuang, M. Ghosh, K. Gulla, S. O'Dell, J. Varriale, N. Barefoot, A. Changela, C. W. Chao, C. Cheng, A. Druz, R. Kong, K. McKee, R. Rawi, E. K. Sarfo, A. Schön, A. Shaddeau, Y. Tsybovsky, R. Verardi, S. Wang, T. G. Wanninger, K. Xu, G. J. Yang, B. Zhang, Y. Zhang, T. Zhou, F. J. Arnold, N. A. Doria-Rose, Q. P. Lei, E. T. Ryan, W. F. Vann, J. R. Mascola, P. D. Kwong; VRC Production Program, Preclinical development of a fusion peptide conjugate as an HIV vaccine immunogen. *Sci. Rep.* **10**, 3032 (2020). doi:10.1038/s41598-020-59711-y Medline

64. R. Nachbagauer, J. Feser, A. Naficy, D. I. Bernstein, J. Guptill, E. B. Walter, F. Berlanda-Scorza, D. Stadlbauer, P. C. Wilson, T. Aydillo, M. A. Behzadi, D. Bhavsar, C. Bliss, C. Capuano, J. M. Carreño, V. Chromikova, C. Claeys, L. Coughlan, A. W. Freyn, C. Gast, A. Javier, K. Jiang, C. Mariottini, M. McMahon, M. McNeal, A. Solórzano, S.

Strohmeier, W. Sun, M. Van der Wielen, B. L. Innis, A. García-Sastre, P. Palese, F. Krammer, A chimeric hemagglutinin-based universal influenza virus vaccine approach induces broad and long-lasting immunity in a randomized, placebo-controlled phase I trial. *Nat. Med.* **27**, 106–114 (2021). doi:10.1038/s41591-020-1118-7 Medline

65. H. M. Yassine, J. C. Boyington, P. M. McTamney, C. J. Wei, M. Kanekiyo, W. P. Kong, J. R. Gallagher, L. Wang, Y. Zhang, M. G. Joyce, D. Lingwood, S. M. Moin, H. Andersen, Y. Okuno, S. S. Rao, A. K. Harris, P. D. Kwong, J. R. Mascola, G. J. Nabel, B. S. Graham, Hemagglutinin-stem nanoparticles generate heterosubtypic influenza protection. *Nat. Med.* **21**, 1065–1070 (2015). doi:10.1038/nm.3927 Medline

66. A. Impagliazzo, F. Milder, H. Kuipers, M. V. Wagner, X. Zhu, R. M. B. Hoffman, R. van Meersbergen, J. Huizingh, P. Wanningen, J. Verspuij, M. de Man, Z. Ding, A. Apetri, B. Kükrer, E. Sneekes-Vriese, D. Tomkiewicz, N. S. Laursen, P. S. Lee, A. Zakrzewska, L. Dekking, J. Tolboom, L. Tettero, S. van Meerten, W. Yu, W. Koudstaal, J. Goudsmit, A. B. Ward, W. Meijberg, I. A. Wilson, K. Radošević, A stable trimeric influenza hemagglutinin stem as a broadly protective immunogen. *Science* **349**, 1301–1306 (2015). doi:10.1126/science.aac7263 Medline

67. S. Belouzard, V. C. Chu, G. R. Whittaker, Activation of the SARS coronavirus spike protein via sequential proteolytic cleavage at two distinct sites. *Proc. Natl. Acad. Sci. U.S.A.* **106**, 5871–5876 (2009). doi:10.1073/pnas.0809524106 Medline

68. I. G. Madu, S. L. Roth, S. Belouzard, G. R. Whittaker, Characterization of a highly conserved domain within the severe acute respiratory syndrome coronavirus spike protein S2 domain with characteristics of a viral fusion peptide. *J. Virol.* **83**, 7411–7421 (2009). doi:10.1128/JVI.00079-09 Medline

69. J. S. Low, D. Vaqueirinho, F. Mele, M. Foglierini, J. Jerak, M. Perotti, D. Jarrossay, S. Jovic, L. Perez, R. Cacciatore, T. Terrot, A. F. Pellanda, M. Biggiogero, C. Garzoni, P. Ferrari, A. Ceschi, A. Lanzavecchia, F. Sallusto, A. Cassotta, Clonal analysis of immunodominance and cross-reactivity of the CD4 T cell response to SARS-CoV-2. *Science* **372**, 1336–1341 (2021). doi:10.1126/science.abg8985 Medline

70. M. N. Prichard, C. Shipman Jr., A three-dimensional model to analyze drug-drug interactions. *Antiviral Res.* **14**, 181–205 (1990). doi:10.1016/0166-3542(90)90001-N Medline

71. T. Tiller, E. Meffre, S. Yurasov, M. Tsuiji, M. C. Nussenzweig, H. Wardemann, Efficient generation of monoclonal antibodies from single human B cells by single cell RT-PCR and expression vector cloning. *J. Immunol. Methods* **329**, 112–124 (2008). doi:10.1016/j.jim.2007.09.017 Medline

72. J. E. Bowen, A. C. Walls, A. Joshi, K. R. Sprouse, C. Stewart, M. A. Tortorici, N. M. Franko, J. K. Logue, I. G. Mazzitelli, S. W. Tiles, K. Ahmed, A. Shariq, G. Snell, N. T. Iqbal, J. Geffner, A. Bandera, A. Gori, R. Grifantini, H. Y. Chu, W. C. van Voorhis, D. Corti, D. Veesler, SARS-CoV-2 spike conformation determines plasma neutralizing activity. bioRxiv 473391 [Preprint] (2021); https://doi.org/10.1101/2021.12.19.473391.

73. E. Olmedillas, C. J. Mann, W. Peng, Y.-T. Wang, R. D. Avalos, D. Bedinger, K. Valentine, N. Shafee, S. L. Schendel, M. Yuan, G. Lang, R. Rouet, D. Christ, W. Jiang, I. A. Wilson, T. Germann, S. Shresta, J. Snijder, E. O. Saphire, Structure-based design of a highly stable, covalently-linked SARS-CoV-2 spike trimer with improved structural

properties and immunogenicity. bioRxiv 441046 [Preprint] (2021); https://doi.org/10.1101/2021.05.06.441046.

74. J. Pallesen, N. Wang, K. S. Corbett, D. Wrapp, R. N. Kirchdoerfer, H. L. Turner, C. A. Cottrell, M. M. Becker, L. Wang, W. Shi, W.-P. Kong, E. L. Andres, A. N. Kettenbach, M. R. Denison, J. D. Chappell, B. S. Graham, A. B. Ward, J. S. McLellan, Immunogenicity and structures of a rationally designed prefusion MERS-CoV spike antigen. *Proc. Natl. Acad. Sci. U.S.A.* **114**, E7348–E7357 (2017). doi:10.1073/pnas.1707304114 Medline

75. H. X. Liao, M. C. Levesque, A. Nagel, A. Dixon, R. Zhang, E. Walter, R. Parks, J. Whitesides, D. J. Marshall, K. K. Hwang, Y. Yang, X. Chen, F. Gao, S. Munshaw, T. B. Kepler, T. Denny, M. A. Moody, B. F. Haynes, High-throughput isolation of immunoglobulin genes from single human B cells and expression as monoclonal antibodies. *J. Virol. Methods* **158**, 171–179 (2009). doi:10.1016/j.jviromet.2009.02.014 Medline

76. J. Ye, N. Ma, T. L. Madden, J. M. Ostell, IgBLAST: An immunoglobulin variable domain sequence analysis tool. *Nucleic Acids Res.* **41**, W34–W40 (2013). doi:10.1093/nar/gkt382 Medline

77. M.-P. Lefranc, G. Lefranc, *The Immunoglobulin FactsBook* (Academic, 2001).

78. K. B. Hoehn, J. A. Vander Heiden, J. Q. Zhou, G. Lunter, O. G. Pybus, S. H. Kleinstein, Repertoire-wide phylogenetic models of B cell molecular evolution reveal evolutionary signatures of aging and vaccination. *Proc. Natl. Acad. Sci. U.S.A.* **116**, 22664–22672 (2019). doi:10.1073/pnas.1906020116 Medline

79. C. Suloway, J. Pulokas, D. Fellmann, A. Cheng, F. Guerra, J. Quispe, S. Stagg, C. S. Potter, B. Carragher, Automated molecular microscopy: The new Leginon system. *J. Struct. Biol.* **151**, 41–60 (2005). doi:10.1016/j.jsb.2005.03.010 Medline

80. K. H. D. Crawford, R. Eguia, A. S. Dingens, A. N. Loes, K. D. Malone, C. R. Wolf, H. Y. Chu, M. A. Tortorici, D. Veesler, M. Murphy, D. Pettie, N. P. King, A. B. Balazs, J. D. Bloom, Protocol and reagents for pseudotyping lentiviral particles with SARS-CoV-2 spike protein for neutralization assays. *Viruses* **12**, 513 (2020). doi:10.3390/v12050513 Medline

81. Y. Kaname, H. Tani, C. Kataoka, M. Shiokawa, S. Taguwa, T. Abe, K. Moriishi, T. Kinoshita, Y. Matsuura, Acquisition of complement resistance through incorporation of CD55/decay-accelerating factor into viral particles bearing baculovirus GP64. *J. Virol.* **84**, 3210–3219 (2010). doi:10.1128/JVI.02519-09 Medline

82. W. Kabsch, XDS. *Acta Crystallogr. D Biol. Crystallogr.* **66**, 125–132 (2010). doi:10.1107/S0907444909047337 Medline

83. T. G. G. Battye, L. Kontogiannis, O. Johnson, H. R. Powell, A. G. W. Leslie, iMOSFLM: A new graphical interface for diffraction-image processing with MOSFLM. *Acta Crystallogr. D Biol. Crystallogr.* **67**, 271–281 (2011). doi:10.1107/S0907444910048675 Medline

84. P. R. Evans, G. N. Murshudov, How good are my data and what is the resolution? *Acta Crystallogr. D Biol. Crystallogr.* **69**, 1204–1214 (2013). doi:10.1107/S0907444913000061 Medline

85. A. J. McCoy, R. W. Grosse-Kunstleve, P. D. Adams, M. D. Winn, L. C. Storoni, R. J. Read, Phaser crystallographic software. *J. Appl. Crystallogr.* **40**, 658–674 (2007). doi:10.1107/S0021889807021206 Medline

86. P. Emsley, B. Lohkamp, W. G. Scott, K. Cowtan, Features and development of Coot. *Acta Crystallogr. D Biol. Crystallogr.* **66**, 486–501 (2010). doi:10.1107/S0907444910007493 Medline

87. D. Liebschner, P. V. Afonine, M. L. Baker, G. Bunkóczi, V. B. Chen, T. I. Croll, B. Hintze, L. W. Hung, S. Jain, A. J. McCoy, N. W. Moriarty, R. D. Oeffner, B. K. Poon, M. G. Prisant, R. J. Read, J. S. Richardson, D. C. Richardson, M. D. Sammito, O. V. Sobolev, D. H. Stockwell, T. C. Terwilliger, A. G. Urzhumtsev, L. L. Videau, C. J. Williams, P. D. Adams, Macromolecular structure determination using X-rays, neutrons and electrons: Recent developments in Phenix. *Acta Crystallogr. D Struct. Biol.* **75**, 861–877 (2019). doi:10.1107/S2059798319011471 Medline

88. E. Blanc, P. Roversi, C. Vonrhein, C. Flensburg, S. M. Lea, G. Bricogne, Refinement of severely incomplete structures with maximum likelihood in BUSTER-TNT. *Acta Crystallogr. D Biol. Crystallogr.* **60**, 2210–2221 (2004). doi:10.1107/S0907444904016427 Medline

89. T. Giroglou, J. Cinatl Jr., H. Rabenau, C. Drosten, H. Schwalbe, H. W. Doerr, D. von Laer, Retroviral vectors pseudotyped with severe acute respiratory syndrome coronavirus S protein. *J. Virol.* **78**, 9007–9015 (2004). doi:10.1128/JVI.78.17.9007-9015.2004 Medline

90. R. Boudewijns, H. J. Thibaut, S. J. F. Kaptein, R. Li, V. Vergote, L. Seldeslachts, J. Van Weyenbergh, C. De Keyzer, L. Bervoets, S. Sharma, L. Liesenborghs, J. Ma, S. Jansen, D. Van Looveren, T. Vercruysse, X. Wang, D. Jochmans, E. Martens, K. Roose, D. De Vlieger, B. Schepens, T. Van Buyten, S. Jacobs, Y. Liu, J. Martí-Carreras, B. Vanmechelen, T. Wawina-Bokalanga, L. Delang, J. Rocha-Pereira, L. Coelmont, W. Chiu, P. Leyssen, E. Heylen, D. Schols, L. Wang, L. Close, J. Matthijnssens, M. Van Ranst, V. Compernolle, G. Schramm, K. Van Laere, X. Saelens, N. Callewaert, G. Opdenakker, P. Maes, B. Weynand, C. Cawthorne, G. Vande Velde, Z. Wang, J. Neyts, K. Dallmeier, STAT2 signaling restricts viral dissemination but drives severe pneumonia in SARS-CoV-2 infected hamsters. *Nat. Commun.* **11**, 5838 (2020). doi:10.1038/s41467-020-19684-y Medline

91. L. Sanchez-Felipe, T. Vercruysse, S. Sharma, J. Ma, V. Lemmens, D. Van Looveren, M. P. Arkalagud Javarappa, R. Boudewijns, B. Malengier-Devlies, L. Liesenborghs, S. J. F. Kaptein, C. De Keyzer, L. Bervoets, S. Debaveye, M. Rasulova, L. Seldeslachts, L.-H. Li, S. Jansen, M. B. Yakass, B. E. Verstrepen, K. P. Böszörményi, G. Kiemenyi-Kayere, N. van Driel, O. Quaye, X. Zhang, S. Ter Horst, N. Mishra, W. Deboutte, J. Matthijnssens, L. Coelmont, C. Vandermeulen, E. Heylen, V. Vergote, D. Schols, Z. Wang, W. Bogers, T. Kuiken, E. Verschoor, C. Cawthorne, K. Van Laere, G. Opdenakker, G. Vande Velde, B. Weynand, D. E. Teuwen, P. Matthys, J. Neyts, H. Jan Thibaut, K. Dallmeier, A single-dose live-attenuated YF17D-vectored SARS-CoV-2 vaccine candidate. *Nature* **590**, 320–325 (2021). doi:10.1038/s41586-020-3035-9 Medline

92. S. J. F. Kaptein, S. Jacobs, L. Langendries, L. Seldeslachts, S. Ter Horst, L. Liesenborghs, B. Hens, V. Vergote, E. Heylen, K. Barthelemy, E. Maas, C. De Keyzer, L. Bervoets, J.

Rymenants, T. Van Buyten, X. Zhang, R. Abdelnabi, J. Pang, R. Williams, H. J. Thibaut, K. Dallmeier, R. Boudewijns, J. Wouters, P. Augustijns, N. Verougstraete, C. Cawthorne, J. Breuer, C. Solas, B. Weynand, P. Annaert, I. Spriet, G. Vande Velde, J. Neyts, J. Rocha-Pereira, L. Delang, Favipiravir at high doses has potent antiviral activity in SARS-CoV-2-infected hamsters, whereas hydroxychloroquine lacks activity. *Proc. Natl. Acad. Sci. U.S.A.* **117**, 26955–26965 (2020). doi:10.1073/pnas.2014441117 Medline

93. R. Abdelnabi, C. S. Foo, D. Jochmans, L. Vangeel, S. De Jonghe, P. Augustijns, R. Mols, B. Weynand, T. Wattanakul, R. M. Hoglund, J. Tarning, C. E. Mowbray, P. Sjö, F. Escudié, I. Scandale, E. Chatelain, J. Neyts, The oral protease inhibitor (PF-07321332) protects Syrian hamsters against infection with SARS-CoV-2 variants of concern. *Nat. Commun.* **13**, 719 (2022). doi:10.1038/s41467-022-28354-0 Medline

94. L. J. Reed, H. Muench, A simple method of estimating fifty per cent endpoints. *Am. J. Epidemiol.* **27**, 493–497 (1938). doi:10.1093/oxfordjournals.aje.a118408

95. J. Tan, B. K. Sack, D. Oyen, I. Zenklusen, L. Piccoli, S. Barbieri, M. Foglierini, C. S. Fregni, J. Marcandalli, S. Jongo, S. Abdulla, L. Perez, G. Corradin, L. Varani, F. Sallusto, B. K. L. Sim, S. L. Hoffman, S. H. I. Kappe, C. Daubenberger, I. A. Wilson, A. Lanzavecchia, A public antibody lineage that potently inhibits malaria infection through dual binding to the circumsporozoite protein. *Nat. Med.* **24**, 401–407 (2018). doi:10.1038/nm.4513 Medline

96. R. Abdelnabi, R. Boudewijns, C. S. Foo, L. Seldeslachts, L. Sanchez-Felipe, X. Zhang, L. Delang, P. Maes, S. J. F. Kaptein, B. Weynand, G. Vande Velde, J. Neyts, K. Dallmeier, Comparing infectivity and virulence of emerging SARS-CoV-2 variants in Syrian hamsters. *EBioMedicine* **68**, 103403 (2021). doi:10.1016/j.ebiom.2021.103403 Medline

97. E. F. Pettersen, T. D. Goddard, C. C. Huang, G. S. Couch, D. M. Greenblatt, E. C. Meng, T. E. Ferrin, UCSF Chimera—A visualization system for exploratory research and analysis. *J. Comput. Chem.* **25**, 1605–1612 (2004). doi:10.1002/jcc.20084 Medline

98. V. B. Chen, W. B. Arendall 3rd, J. J. Headd, D. A. Keedy, R. M. Immormino, G. J. Kapral, L. W. Murray, J. S. Richardson, D. C. Richardson, MolProbity: All-atom structure validation for macromolecular crystallography. *Acta Crystallogr. D Biol. Crystallogr.* **66**, 12–21 (2010). doi:10.1107/S0907444909042073 Medline

99. T. D. Goddard, C. C. Huang, E. C. Meng, E. F. Pettersen, G. S. Couch, J. H. Morris, T. E. Ferrin, UCSF ChimeraX: Meeting modern challenges in visualization and analysis. *Protein Sci.* **27**, 14–25 (2018). doi:10.1002/pro.3235 Medline

100. D. Pinto, E. Montani, M. Bolli, G. Garavaglia, F. Sallusto, A. Lanzavecchia, D. Jarrossay, A functional BCR in human IgA and IgM plasma cells. *Blood* **121**, 4110–4114 (2013). doi:10.1182/blood-2012-09-459289 Medline

101. X. Y. Ge, J. L. Li, X.-L. Yang, A. A. Chmura, G. Zhu, J. H. Epstein, J. K. Mazet, B. Hu, W. Zhang, C. Peng, Y. J. Zhang, C. M. Luo, B. Tan, N. Wang, Y. Zhu, G. Crameri, S. Y. Zhang, L. F. Wang, P. Daszak, Z. L. Shi, Isolation and characterization of a bat SARS-like coronavirus that uses the ACE2 receptor. *Nature* **503**, 535–538 (2013). doi:10.1038/nature12711 Medline

102. R. Vlasak, W. Luytjes, W. Spaan, P. Palese, Human and bovine coronaviruses recognize sialic acid-containing receptors similar to those of influenza C viruses. *Proc. Natl. Acad. Sci. U.S.A.* **85**, 4526–4529 (1988). doi:10.1073/pnas.85.12.4526 Medline

103. X. Huang, W. Dong, A. Milewska, A. Golda, Y. Qi, Q. K. Zhu, W. A. Marasco, R. S. Baric, A. C. Sims, K. Pyrc, W. Li, J. Sui, Human coronavirus HKU1 spike protein uses O-acetylated sialic acid as an attachment receptor determinant and employs hemagglutinin-esterase protein as a receptor-destroying enzyme. *J. Virol.* **89**, 7202–7213 (2015). doi:10.1128/JVI.00854-15 Medline

104. I. G. Madu, V. C. Chu, H. Lee, A. D. Regan, B. E. Bauman, G. R. Whittaker, Heparan sulfate is a selective attachment factor for the avian coronavirus infectious bronchitis virus Beaudette. *Avian Dis.* **51**, 45–51 (2007). doi:10.1637/0005-2086(2007)051[0045:HSIASA]2.0.CO;2 Medline

105. C. Winter, C. Schwegmann-Weßels, D. Cavanagh, U. Neumann, G. Herrler, Sialic acid is a receptor determinant for infection of cells by avian Infectious bronchitis virus. *J. Gen. Virol.* **87**, 1209–1216 (2006). doi:10.1099/vir.0.81651-0 Medline

106. W. Li, R. J. G. Hulswit, S. P. Kenney, I. Widjaja, K. Jung, M. A. Alhamo, B. van Dieren, F. J. M. van Kuppeveld, L. J. Saif, B.-J. Bosch, Broad receptor engagement of an emerging global coronavirus may potentiate its diverse cross-species transmissibility. *Proc. Natl. Acad. Sci. U.S.A.* **115**, E5135–E5143 (2018). doi:10.1073/pnas.1802879115 Medline
